# *In vivo* reconstruction of the cell lineage history of a developing mouse with DNA Typewriter, from zygote to late organogenesis

**DOI:** 10.64898/2026.07.29.741625

**Published:** 2026-07-30

**Authors:** Qi Yu, Haedong Kim, Sophie Seidel, James F. Acosta-Clark, Beth K. Martin, Kyle O’Connor, Riza M. Daza, Molly Gasperini, Jenny F. Nathans, Maggie Lam, Elena Gamo, Shruthi VijayKumar, Lauren Kuo, Jean-Benoît Lalanne, Kamen Simeonov, Marion Pepper, Cole Trapnell, Jesse M. Gray, Junhong Choi, Chengxiang Qiu, Jay Shendure

**Affiliations:** 1 Department of Genome Sciences, University of Washington, Seattle, WA, USA; 2 Seattle Hub for Synthetic Biology, Seattle, WA, USA; 3 Howard Hughes Medical Institute, Seattle, WA, USA; 4 Allen Institute, Seattle, WA, USA; 5 Medical Scientist Training Program, University of Washington, Seattle, WA, USA; 6 Present address: Dépt. de Biochimie et Médecine Moléculaire & Courtois Institute for Biomedical Innovation, Université de Montréal, Montréal, QC, Canada; 7 Public Health Sciences Division/Translational Research Program, Fred Hutchinson Cancer Center, Seattle, WA, USA; 8 Department of Immunology, University of Washington, Seattle, WA, USA; 9 Brotman Baty Institute for Precision Medicine, Seattle, WA, USA; 10 Institute of Stem Cell and Regenerative Medicine, University of Washington, Seattle, WA, USA; 11 Allen Discovery Center for Cell Lineage Tracing, Seattle, WA, USA; 12 Present address: Dev. Biology Program, Memorial Sloan Kettering Cancer Center, New York, NY, USA; 13 Department of Molecular and Systems Biology, Dartmouth College, Hanover, NH, USA

## Abstract

The complete cell lineage of *C. elegans*, mapped over four decades ago, was tractable because the animal is small, transparent, and lineage invariant^1^. Most animals are none of these, having orders of magnitude more cells, being opaque, and developing with substantial stochasticity^2^. Since 2016, genome editing-based lineage tracing has opened the door to dense cell lineage reconstruction in such organisms^3–8^, but delivering on that promise has proven technically challenging. Here we apply DNA Typewriter^9–11^, a prime editing-based recorder that writes stochastic symbolic insertions to an engineered genomic TAPE in strictly sequential order, to trace mouse development from zygote (E0) to late organogenesis (E13.5). We introduce constructs encoding a prime editor, engineered prime editing guide RNAs (epegRNAs), and Pol-III-driven circularized TAPE RNA (circTAPE) into wildtype zygotes by pronuclear injection (PNI), then assay embryos by single-nucleus transcriptional profiling (sci-RNA-seq3)^12^ with paired circTAPE recovery. From one E13.5 embryo bearing ~7 integrations of a constitutively expressed prime editor and 11 integrations of 6-unit circTAPE, we recover ~1.75M single-nucleus transcriptomes and reconstruct a time-calibrated phylogeny of 1,340,794 annotated cells with parsimony-based node support. The first cell division is marked unequivocally, and although the resulting blastomeres contribute asymmetrically to the embryo proper, they are fate-neutral and serve as internal replicates that reproduce every finding. A modest cohort of pre-gastrulation founders dominates the embryo, with inequality exceeding neutral expectation within one to two cell cycles of founder allocation, yet these founders remain broadly multipotent; a second phase of clonal dominance arises in specific lineages during organogenesis. At the finest scale, sibling cells share cell type 9-fold in excess of chance, reaching 68- to 107-fold for cell types arising from spatially restricted founder pools, while the recent differentiations of organogenesis are legible in the heterotypic structure of terminal clades. From clade co-occurrence alone, we recover germ-layer organization and a dated hierarchy of cell-type couplings with branch points from E8.5 onwards. Finally, by integrating these data with our single-cell time-series of mouse development^13^, we impute transcriptional states and annotations for the majority of internal nodes and recover established state histories for diverse cell types. All data are made freely available, together with NextCell, an interactive browser for this annotated cellular phylogeny of mouse development from zygote to late organogenesis.

## INTRODUCTION

In the 1970s and early 1980s, John Sulston and colleagues watched *C. elegans* develop under the microscope, painstakingly drawing each cell division until they had mapped its complete cell lineage, from zygote to adult^1,2^. It remains one of the singular accomplishments of modern biology -- every somatic cell of the developing worm, placed on a single, enduring tree. More than forty years on, we have achieved nothing remotely comparable for any other model organism.

One reason that Sulston and colleagues succeeded is that *C. elegans* is, in several key respects, exceptional. It is transparent, allowing cells to be followed directly by eye. It is small, with fewer than 1,000 somatic cells in the adult hermaphrodite. And, most fundamentally, its development is deterministic, unfolding identically in every individual, such that observations made in one individual are readily integrated with those made in others. In contrast, nearly all animals, including all mammals, develop in the opposite, regulative mode. Fates are specified largely by extrinsic cues, such as cell-cell interactions and positional signals, and not by lineage alone; the embryo accommodates displaced or missing cells; and the precise sequence of divisions varies between individuals. Lineage is nonetheless far from irrelevant, because a cell inherits from its mother not only its genome but its position, its neighbors, and its molecular state. Extrinsic cues are therefore themselves substantially heritable, and lineage and environment act as correlated rather than competing determinants of fate. The question is thus not whether fate follows lineage, but how much of it does, in which lineages, and by when. The defining feature of regulative development is the emergence of reproducible form and function from a process that, at the level of individual cells, is substantially but not wholly stochastic -- a reconciliation that no single invariant lineage can capture.

Beyond developing regulatively, mammals present practical obstacles that compound the challenge of dense lineage measurement at every turn. They are opaque, foreclosing the direct visual observation that served Sulston so well. They are also vastly larger. An adult mouse contains billions, and an adult human trillions, of nucleated cells^3^, placing the tracking of individual cells far beyond the reach of the eye or the microscope. In the absence of direct observation, our principal recourse has been to sample, *e.g.* dissociating an embryo at a given stage and profiling the molecular states of its cells. For instance, we and others have leveraged such snapshots to generate rich, whole-embryo, temporally resolved scRNA-seq and scATAC-seq atlases of mouse development^4–9^. But sampling is destructive, capturing each cell at a single instant and severing it from both its past and future. Cell lineage relationships, as well as the spatial and state trajectories of individual cells, must then be inferred, indirectly and with substantial ambiguity, by stitching together snapshots acquired from different individuals. This inference is precisely what regulative development frustrates, since no two individuals share the same sequence of cell divisions. Furthermore, it does not scale. Constructing a high-resolution temporal atlas is a major undertaking for even a single wildtype genotype, let alone for each of the thousands of mutants in which the developmental origins of a phenotype are of interest.

The fact that the genome is a digital, engineerable medium that is faithfully copied at each cell division makes it natural to consider exploiting it for recording cell lineage. Numerous studies have sought to leverage somatic genetic tags to recover lineage information, including naturally arising somatic mutations^10,11^ or epimutations^12^, static clonal barcodes^13^, and site-specific recombination-mediated diversification^14–17^. However, these strategies are limited in their potential for multiplexing, for stable recording over long timespans, and/or for the efficient recovery of recorded information from vast numbers of cells^18^.

Around 2016, these limitations led us and others to pursue a paradigm in which genome editors such as CRISPR/Cas9 are leveraged to enable organisms to record their own histories via “evolving barcodes”^18–23^. The core premise is to introduce the necessary components for genome editing (*i.e.* a genome editor, guide RNAs, and target sites) to a zygote, such that diverse, heritable marks accumulate continuously at defined genomic locations throughout development. When read out at an endpoint via single-cell or single-nucleus molecular profiling of both endogenous transcripts and transcribed evolving barcodes^24^, a single destructive snapshot becomes informative not only of each cell’s present state but also of its past. This collapses the inference problem, as cell lineage (and potentially other aspects of cells’ histories) can be inferred from the accumulated edits observed in cells sampled from one individual rather than reconstructed across individuals.

Although diverse implementations of this basic idea have been pursued over the past decade, they can be broadly grouped by the class of genome editor employed. A first group uses CRISPR-Cas9 nucleases to generate double-strand breaks at arrays of target sites during development, such that error-prone repair leaves heritable, lineage-informative insertions and deletions (“scars”). This approach powered the earliest genome editor-based recorders in zebrafish, mammalian stem cells, and mice (*e.g.* GESTALT^19^, MEMOIR^20^, scGESTALT^24^, ScarTrace^25^, LINNAEUS^26^, MARC1^21^, Chan *et al.*^22^, CARLIN^27^). A second group uses base editors, which install point mutations without double-strand breaks at sites that can be densely packed into compact arrays (*e.g.* SMALT^28^, baseMEMOIR^29^, BASELINE^30^, Hypercascade^31^). A third group uses prime editors^32^, which write short, programmed insertions at target sites via a Cas9 nickase fused to a reverse transcriptase (*e.g.* DNA Typewriter^33^, peCHYRON^34^, PEtracer^35^).

Substantial progress has been made over the course of this paradigm’s first decade. However, the reconstruction of dense *in vivo* cell lineages has been a persistent struggle, for a confluence of technical reasons^18^, including: (i) Delivery and expression. The full complement of recording components must be introduced into the zygote and expressed stably throughout development, without silencing, mosaicism, or the toxicity and lethality that excessive editing can potentially cause; (ii) Information loss. For Cas9-based recorders, when target sites are packed densely into arrays, the double-strand breaks that drive editing frequently trigger deletions that span and destroy neighboring sites, erasing information that has already been written; (iii) Instability of temporal resolution. For most strategies, recording sites are consumed as they are written to, such that it is challenging to maintain a stable recording resolution over long periods of time; (iv) Event ordering. Most systems record only unordered marks, so the temporal sequence of events must be reconstructed solely by phylogenetic inference rather than read directly from the genome; (v) Completeness. Dense phylogenetic reconstruction requires profiling of a substantial proportion of an organism’s cells. There are on the order of 100M cells in a newborn mouse^5^, roughly on par with the aggregate output of the entire scRNA-seq field to date^36^; (vi) Records retrieval. The recovery of all evolving barcodes from each profiled cell or nucleus is a challenging technical requirement; (vii) Tree building. Algorithms for inferring cell lineage trees have largely been developed and benchmarked on 10^2^ to 10^4^ cells, orders of magnitude below the ~10^7^ cells of a mid-gestational mouse embryo, let alone the ~10^9^ cells of an adult mouse; at such scales exhaustive tree search and resampling-based measures of node support are computationally infeasible; and (viii) Design-test cycle time. Optimizing *in vivo* recording systems is slow (because *in vivo* work is generally slow in large model organisms), throttling the pace of technology development.

**Figure 1.**
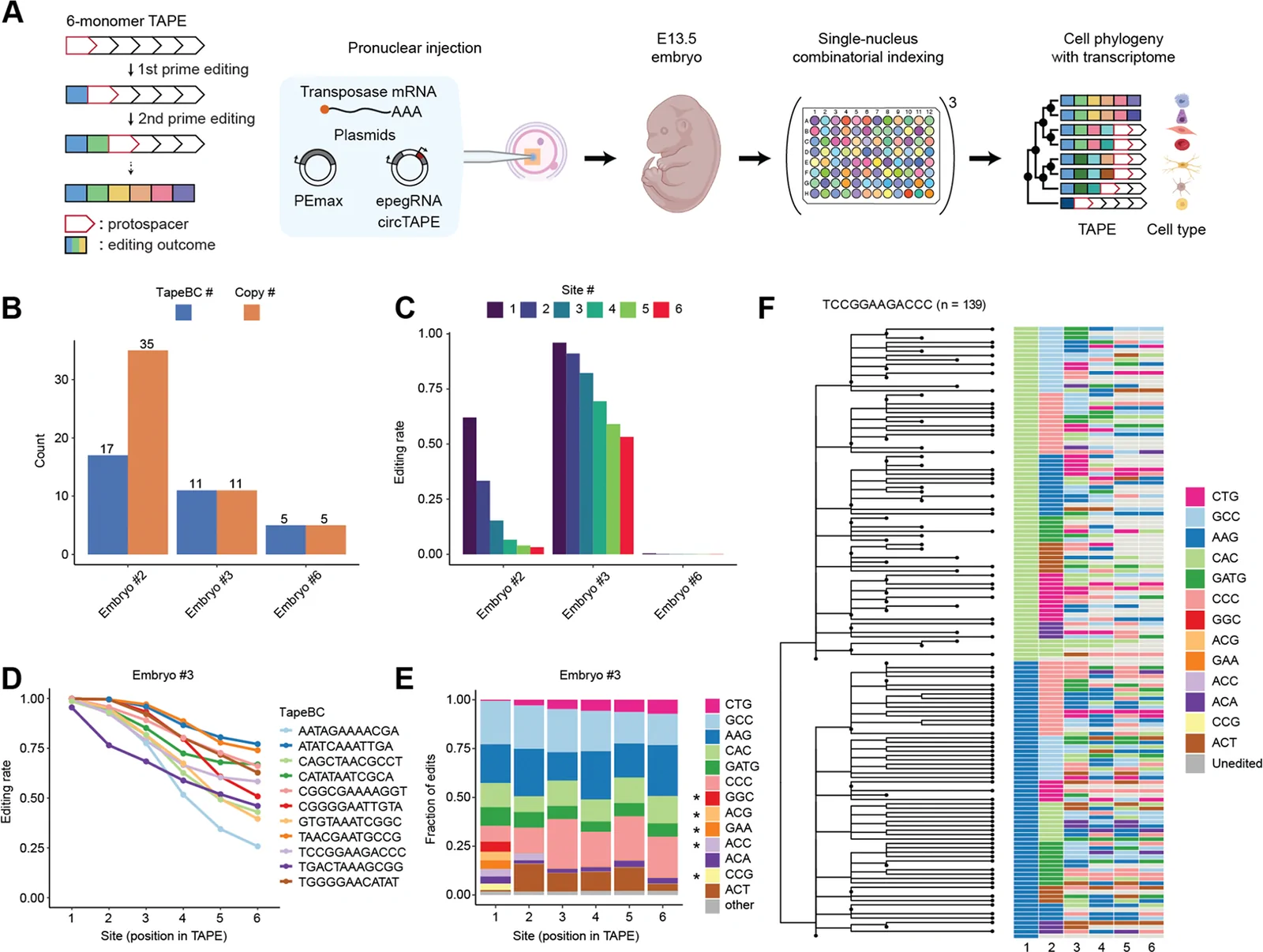
Pronuclear zygotic injection (PNI) of DNA Typewriter components identifies an embryo with robust levels of sequential editing by E13.5. **(A)** Schematic. Left: a 6-monomer TAPE array is edited sequentially by prime editing, with each event installing an insertional symbol (colored) at the active site while regenerating the protospacer (red outline) at the next. Middle: constructs encoding constitutively expressed PEmax, epegRNA, and circTAPE, together with piggyBac transposase mRNA, were delivered to the zygote by PNI. Right: embryos were harvested at E13.5 and transcriptionally profiled by three-level single-nucleus combinatorial indexing (sci-RNA-seq3)^40^, yielding paired circTAPE genotypes and transcriptomes for the reconstruction of a cell phylogeny annotated by cell type. **(B)** Number of distinct TAPE-BC sequences (unique integration barcodes; blue) and the estimated total number of genomic integrations after accounting for multi-copy barcodes (orange), shown separately for each of three E13.5 embryos bearing successful TAPE integrations. **(C)** Per-site editing rate for each embryo, expressed as the fraction of resolved reads edited at each of the six monomer sites (colored 1–6), collapsed with respect to independent integrations but shown separately for the three TAPE-bearing embryos. Editing is low-to-absent in embryo #6, modest in embryo #2, and extensive in embryo #3. **(D)** Per-site editing rate in embryo #3, shown separately for each of 11 single-copy TAPE-BC integrations. Editing declines progressively from site 1 to site 6, consistent with sequential writing. **(E)** Frequency of each insertional symbol at each of the six sites within TAPE, aggregated across integrations in embryo #3. The 13 symbols present at ≥0.5% frequency at one or more sites are shown individually; the remainder are grouped as “other”. The 5 symbols that are present at ≥0.5% frequency only at site-1 and/or site-2 are marked with an asterisk. **(F)** Neighbor-joining lineage tree reconstructed from the lineage genotypes of a single representative integration (TAPE-BC “TCCGGAAGACCC”; n = 139 genotypes), with the corresponding six-site edit patterns shown at right (colored by symbol; grey, unedited).

DNA Typewriter^33,37^ addresses several of these limitations (Fig. 1A). Built on prime editing, a key feature of DNA Typewriter is that each edit installs an information-bearing insertion at a target site while simultaneously unlocking a new one. In principle, because the number of writable sites remains stable, the rate of recording should remain constant, rather than recording finely early on and progressively more coarsely as sites are exhausted. Because insertions are added strictly in sequence, their 5′ to 3′ linear order preserves the temporal order in which events were written, simplifying phylogenetic reconstruction. We established DNA Typewriter as a cell lineage recorder in cultured HEK293T cells^33^. We subsequently demonstrated its compatibility with the symbolic recording of signaling and *cis*-regulatory activity with ENGRAM^38^. We also recently applied DNA Typewriter to reconstruct a bootstrap-supported phylogeny of ~50,000 cells derived from ~100 monoclonal gastruloids, which were in turn derived from a single founding stem cell^39^. These gastruloids recapitulate aspects of early development, but remain an *in vitro* model.

Here we apply DNA Typewriter to record the *in vivo* cell lineage of a mouse. Introducing all recording components into a wildtype zygote by pronuclear injection (PNI) and piggyBac integration, and reading them out at E13.5 via exponentially scalable single-nucleus transcriptional profiling (sci-RNA-seq3)^40^ with paired recovery of edited circTAPEs, we obtain a time-calibrated developmental phylogeny of a single embryo spanning its first cell division through late organogenesis, comprising a parsimony-supported backbone of 640,012 cells expanded by distance-based placement to 1,340,794 cells. This tree sheds quantitative, organism-wide light on the mouse embryo proper, including: clonal dominance in excess of neutral expectation, established within one to two cell cycles of founder allocation, in which a modest cohort of pre-gastrulation progenitors accounts for a disproportionate fraction of the embryo while remaining broadly multipotent; fate sharing among sibling cells far above chance, strongest in lineages that commit from spatially restricted progenitor pools; and a dated hierarchy of cell-type relationships, reconstructed from clonal co-occurrence alone, that recovers the major progenitor fields. Finally, by integrating these data with a transcriptional time-series of mouse development^5^, we impute transcriptional states and annotations for the majority of internal nodes and chart plausible, lineage-informed state histories for the origins of cell types. All data are made available, alongside NextCell, an interactive website for exploring the annotated cell lineage tree.

## RESULTS

### In vivo DNA Typewriter recording to expressed, circularized TAPE

DNA Typewriter relies on three components: (i) a prime editor, (ii) epegRNAs, and (iii) TAPE (Fig. 1A; fig. S1A)^33,37^. Blank TAPE is composed of a tandem monomer array (here, 6 × 14 bp), preceded at its 5′ end by a key sequence (here, GGA). This key, together with the first monomer and the first portion of the second, forms a 20-bp protospacer recognized by the epegRNAs. A successful prime editing event installs an information-bearing insertion (here, NNNGGA) that disrupts the original protospacer while also converting the immediately downstream monomer into a new, complete protospacer. This sequential editing feature enables genetic marks to be installed in an ordered manner and keeps the number of editable sites per TAPE constant at one, at least until the end of the tandem array is reached.

Toward an *in vivo* proof-of-concept of DNA Typewriter, we sought to introduce constitutively expressed PEmax^41^ (prime editor), together with a library encoding an epegRNA programming a partially degenerate insertion (NNNGGA), a 12-bp barcode (TAPE-BC: NNNNNAANNNNN), and blank TAPE (6 monomeric units), into wildtype mice via PNI and piggyBac-mediated integration (Fig. 1A; fig. S1A). A critical technical challenge, however, relates to records retrieval. The original TAPE was designed to be transcribed as a protein-coding mRNA^33^, but achieving high-efficiency TAPE recovery in scRNA-seq has proven persistently difficult^39^. This challenge is compounded during midgestational organogenesis by the formation of bone, cartilage, and other differentiated tissues, which makes whole-embryo cellular dissociation difficult to achieve without introducing cell type biases. We have previously circumvented this, through as late as P0, by profiling nuclei rather than whole cells^6,40,42^. However, recovering an mRNA reporter from nuclei with minimal dropout is harder still, simply because far less mRNA is present there.

We recently showed that a Pol-III-driven circular RNA reporter exhibits 160-fold higher recovery in cells than its linear equivalent in a single-cell quantitative expression reporter assay, consistent with the enhanced stability conferred by the absence of free termini^43,44^. We therefore engineered a version of TAPE that leverages Pol-III transcription and the Tornado system to express circularized TAPE transcripts (“circTAPE”) at high levels. The circTAPE additionally bears a 30-nt poly(A) region and a custom capture sequence, enabling its oligo-dT-based and (optionally) targeted reverse-transcription, respectively (fig. S1A).

To generate DNA Typewriter embryos, we performed PNI of the PEmax and epegRNA∷TAPE-BC∷circTAPE constructs described above, together with mRNA encoding the piggyBac transposase and synthesized with a Cap-1 structure, a poly(A) tail, and *N*^1^-methyl-pseudouridine (m^1^Ψ) modification to enhance mRNA stability and translational efficiency, into isogenic C57BL/6J (B6) zygotes (Fig. 1A; fig. S1B). Of 100 injected zygotes, 78 were immediately transferred at the 1-cell stage into the oviducts of three pseudo-pregnant CD-1 recipient females, all of which became pregnant. Following harvesting and snap freezing at E13.5, we isolated nuclei from 10 embryos as previously described^5,40^.

### Recorded clonal diversity in embryo #3 (E13.5) is extensive, tree-like and far from saturated

To count TAPE integrations and quantify any DNA Typewriter activity, we performed droplet digital PCR (ddPCR) and bulk amplicon sequencing of epegRNA∷TAPE-BC∷circTAPE integrations from 5 ng of genomic DNA per embryo. In 3 of the 10 embryos, we identified 17 (embryo #2), 11 (embryo #3), and 5 (embryo #6) distinct TAPE-BC sequences (Fig. 1B). However, per-barcode read-count distributions indicated that in embryo #2, some of its 17 barcodes were present in multiple copies (consistent with piggyBac excision and re-integration, which we also observed in high-MOI cell culture^45^), such that the actual number of integrations was ~35 (Fig. 1B; fig. S1C). Parsing the six sites of each TAPE array, we detected low-to-absent DNA Typewriter activity in embryo #6, modest activity in embryo #2, and high activity in embryo #3 (Fig. 1C). To understand this variation between embryos, we quantified PEmax copy number by ddPCR (fig. S1D). PEmax was detected in embryos #2 (~2 copies) and #3 (~7 copies), the same TAPE-bearing embryos in which we observed modest and substantial editing, respectively.

Given the substantial activity of DNA Typewriter in embryo #3, we decided to focus solely on it. The rate of NNNGGA insertions declined progressively across the six monomers, from 96% at site-1 (95–100% across the 11 integrations) to 53% at site-6 (26–77% across integrations), consistent with the 5′ → 3′ sequential editing expected of DNA Typewriter (Fig. 1D). Our estimate of 11 single-copy integrations of the epegRNA∷TAPE-BC∷circTAPE construct led us to expect a “vocabulary” of no more than 11 distinct 3-nt symbols (insertions). Interestingly, we observed 13 symbols present at ≥0.5% frequency at one or more of the sites within each six-unit TAPE, including 12 at site-1, 9 at site-2 and 8 at each of sites 3–6 (Fig. 1E). To understand this better, we performed amplicon sequencing of the full epegRNA::TAPE-BC∷circTAPE integrations, which recovered 10 of the 11 TAPE integrations and showed them to collectively encode all 8 insertions present at ≥0.5% at each of the six sites (7 × 3-nt and 1 × 4-nt). Because a complex library bearing epegRNAs encoding all possible 3-nt insertions was injected into the zygote, and only 11 of these integrated, the additional symbols at sites 1–2 were likely mediated by epegRNAs transiently expressed shortly after PNI, and are thus restricted to the 5′-most sites.

As DNA Typewriter records both sequentially and combinatorially, even a single TAPE integration can in principle uniquely mark a large number of distinct lineages (*e.g.* with 8 symbols, there are 8^6^ (~10^5^) configurations for a fully edited, six-site TAPE). To assess how much of this potential diversity was realized *in vivo* in embryo #3, we performed a rarefaction analysis on the “lineage genotypes” observed at each of the 11 integrations. Because the bulk amplicon data lack UMIs, we estimated genotype abundances from read depth. For each integration, we took λ, the read depth corresponding to a single genome equivalent, as the mode of the per-genotype read-count distribution in log space, and estimated the number of genome equivalents (cells) carrying a given genotype as its read count divided by λ. Treating these per-genotype genome-equivalent counts as abundances, we computed the accumulation of distinct genotypes as a function of genome equivalents sampled (Hurlbert interpolation^46^; fig. S1E).

Each integration was represented by an estimated 180–370 genome equivalents, distributed across an observed 76–165 distinct lineage genotypes. Moreover, every integration lay on the steeply rising portion of its accumulation curve, with a terminal slope far from zero. This regime is set by the input itself, in that the ~5 ng of genomic DNA assayed corresponds to only ~850 genome equivalents (~6 pg of gDNA per cell), and likely fewer, as UV quantitation tends to overestimate DNA mass^47^. The observed richness of lineage genotypes at even a single TAPE in embryo #3 is thus a substantial fraction of the genomes sampled, indicating that the diversity we recover is limited by input material rather than recorder capacity, and that deeper sampling would reveal many more lineage genotypes.

Finally, we reconstructed phylogenetic trees from the lineage genotypes observed at each integration. For each, we computed a DNA Typewriter-specific distance metric^48^ that respects the ordered, monotonic nature of its write events, and applied Neighbor Joining (NJ)^49^ with midpoint rooting. All 11 integrations yielded clean, tree-like structures (Fig. 1F; fig. S2). Notably, for 9 of the 11 TAPEs, >95% of lineage genotypes bore one of only two symbols at site-1. For example, for integration “TCCGGAAGACCC”, 139 of 139 lineage genotypes bore either CAC (55%) or AAG (45%) at site-1, while for integration “CATATAATCGCA”, 122 of 122 lineage genotypes bore either ACA (53%) or AAG (47%) at site-1. A parsimonious explanation is that there was a pronounced burst of editing activity coincident with zygotic genome activation at the 2-cell stage, unambiguously marking the first cell division.

Together, these results establish that DNA Typewriter, when robustly established in a wildtype mouse zygote by PNI and piggyBac integration of all components, records heritable, ordered, lineage-informative marks *in vivo*, including marking the daughters of the first cell cleavage. Analyzing each integration in isolation, however, captures only a fraction of the available information. Because the 11 × 6-unit TAPEs were edited independently, the lineage history of any given cell is defined by the combination of lineage genotypes across all of them. Read jointly, the theoretical upper bound on the number of possible configurations expands from 8^6^ (~10^5^) per integration to 8^(6*11)^ (~10^59^) per cell. However, realizing this potential requires determining which TAPE sequences co-occur within individual cells.

### Lineage-informative single-nucleus RNA-seq of an E13.5 DNA Typewriter mouse embryo

We therefore subjected all ~10M remaining nuclei isolated from embryo #3 to transcriptional profiling with three-level single nucleus combinatorial indexing (sci-RNA-seq3)^40^, with minor modifications to enhance the recovery of expressed circTAPEs both to establish a lineage genotype for each cell and to pair it with that cell’s transcriptional state (Fig. 1A). Specifically, during the third round of barcoding, distinct primer sets were used for indexed, UMI-tagged PCR amplifications of transcriptome and TAPE libraries. These libraries were sequenced (Illumina NextSeq, NovaSeq) to obtain 8.5B and 1.6B reads for the transcriptome and circTAPE libraries, respectively. For the transcriptome library, reads were demultiplexed, adapter-trimmed, and aligned to the mouse reference genome (mm39). Following removal of PCR duplicates (40%-66%), low-quality nuclei and putative doublets, the final cell-by-gene count matrix comprised transcriptional profiles for 1,753,895 nuclei (median UMI count: 790; median genes detected: 583).

**Figure 2.**
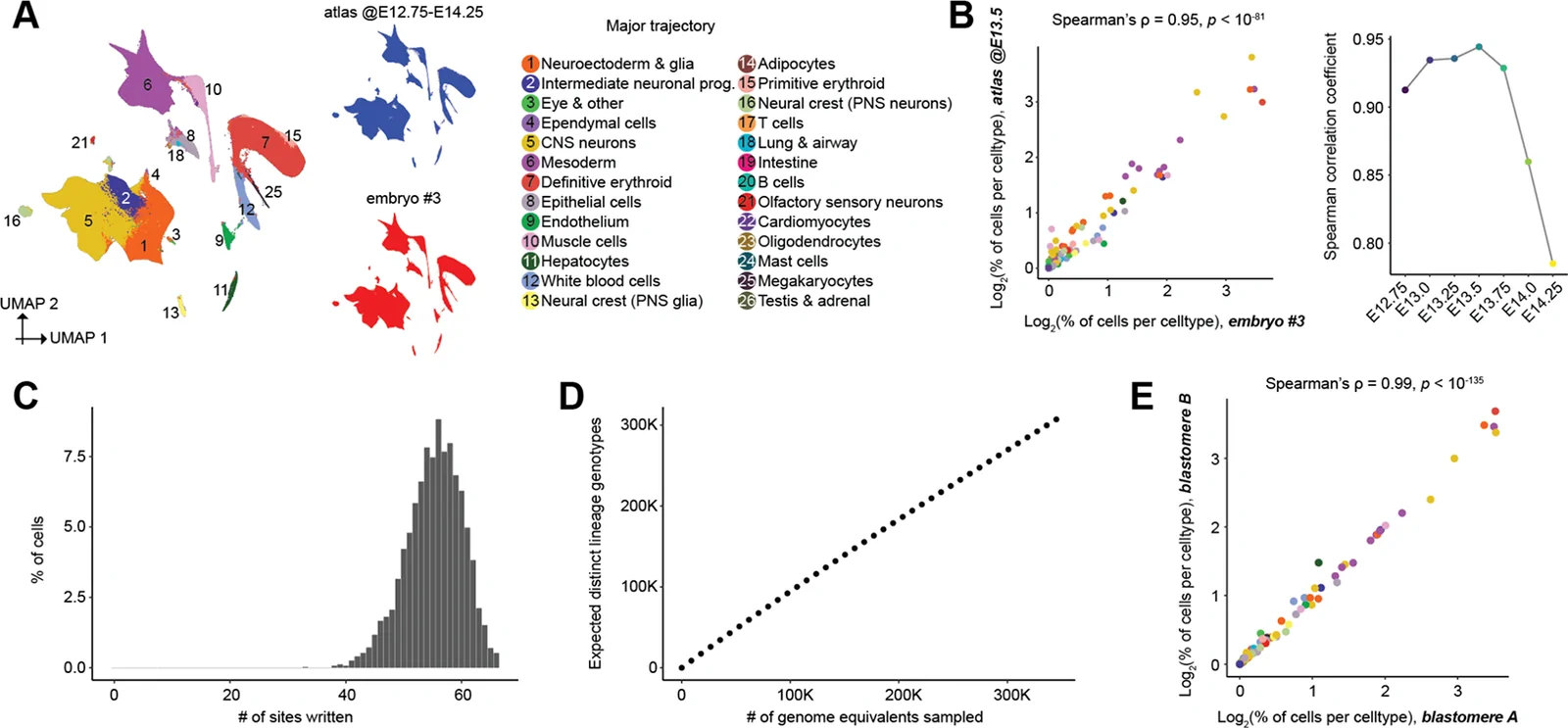
1.75M single-nucleus transcriptomes of a DNA Typewriter E13.5 embryo recapitulate wildtype E13.5 cell type proportions and carry extensive lineage recordings whose sampling remains unsaturated. **(A)** 2D UMAP co-embedding of 3.4M scRNA-seq profiles, including 1.75M from embryo #3 (this study) and 1.65M from 7 timepoints of our high-temporal-resolution single-cell atlas of mouse development (E12.75 - E14.25 in 6-hr increments)^5^. Left: all cells, colored by the 26 major trajectories (numbered key); major trajectories accounting for more than 0.1% of cells are labeled. Right: the same embedding split by dataset of origin, showing wildtype timecourse atlas cells (top) and embryo #3 cells (bottom). **(B)** Left: log_2_-scaled percentage of cells per cell type in DNA Typewriter E13.5 embryo #3 (x-axis) versus wildtype E13.5^5^ (y-axis), for each of the 169 cell types detected among the 3.4M cells shown in panel **A**. Each point is one cell type, colored by its major trajectory (Spearman’s ρ = 0.95, p < 10^−81^). Right: Spearman’s ρ between cell type proportions in embryo #3 versus each wildtype atlas timepoint from E12.75 to E14.25. Concordance peaks at E13.5, consistent with the intended harvest stage. **(C)** Histogram of the number of edited sites per cell in embryo #3, for the 4,027 cells with consensus genotypes at all 11 TAPE integrations. The distribution is unimodal, and on average, 55 ± 5 sites per cell were written, out of the 11 × 6 = 66 available sites. **(D)** Cell-level rarefaction analysis of lineage-genotype diversity, based on the 345,140 cells with a consensus genotype call for all 5 of the most highly recovered circTAPE integrations. The expected number of distinct lineage genotypes is plotted against the number of cells sampled (Hurlbert interpolation). The curve remains near-linear. **(E)** Log_2_-scaled percentages of cells per cell type in blastomere A (975,196 cells; x-axis) versus blastomere B (711,668 cells; y-axis), for the 135 cell types detected in the union of A and B. Each point is one cell type, colored by its major trajectory (Spearman’s ρ = 0.99, p < 10^−135^).

For cell type annotation, we turned to our 11.4M-cell single-cell transcriptional atlas of mouse development, which was staged at 2- to 6-hour intervals spanning late gastrulation (E8) to birth (P0)^5^. We selected 7 timepoints spanning E12.75 to E14.25 and co-embedded these 1.65M cellular profiles with the 1.75M cellular profiles of embryo #3 in a shared 30-dimensional PCA space (Fig. 2A). Embryo #3 cells were assigned major trajectory and cell type labels based on their k-nearest neighbors (kNN, k = 20) from the atlas. The resulting cell type annotations and their proportions in embryo #3 were highly consistent with those observed at E13.5 in the atlas (Spearman’s ρ for log_2_-scaled proportions: 0.95, p < 10^−81^; Jensen-Shannon divergence (log_2_): 0.03; Fig. 2B). Moreover, this concordance was maximal at E13.5, consistent with the intended harvest stage and no overt impact of the ~18 piggyBac-mediated integrations on embryonic development (Fig. 2B).

For the circTAPE library, we assigned reads to cells, validated them against the bulk-derived integration whitelist and edit vocabulary, collapsed and error-corrected by UMI (requiring ≥2 reads per UMI), de-noised (including chimera removal), and called a single consensus lineage genotype per cell per circTAPE, requiring the dominant lineage genotype to be supported by ≥3 UMIs and to account for ≥90% of the cell’s UMIs at that integration. We recovered a median of 45 quality-passing circTAPE UMIs per cell (IQR 23–77). Overall, 49.6% of the ~1.75M cell × 11 TAPE matrix bore a genotype call. Per-integration recovery ranged from 45% to 66% for 9 of the 11 TAPEs, but was only 20% and 3% for the remaining 2 TAPEs. We attribute this to epigenetic silencing of a subset of circTAPE-encoding integrations.

To what extent was the recording capacity of DNA Typewriter in this mouse (*i.e.* 11 integrations × 6 monomers = 66 writable sites) saturated by E13.5? We first restricted the analysis to the 4,027 cells with a consensus genotype at all 11 TAPEs. On average 55 ± 5 sites per cell had been written (84%), with a median of 7 of the 11 TAPEs exhausted (*i.e.* all six monomers used). The distribution of written sites per cell was unimodal and peaked at 56; every cell had written at least half of its 66 sites, but only 0.5% (21 cells) had written all of them (Fig. 2C). Because these 4,027 cells are just 0.23% of the dataset and might be atypical, we repeated the estimate using the entire matrix. For each TAPE, we averaged the number of written sites across every cell in which that TAPE was genotyped (mean ~870,000 cells per TAPE), then summed these 11 per-TAPE means. This yielded an estimate of 53 of 66 sites overall (56 for blastomere A; 50 for blastomere B). Thus, by E13.5 the recording capacity had been >80% used but not saturated.

If DNA Typewriter recorded actively across a sustained window of development and its recording capacity was not exhausted by the time of harvest, we would expect the vast majority of cells to carry a unique lineage genotype. To quantify the extent to which this is true, we focused on cells for which a consensus genotype was called for all of the 5 most highly recovered circTAPEs and performed a rarefaction analysis on cell lineage genotypes (rather than on individual TAPEs as in fig. S1E). Among these 345,140 cells, only 17% shared an identical cell lineage genotype with another. Furthermore, the accumulation curve was near-linear, with nearly every additional cell contributing a previously unobserved cell lineage genotype (Fig. 2D). This implies that our resolution is limited by the number of cells sampled, rather than by the extent of *in vivo* recording activity of DNA Typewriter in this experiment. Reassuringly, of 13,610 pairs of cells sharing an exact genotype across these five TAPEs, 57% were of the same cell type, 10-fold more than the 5.7% expected by chance -- an early indication that cell lineage genotype captures biologically meaningful structure. Having established this, we proceeded to phylogenetic reconstruction.

### Recording dynamics shape the dating and branch support of a 640,012-cell backbone phylogeny

The pronounced burst of editing following PNI marked the two blastomeres resulting from the first mitotic division with numerous unique edits (Fig. 1F; fig. S2), enabling us to separate their descendants directly, rather than relying on tree-building to resolve the first split. We refer to these 2-cell stage blastomeres as “A” and “B” throughout the remainder of this report. From 385,267 cells with ≥8 genotyped TAPEs, we derived the edit state of A and B at each TAPE (A: 23 edits, B: 19 edits; **table S1**). We then assigned 1,686,864 of 1,708,269 cells with ≥1 genotyped TAPE (98.7%) to either blastomere A (57.1%) or B (41.7%). The remaining 1.3% lacked a resolvable founder signature and were set aside. Because founder signatures are derived from well-covered cells, this partition is robust to the coverage differences that can mislead distance-based splitting.

Although blastomere A contributed 1.37-fold more cells to the embryo than B, their cell-type compositions were highly correlated (Spearman’s ρ for log_2_-scaled proportions: 0.99, p < 10^−135^; Jensen-Shannon divergence (log_2_): 0.003; Fig. 2E), indicating that the first division is clonally asymmetric in output but fate-neutral. We exploit this clean, fate-neutral separation throughout the remainder of this report by reconstructing the cell lineage of A and B independently, essentially treating the two daughters of the first cleavage as internal replicates drawn from the same embryo. Consequently, each phylogenetic pattern that we report can be measured twice and its reproducibility assessed directly.

The >10^6^ lineage-informative cells exceeded the practical limits of direct phylogenetic reconstruction, so we proceeded in two stages: (i) independent reconstruction of high-confidence backbone trees for blastomeres A and B from cells with ≥7 genotyped TAPEs, and (ii) distance-based placement of additional cells with ≥4 genotyped TAPEs onto those high-confidence backbones. To build the backbone trees, we computed distance matrices for 401,901 (A) and 238,111 (B) cells using a DNA Typewriter-specific metric that respects the sequential nature of events written to TAPE^48^, and then applied DecentTree’s scalable neighbor-joining (NJ) implementation^50–52^ to build two independent trees rooted on the defining edit state of A and B.

We independently dated the internal nodes of the A and B backbones with least-squares dating (LSD)^53^ under a strict molecular clock, calibrating each tree at two fixed points (root: E1.5, tips: E13.5), imposing a 2-hour minimum branch length (the fastest cell cycles of the gastrulating epiblast^54^), and constraining the number of ancestral lineages to no more than the number of cells present in the embryo (**table S2**). This ceiling is principled, in that a tree built from a subsample of cells can contain no more lineages at any given developmental time than the whole embryo then had cells. In practice, the ceiling binds only near gastrulation, where ceiling-free dating places far more lineages than the embryo has cells (fig. S3A; *e.g.* 90,820 at E6.5, against an epiblast of ~660 cells^54^), yet imposing it changes the least-squares objective by only +2.0% (A) and +1.8% (B). In both the A and B trees, imposing the ceiling shifted node ages later, by an amount that decreased with the unconstrained node date, *e.g.* +3–4.5 days for early nodes and ~0 for late nodes (fig. S3B).

**Figure 3.**
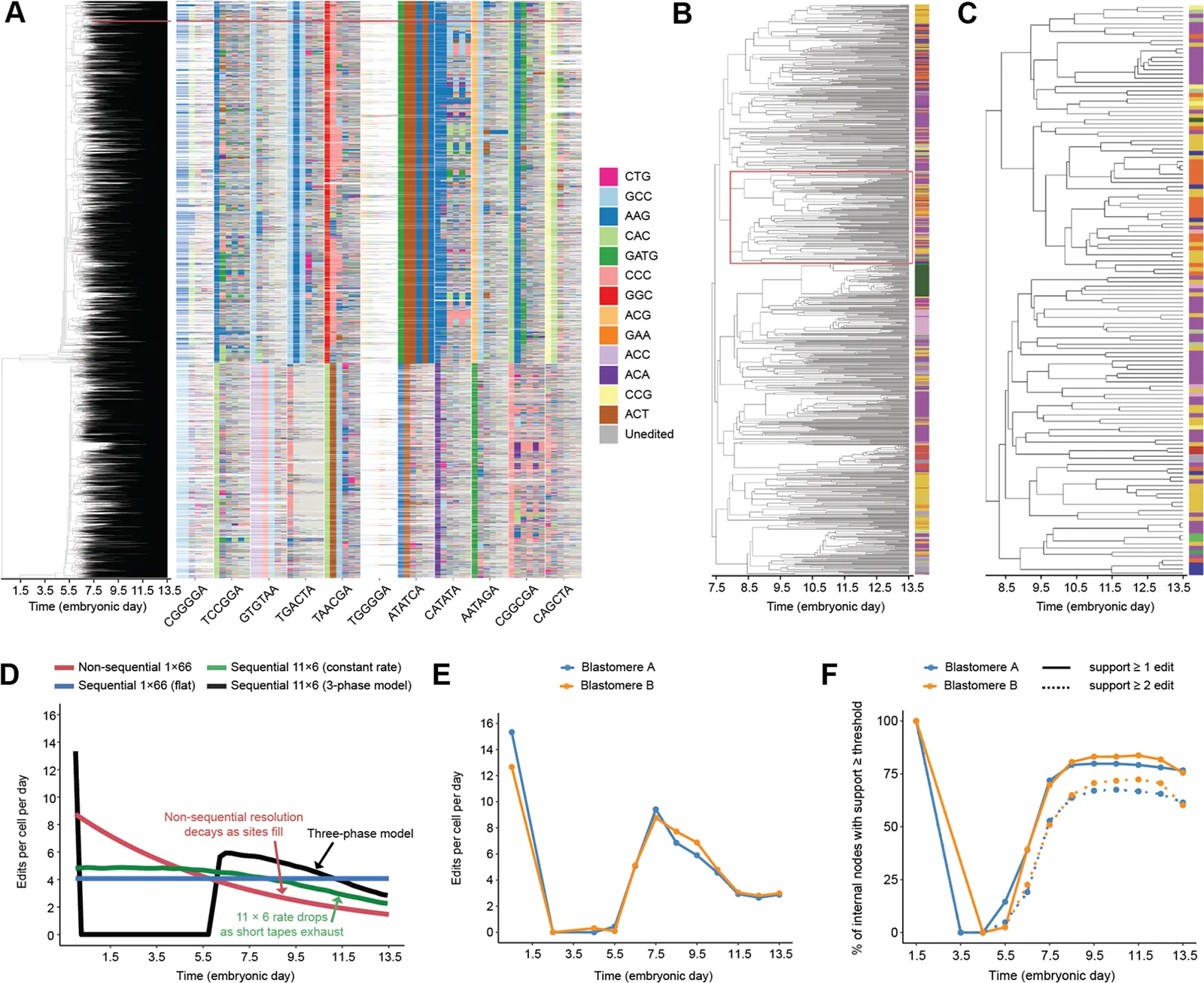
A dated, tip-annotated phylogeny of an E13.5 DNA Typewriter embryo, in which editing rate and branch support both track developmental time. **(A)** Timed lineage tree (640,012 cells; root fixed E0; A and B subtrees fixed at E1.5, tips fixed at E13.5). The tree (left) is shown beside the per-cell TAPEs (right). Each of the 11 TAPE integration sites contributes 6 editable monomer positions, colored by the observed edit outcome. **(B)** Zoom-in on clade outlined by red rectangle in panel **A** (845 cells; clade root at E7.38), plotted as a time-scaled tree with color-coded major trajectory annotations of tips at right (color legend in Fig. 2A). **(C)** Zoom-in on clade outlined by red rectangle in panel **B** (137 cells; clade root at E7.95), plotted as in panel **B**. **(D)** Mean edits accumulated per cell versus developmental time, for four idealized models, each tuned to reach 55 of 66 edits by E13.5: non-sequential 1 × 66-unit TAPE under a constant per-site, per-day editing rate (red), sequential 1 × 66-unit TAPE under a constant per-site, per-day editing rate (blue), sequential 11 × 6-unit TAPEs under a constant per-site, per-day editing rate (green), and sequential 11 × 6-unit TAPEs under a three-phase model comprising a 20-edit burst, quiescence until E6, and then a constant per-site, per-day editing rate (black). **(E)** Observed TAPE editing rate (edits per cell per day) across developmental time for blastomere A (blue; 401,901 cells) and blastomere B (orange; 238,111 cells). Trees were built by neighbor-joining on a DNA Typewriter-specific distance that counts the number of edits separating two cells given the ordered, irreversible nature of writing to TAPE^48^, then dated under a strict molecular clock with a 2-hour minimum branch length and a literature-informed ceiling on the number of coexisting lineages (**table S2**). Ancestral TAPE states were then inferred as the longest edit prefix shared by all descendants of each node, and branches assigned to 1-day bins from E0 to E14 by their temporal midpoint; the rate for each bin is the sum of edits across integrations divided by the total branch time falling in that bin. The peak in the E0-E1.0 bin (≈15 (A) and ≈13 (B) edits per day) corresponds to the founder burst, placed on each blastomere’s stem lineage. **(F)** Internal-node support across developmental time for blastomere A (blue) and B (orange), binned by node date over the same 1-day windows. Support is the number of independent edits inferred to occur along the branch entering a node; panels show the percentage of nodes with support ≥1 (left) or ≥2 (right). Support is highest at the first division, dips sharply through the recording quiescence that follows, recovers at the E6.5 bin as further integrations acquire edits, and declines modestly toward the tips as TAPE exhaustion reduces the number of available marks. In both panels, points are plotted at bin midpoints, and bins containing no branch midpoint (panel **E**) or dated node (panel **F**) are omitted rather than plotted as zero (**E**: E1.5, E3.5; **F**: E2.5 in both blastomeres, E3.5 in B); panel **F** additionally begins at E1.5, the age of the blastomere subtree roots. These gaps, and the sparse sampling of neighboring early bins, reflect the small number of divisions inferred during the recording quiescence.

Two things follow. First, the ceiling is itself a tacit admission that the strict molecular clock is violated. Had edits accrued at a constant rate, the unconstrained fit would not have placed ~10^5^ lineages in an embryo then comprising a few hundred cells. Second, the shape of the correction tells us where the clock breaks down. Because dates moved substantially only for nodes originally placed before ~E3.5, the violation is concentrated in the interval between the 2-cell stage and gastrulation, with the ceiling substituting external biological knowledge (*i.e.* the number of cells the embryo contains each day) for a temporal signal the recorder apparently failed to supply. Joining the dated trees at a common root representing the unedited zygote yielded a single backbone phylogeny with 640,012 tips (Fig. 3A–C). We next asked how the dated tree looks from the perspective of the recorder itself, *i.e.* how the rate of editing varies over time.

A key feature of DNA Typewriter is that, at least in principle, its sequential design should stabilize the number of active recording sites, and thus the rate at which independent marks accumulate per unit time. In this embryo we wrote to, on average, ~55 of the 66 available sites by E13.5 across the 11 × 6-unit sequential TAPEs (Fig. 2C). To evaluate whether recording rate stabilization was achieved, we compared the observed accumulation against several idealized recording architectures, each tuned to reach the same mean of 55 edits by E13.5. A 1 × 66-unit writable substrate whose sites are edited independently and non-sequentially would require a per-site rate of ~0.13 edits/day, but its temporal resolution (*i.e.* the number of new marks written per unit time) would fall ~6-fold over the experiment, from ~8.8 edits per day at E0 to ~1.5 per day at E13.5, as the pool of unedited sites is progressively depleted (Fig. 3D, red line). A 1 × 66-unit sequential TAPE, by contrast, would accrue edits at a constant ~4.1 edits/day, holding temporal resolution flat (Fig. 3D, blue line). Finally, an idealized version of our actual architecture in embryo #3, *i.e.* 11 × 6-unit sequential TAPEs, would behave similarly to the 1 × 66-unit sequential TAPE scenario, except that the aggregate rate droops in the second half of development, as some 6-unit TAPEs reach their limit (Fig. 3D, green line).

What we actually observed in embryo #3 under constrained dating matched none of these idealized curves, but instead shows three phases (Fig. 3E): (i) a burst of DNA Typewriter activity at the 2-cell stage (~20 edits) that marks the founding blastomeres, potentially attributable to zygotic genome activation (ZGA); (ii) a near-complete cessation of editing through the subsequent cleavages and blastocyst formation; and (iii) a sharp resumption of DNA Typewriter activity around ~E6 that peaks at ~9 edits per cell per day (~0.75 per TAPE per day) and then declines to ~4 edits per cell per day as many of the TAPEs reach exhaustion. Consistent with this interpretation, a minimal model composed of three such phases (*i.e.* a 20-edit burst, no recording to E6.0, and a constant recording rate from E6.0 to E13.5, applied to 11 × 6-unit sequential TAPE and tuned to reach 55 edits by E13.5), broadly reproduces the observed editing rate dynamics (Fig. 3D, black line vs. Fig. 3E).

This three-phase dynamic has a direct corollary for the tree itself: an interval in which few marks are written is an interval in which few marks are available to resolve the divisions occurring within it. Recording dynamics therefore bear on topology as much as on timing. How reliable is the topology of this backbone tree? Node support measures like bootstrapping are a cornerstone of phylogenetics^55^, but their use in the lineage recording field is inconsistent, perhaps because cell phylogenies can be large enough to make resampling-based bootstrap computationally prohibitive. Even at the scale of the backbone subtree of either blastomere, a resampling bootstrap was indeed infeasible. We therefore devised an edit-count support statistic that, for each branch in the fixed topology, counts the number of independent sites whose most-parsimonious reconstructed edit history places a supporting edit on that branch (**Methods**). The resulting distribution was right-skewed, with a median of 3 supporting edits on the incoming branch in both A and B and a long tail extending beyond 20 supporting edits (fig. S3C); 78% (A) and 81% (B) of internal branches carried at least 1, and 64% (A) and 68% (B) at least 2, independent supporting edits.

Support varied systematically with developmental time, in a pattern that mirrors the recording dynamics described above (Fig. 3F). It was maximal at the first division, where the 2-cell burst deposits many independent edits and unambiguously resolves the A/B split; weakest for the divisions immediately following the burst, during the quiescent cleavage-stage window in which few marks are written; and then rose sharply as editing resumed and remained high through mid-organogenesis, before falling slightly toward the tips as accumulating TAPE exhaustion reduces the number of independent marks available to support the most recent splits (Fig. 3F). Notably, the low-confidence early window is precisely where unconstrained dating inflates the number of ancestral lineages beyond the number of cells the embryo then contains (fig. S3A), so that the lineage-count ceiling does the most work and the new dates shift most from the unconstrained estimates (fig. S3B). The branch-support profile, the editing-rate curve, and the magnitude of the dating correction thus localize the tree’s least-reliable region to the same early-cleavage window. However, node support recovers sharply as the embryo approaches gastrulation. By E6.5-E7.5, an interval in which the dated tree traces an expansion from 658 to 13,691 lineages (fig. S3A), DNA Typewriter activity has risen to 9.4 (A) and 8.8 (B) edits per cell per day (Fig. 3E), accompanied by a high level of node support that is maintained thereafter (Fig. 3F). The bulk of the reconstructed backbone phylogeny is therefore both well supported and reliably dated, with the caveat that the cleavages following the 2-cell stage are sparsely marked and therefore resolved and timed with much lower confidence.

### Cell-type-specific editing rates may further violate a strict molecular clock

The lineage-count ceiling corrects the conspicuous early-branching artifact, but the reconstruction violates a strict molecular clock^56^ in two ways already apparent above. First, editing is quiescent in the days following the first cleavage and then bursts back on around the onset of gastrulation. Second, DNA Typewriter’s theoretically constant recording rate is compromised as an increasing proportion of 6-unit TAPEs exhaust. However, a third concern, addressed by neither the ceiling nor the timing correction, is that prime editing rate may vary across cell types or states, *e.g.* through dependencies on proliferative state^57,58^ or differences in the levels of enzymes (*e.g.* PEmax, DNA repair factors) or small molecules (*e.g.* nucleotides) on which prime editing depends^41,59–61^.

To assess this in the context of this embryo, we calculated the mean number of post-burst edits per TAPE for each cell type (post-burst: edits accruing in the descendants of blastomeres A or B after the 2-cell stage burst), a metric consistent between blastomeres A and B (Spearman’s *r* = 0.80, *p* < 10^−29^). The 93 abundant cell types (>=0.01% at E13.5) fell within a remarkably tight 1.6-fold range in both blastomeres. However, the slowest-editing cell types were overwhelmingly hematopoietic (fig. S4A). As recording is cumulative and these cells presumably edited at typical progenitor rates before adopting a hematopoietic identity, this measure likely underestimates the true slowdown in the hematopoietic state itself. We considered two explanations: (i) reduced PEmax expression (*e.g.* due to hematopoietic lineage-specific silencing at one or more of its ~7 random integration sites) or (ii) reduced nucleotide supply to prime editing’s reverse transcriptase. Consistent with the first, PE transcript levels were significantly lower in hematopoietic cell types (A: p < 10^−5^; B: p < 10^−7^, Wilcoxon rank-sum test; fig. S4B). Consistent with the second, *Samhd1* expression was significantly higher in hematopoietic cell types (A: p < 10^−2^; B: p < 10^−2^, Wilcoxon rank-sum test; fig. S4C), in line with SAMHD1’s role in limiting the dNTP pool available to PEmax’s reverse transcriptase^59,60^. However, both factors are highly confounded with blood cell type identity, such that we cannot confidently attribute the lineage-specific slowdown to either, nor can we rule out that other blood-lineage-specific factors are at play. In fact, when non-blood cells are examined on their own, the correlations for both factors are flipped from expectation (*i.e.* higher editing rates in cell types with lower *PEmax* and higher *Samhd1* expression; fig. S4D–E).

Taken together, these observations show that timing *in vivo* cell lineage trees spanning development can be considerably more involved than assuming a strict molecular clock^56^. Our recalibrated dates should be read as reasonable approximations rather than exact ages, most reliable where independent cell-number estimates anchor them and where recording is active, and least reliable at the earliest divisions, toward the tips and in hematopoietic cell types. More faithful timing will require models that represent cell type- or cell state-specific rate dynamics directly, particularly as recording extends into later development, which brings both a greater diversity of cell types and a growing fraction of post-mitotic and slowly dividing cells.

### Expanding the E13.5 phylogeny to 1,340,794 cells by distance-based placement

Having built and dated a high-confidence backbone from cells for which ≥7 genotyped TAPEs were recovered, we returned to the second stage of reconstruction, *i.e.* distance-based placement of an additional 700,782 cells passing a lower recovery threshold (≥4 genotyped TAPEs). To do so, we simply attached each query cell to its single closest cell in the existing tree -- whether a backbone tip or a previously placed query cell -- under the same DNA Typewriter-specific edit distance used to build the backbone^48^. Query to anchor placements were restricted to whichever of the A or B subtrees the query cell had been assigned to by its founder signature, and cells were processed in order of decreasing TAPE recovery. This two-stage strategy rests on two assumptions: that the backbone samples the embryo densely enough that each query cell has a near relative among its tips, and that ≥4 genotyped TAPEs carry enough information to identify that relative. Neither is guaranteed, and the first is at best approximate, since the backbone represents only a fraction of the E13.5 embryo. We therefore assessed placement accuracy from two directions: the edit distances underlying each placement, and the cell type concordance of the nearest neighbors that placement creates.

From the perspective of edit distances, two features of placements are reassuring. First, their anchors are genuine close relatives rather than arbitrary attachment points. Query cells share a median of 10 (A) and 9 (B) sequential, post-burst edits with their anchor (fig. S5A). Second, most placements involve no anchor-query contradiction (A: 76%, B: 61%), and large numbers of contradictions are rare (fig. S5B). However, placements are also limited with respect to new topological information. Most query cells are genotype-identical to their anchor at every recovered site, with a mean of 0.6 (A) and 1.2 (B) private edits (*i.e.* edits carried by a query cell but not by its anchor) (fig. S5C). Accordingly, the divergence time implied by placement (*i.e.* the edit distance to the inferred common ancestor with the anchor, scaled by the clock rate) is low (median: 0 days for both; mean: 0.6 (A), 1.1 days (B); fig. S5D).

The second form of assessment -- cell type concordance of placements’ nearest neighbors -- is independent of the first. Placement used TAPE edit distances alone, with no reference to transcriptional state, so agreement between a placed cell and the cell-type annotations of its nearest tree neighbors is independent evidence that placement recovers genuine lineage relationships. Because placements join a query cell to a terminal tip or a previously placed query -- unlike the backbone, which was built by neighbor-joining -- inserting them reshapes the terminal topology of the tree. In the backbone alone, before placement, 67% of the 640,012 cells sat in cherries, 1% in polytomies, and 32% were loners. Inserting the 700,782 placements displaced many backbone cherry-partners into newly created subtrees, so that in the final tree only 48% of backbone cells remain in cherries, 5% sit in polytomies (*i.e.* where a query attached directly to a backbone tip), and 47% are now loners (*i.e.* their nearest co-terminal relative was displaced into a subtree by an inserted placement). The placed cells themselves are mostly resolved: 59% form cherries, 30% sit in polytomies, and 10% are loners (fig. S5E).

As a fair comparison in which every cell contributes a single value regardless of terminal context, we computed, for each cell, the fraction of its equally related nearest neighbors -- its cherry mate, or, for polytomy and loner cells, the leaves tied for the fewest intervening branches -- that shared its cell type, relative to a null obtained by permuting cell-type labels across tips (100 permutations; chance: 5.7%). In every terminal context, backbone-derived cells were somewhat more concordant than placement-derived cells, but both were far above chance: for cherries, 36% (backbone) vs. 30% (placed); for polytomies, 45% vs. 40%; and for loners, 34% vs. 24% (fig. S5F). Overall, backbone-derived cells matched a nearest neighbor’s cell type 35% of the time and placement-derived cells 32%, 6.2- and 5.7-fold above chance, respectively.

The results of these two assessments are independently reassuring. Placed cells sit at short edit distances from their anchors, and they recover cell-type concordance nearly as well as backbone cells do. The modest gap between them most likely reflects the lower recovery threshold used for placement than backbone construction (≥4 vs. ≥7 genotyped TAPEs, respectively), which leaves fewer marks with which to identify the correct relative. We conclude that placed cells are assigned to the correct local neighborhood, if not always to the correct depth -- a limitation that follows directly from the algorithm, in that query cells attach along terminal branches rather than internal edges in order to keep placement tractable.

### NextCell: an interactive explorer for large, dated, transcriptome-anchored phylogenies

**Figure 4.**
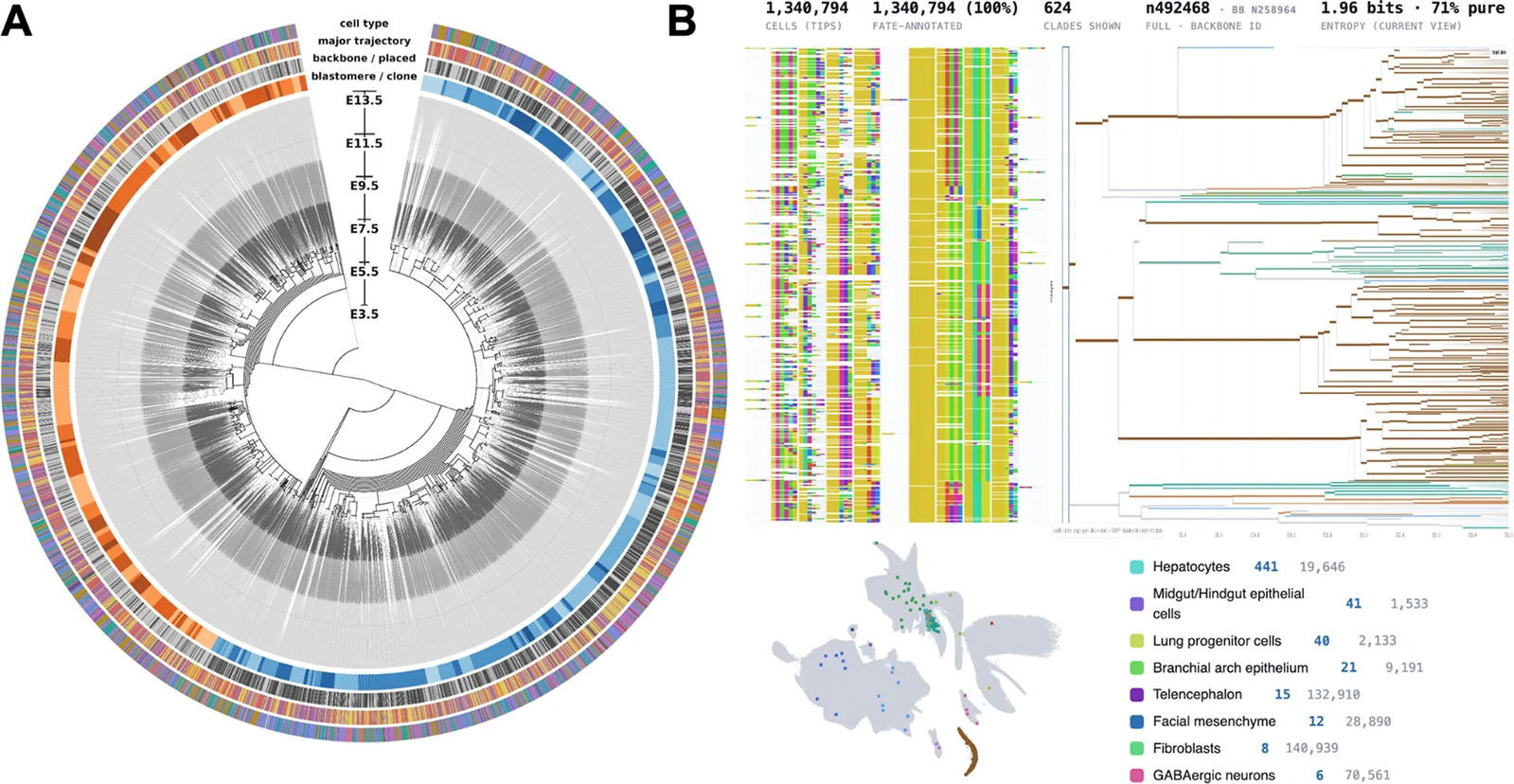
A ~10^6^ cell phylogeny of a developing mouse from zygote to late organogenesis, and an interactive browser to explore it. **(A)** 1,340,794 cells shown as a circular dendrogram in which radial position gives inferred node age, from zygote (center) to E13.5 (perimeter). Branch widths are scaled in 2-day steps to render the sparse early backbone legible. The two blastomere sub-lineages, A and B, occupy the right and left halves. Concentric rings encode, from inner to outer: (i) blastomere identity (A, blue; B, orange), with a distinct shade for each of the 182 ancestral lineages resolved at E6.0; (ii) reconstruction origin (backbone, dark grey; placement, light grey); (iii) major trajectory (legend in Fig. 2A); and (iv) cell type. **(B)** Interactive visualization in NextCell, focused on node n492468, an endodermally fated progenitor (E7.43) whose 624 sampled descendants are clonally partitioned among visceral organs, including liver (hepatocytes, n = 441), midgut/hindgut (n = 41), lung (n = 40) and branchial arch epithelium (n = 21). Panels show the edit states of 11 genotyped TAPEs (top left); a dendrogram of the subclade, with branches colored where ≥50% of descendant cells share a cell type (top right); a UMAP of all cells in the final tree, highlighting subclade members (bottom left); and cell types observed five or more times, with counts in the subclade and among all tips (bottom right).

The final zygote-to-late-organogenesis lineage of embryo #3 comprises 1,340,794 tips and 1,142,588 dated ancestral nodes (Fig. 4A). The tree is ultrametric, as all tips date to E13.5. Although these cells represent only ~10% of the E13.5 embryo, so few cells are present at early stages that the same tree provides, on average, 98-fold mean and 18-fold median “coverage” of the descendants of 13,691 inferred progenitors dated to E7.5. Each tip carries one of 25 major trajectory and 135 cell type annotations, and a single-cell transcriptome. The maximum polytomic fan-out is 168, but 97% of cells sit as singletons, in cherries, or in polytomies of ≤5 tips. To facilitate use by the community and inspired by NextStrain^62^, we built NextCell, an interactive explorer for this lineage. One can zoom in and out of clades and click any node to project its descendants onto the cell-state UMAP, while a synchronized strip displays the consensus TAPE edit pattern of every clade in view. Nodes can also be retrieved directly by identifier, and branches colored by germ layer, trajectory, cell type, or clade size, with a tunable purity threshold. For example, Fig. 4B shows node n492468, a progenitor dated to E7.43 whose descendants are endodermally fated, with subclades clonally partitioned among visceral organs. NextCell serves both the full 1,340,794-cell tree and the placement-free 640,012-cell backbone from which it was expanded, together with raw and intermediate files from each stage of processing.

### Two waves of clonal dominance, one as early as E6.0, and another during organogenesis

Clonal dominance, which we define here to mean the progeny of a few progenitors coming to outnumber those of the others, is a recurrent phenomenon in vertebrate development, typically studied in a tissue- or organ-specific manner^63–66^. In our early work with editing-based lineage tracing, we and colleagues documented extensive clonal dominance when sampling an entire organism. Specifically, in adult zebrafish, fewer than seven GESTALT alleles accounted for the majority of non-blood cells in each dissected organ^19^. There, however, editing tapered before gastrulation and was read out only in the adult, so dominance could be quantified but not dated. In the mouse, LoxCode barcoding of the E5.5 epiblast revealed clones that differ widely in both size and fate^67^, but a barcode induced once and read out at one endpoint cannot separate early restriction of a founder from the late expansion of its descendants.

**Figure 5.**
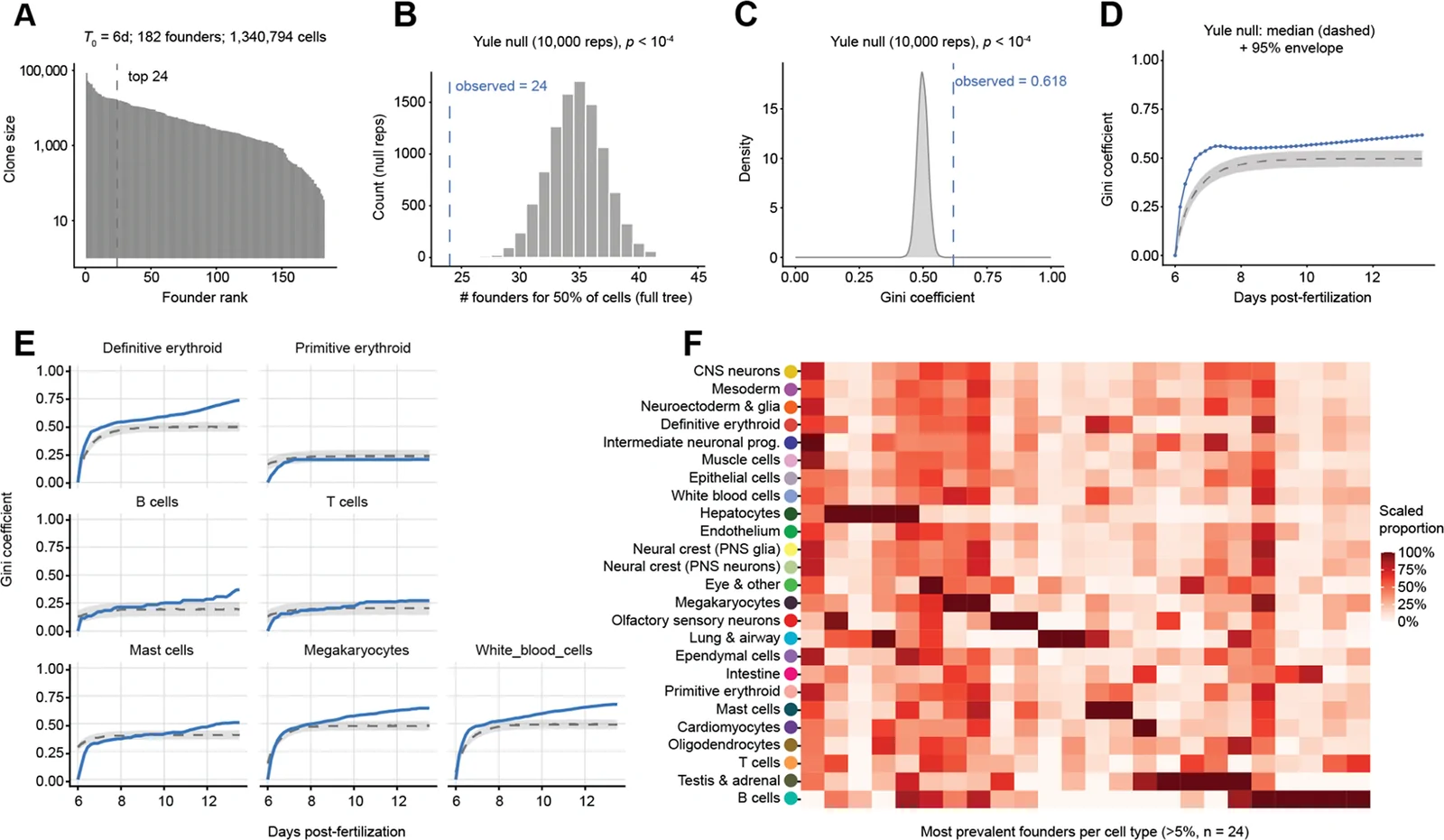
Two waves of clonal dominance, one as early as E6.0, and another during organogenesis. **(A)** Rank-abundance of founder clone sizes (log_10_ scale). Founder clones (n = 182) are defined as the lineages present at T₀ = 6.0 days post-fertilization in the dated tree; each founder’s clone size is its number of E13.5 descendants. The dashed line marks the top 24 founders. **(B)** Number of founders required to generate 50% of all E13.5 cells, in observed data (blue dashed line, n = 24) vs. the Yule null (grey, 10,000 replicates). *p* = *P* (null ≤ observed). **(C)** Gini coefficient of founder clone sizes (0 = every founder contributes equally, 1 = all cells derive from a single founder); the observed value (dashed line) is compared with its distribution under the Yule null (10,000 replicates). *p* = *P* (null Gini ≥ observed). **(D)** Emergence of dominance. The Gini coefficient computed across the same 182 founders’ descendant-lineage counts at each time T, tracking the fixed founder set forward from T₀ to E13.5 (blue), and comparing against the Yule null (grey) and its 95% envelope (grey). **(E)** As in panel **D**, but here computed for each of 7 hematopoietic major trajectories from founders with ≥1 descendant of that type (of 25; others shown in fig. S6E). Note that the null is cell-number sensitive, so the relevant comparison is between a trajectory and its own computed null rather than between trajectories. **(F)** Heatmap showing the contribution of individual founder clones to each major trajectory. Columns represent the 24 founders that contribute >5% of cells to at least one trajectory, ordered by the trajectory in which they are most enriched (following the row order) and, within each trajectory, by their maximum contribution, producing a block-diagonal arrangement. Rows represent trajectories, ordered by their total E13.5 cell number. Color indicates each founder’s contribution to a trajectory, normalized within each column (*i.e.* per founder) such that the founder’s largest contribution to a trajectory is set to 100% (that is, colors represent each founder’s relative contributions across trajectories, not its ranking against other founders within a trajectory). The >5% inclusion threshold corresponds to ~3 cells in the smallest trajectory (B cells; 66 cells) but ~18,800 cells in the largest (CNS neurons; 375,607 cells). As such, apparent founder biases in small trajectories should be interpreted with caution.

Here we sought to quantify clonal dominance throughout the mouse embryo and across the interval from gastrulation through late organogenesis. For this, we defined founder clones as the 182 ancestral lineages that date to E6.0 (Fig. 5A), using the dated backbone tree for temporal analyses and the full tree for topological ones. Although these founder clones have, on average, 7,367 profiled descendants at E13.5, their output was highly unequal across multiple measures. For example, only 24 founders were needed to account for 50% of all E13.5 tips, versus the 35 expected under a neutral Yule (pure-birth) model in which every lineage divides independently at the same constant rate, with exponentially distributed waiting times and no cell loss^68^ (Fig. 5B). We also calculated the Gini coefficient, a widely used measure of inequality, *i.e.* with a value of 0 corresponding to an equal contribution by each founder clone, and a value of 1 to all cells deriving from a single founder. The observed Gini coefficient of 0.62 is a marked departure from the expected value of 0.50 set by the neutral Yule model (Fig. 5C). Despite differing numbers of E6.0 founder clones (A: 110, B: 72), both blastomeres exhibit clonal dominance when analyzed separately, and to a similar extent (Gini coefficient for A: 0.64, B: 0.57) (fig. S6A–C).

To resolve when this inequality is established, we tracked the Gini coefficient across developmental time (E6.0-E13.5) (Fig. 5D, fig. S6D). The coefficient rises steeply from zero at E6.0 -- necessarily so, as founders are defined as the lineages present at that timepoint -- and plateaus by ~E8.0. A rise of this shape is also expected under the neutral Yule model, but there it asymptotes at 0.50; the informative quantity is therefore the departure from the null rather than the rise itself. The observed trajectory exceeds the null’s 95% envelope from ~E6.3 onwards, within one to two cell cycles of the founder timepoint and roughly coincident with the appearance of the primitive streak^69^, and remains above it throughout (*p* < 10^−4^). We conclude that the first wave of dominance is either already underway at E6.0 or begins within one or two divisions of it.

What might drive it? The Yule null assigns every lineage the same division rate, and each waiting time draw is independent, such that there is no possibility of a durable advantage. However, inequality in excess of that null can arise from division rates that differ between lineages and persist within them. Both conditions are plausible in this particular window. Cell cycles around gastrulation are the shortest of the mammalian life cycle and markedly heterogeneous, reported to range from 3 to 7.5 hours in the gastrulating rat^70^, with the fastest in the epiblast’s proliferative zone^54^. Remarkably, Snow’s speculation in 1977 that the proliferative zone’s descendants could quickly grow from 10% to 50% of the embryo if its rapid cell cycles were maintained until E7.5, may not have been far off the mark, as the 10% most proliferative founder clones (which we speculate to correspond to the proliferative zone) account for 39% of lineages dating to E7.5^54^. A second potential explanation, which is not mutually exclusive, is that founders differ in how many of their descendants survive. Either way, the inequality we observe at E13.5 is much smaller than differences of this magnitude would produce if fully inherited, which suggests that the plateau marks not the end of heterogeneity but the end of its heritability, after which growth is proportional and preserves the inequality already accrued.

Global inequality does not remain fixed after the plateau, but instead resumes a slow and steady rise through E13.5, and this second wave is cell-type dependent (Fig. 5E; fig. S6E–F). A full account of each of the 25 major trajectories is beyond our scope here, so we focus on hematopoiesis, where the distinct developmental origins of the successive blood waves offer a natural test of whether the timing and magnitude of dominance track tissue origin (Fig. 5E). Among the hematopoietic major trajectories, primitive erythroid is the exception, as its Gini coefficient remains within the null envelope across the entire window. This is consistent with the biology of yolk-sac primitive erythropoiesis, in which progenitors emerge as a single synchronous cohort, undergo a tight, non-self-renewing expansion, exhaust the progenitor pool by ~E9^71^, and thereafter mature semi-synchronously in the bloodstream^72^. The definitive lineages, by contrast, all diverge from the null as development proceeds. Definitive erythroid departs earliest and furthest, exceeding its null envelope by ~E8 and reaching a Gini coefficient of ~0.75 by E13.5, with white blood cells following a similar trajectory; both steepen after ~E10, coincident with the onset of fetal liver hematopoiesis (with the caveat that although the clonal aspect of this analysis should be robust to it, the hematopoietic slowdown in DNA Typewriter discussed above (Fig. S4) may bias dating within these lineages). Megakaryocytes and mast cells are intermediate, while lymphoid lineages exhibit similar trends but are still rare in these data. A straightforward interpretation is that definitive progenitors from later waves of erythropoiesis (*i.e.* EMP, HSPC)^73^ self-renew and compete for expansion over successive divisions, so small proliferative advantages accumulate. In contrast, primitive erythroid progenitors expand once, in lockstep, and afford no such opportunity for one clone to outcompete another. The ordering of these trajectories thus tracks the known succession of at least two waves of mouse hematopoiesis^74^, and suggests that the magnitude of organogenesis-phase dominance may relate to a lineage’s mode of expansion.

Finally, we asked whether dominant founder clones supply many cell types or few, and whether the supply of any given cell type is itself dominated. The largest of the 182 founder clones accounts for 6.3% of the embryo, including at least one cell from every major trajectory. Twenty-four founders each account for >5% of at least one of the 25 major trajectories, but these are overwhelmingly multipotent rather than confined to a specific germ layer or lineage (Fig. 5F; fig. S6G). Trajectories nonetheless differ in how concentrated their supply is. Lung & airway, as well as olfactory sensory neurons, were supplied by the fewest founders (n = 12 to reach 50%) and mesoderm and muscle cells by the most (n = 23 to reach 50%). The most extreme single contribution to a non-rare trajectory is one founder accounting for 9.8% of E13.5 megakaryocytes, but this is only a 1.6-fold enrichment over expectation, as it is also the largest founder clone. Conversely, a handful of founders are modest in size yet highly specific, *e.g.* two account for only 2.0% and 3.9% of all hepatocytes sampled at E13.5, yet their own descendants are 44% and 27% hepatocytes. Thus, at the founder scale, dominance and fate are largely decoupled. With few exceptions, the clones that expand most do so across the embryo rather than within a lineage.

### Tree siblings share fate far in excess of chance, and most strongly in recently expanding lineages

Our analysis of clonal dominance begins to address how fate maps onto lineage, but the behavior of founders defined at E6.0 is dominated by the sheer scale of the expansion that follows, while the structure most informative about the fate relationships among cell types lies closer to the tips. In the ensuing four sections, we turn to the tree itself -- reconstructed without reference to the transcriptional states or cell type annotations of its tips -- to ask how strongly, and from when, lineage predicts fate, and working from the finest scale upward.

We began with tree siblings (*i.e.* cherries and terminal polytomies). For each of 25 major trajectories and 129 cell types observed in both the A and B subtrees, we asked how often observed tree siblings share that fate, relative to random expectation as defined by a tip-label-permuted null. The ratio of observed to permuted pairs (with a pseudocount to stabilize rare fates) defines each fate’s sibling enrichment. Estimates of the total number of cells at E13.5 vary considerably by method, from *~*13M (counting of dissociated nuclei^4^) to ~20M (extrapolating from the ~7 trillion nucleated cells of a ~70 kg adult human to the ~200 mg estimated weight of an E13.5 mouse embryo^75^) to ~61M (whole embryo genomic DNA quantitation^5^). Because our 1,340,794 cell lineage tree captures, at best, ~10% of these, most tree siblings are not true sisters but nearest sampled relatives, and any fate sharing we observe is a conservative lower bound on true sibling concordance.

Globally, 51% (65%) of tree-sibling pairs share a cell type (major trajectory), an 8.9-fold (3.4-fold) enrichment over the permuted null. Enrichment varied widely across fates but was highly reproducible between blastomeres A and B (fig. S7A–B; Spearman’s ρ = 0.94 for major trajectories, 0.93 for cell types), and widespread (82/129 cell types and 17/25 major trajectories exhibited significant, ≥2-fold enrichment in both blastomeres (**tables S3–4**). Certain trajectories exhibited extreme enrichment, including endoderm derivatives such as lung & airway (139-fold), intestine (61-fold), and hepatocytes (33-fold), hematopoietic lineages such as megakaryocytes (42-fold), definitive erythroid (42-fold), and white blood cells (22-fold), and various lineages arising from localized founder pools such as ependymal cells (61-fold), olfactory sensory neurons (56-fold), and neural crest-derived PNS neurons (19-fold). At the cell-type level, additional strong enrichments included liver sinusoidal endothelial cells (107-fold), otic sensory neurons (89-fold), otic epithelial cells (79-fold), Kupffer cells (78-fold), dorsal root ganglion neurons (69-fold), and *Tgfb2*+ myelinating Schwann cells (68-fold). Many of these lineages are understood to commit from a small, spatially restricted progenitor pool and then expand clonally within a defined anatomical niche, *e.g.* lung and gut progenitors bud from the ventral foregut/midgut endoderm around E9 and proliferate locally to build the visceral organs^76,77^; hematopoietic and liver-resident cells descend from small precursor pools that seed the fetal liver microenvironment around E11^74,78^; and otic and dorsal root ganglion neurons emerge from placodal or neural-crest founder pools around E9.5 and expand within their local ganglion^79,80^. In each case, the cells sampled at E13.5 descend from a small number of committed progenitors dated to only a few days earlier, well within the window that our tree resolves, such that tree siblings are drawn overwhelmingly from the same fate.

### Late, heterotypic tree siblings highlight terminal differentiation-associated cell divisions

While many tree siblings share fate, a minority pair a proliferative progenitor or regional state with a post-mitotic derivative -- each a candidate snapshot of a single differentiation-associated division. Because we sample at best ~10% of the >10^7^ cells of this embryo, such differentiation-associated divisions are expected to be a minority of all heterotypic tree siblings. To identify them, we enumerated 959,215 tree sibling pairs from the full tree as above, and then asked which of 27 post-mitotic cell types (**table S5**) are recurrently paired with a progenitor or regional state. Restricting to recent divisions (by requiring a shared ancestor dated to E12.5 or later and at most one discordant TAPE site between the pair), and furthermore counting each post-mitotic cell once (credited to its nearest qualifying sibling), yielded 90,737 post-mitotic cells over 653 progenitor-derivative pairings observed ≥3 times. Against a tip-label, within-blastomere permutation null, 34 pairings were significant at a 1% Benjamini–Hochberg FDR and ≥3-fold enriched (max 25-fold), altogether comprising 13,752 post-mitotic cells. Reassuringly, their enrichments were highly correlated when calculated independently on the A and B subtrees (Spearman ρ = 0.85, p < 10^−111^; fig. S7C).

**Figure 6.**
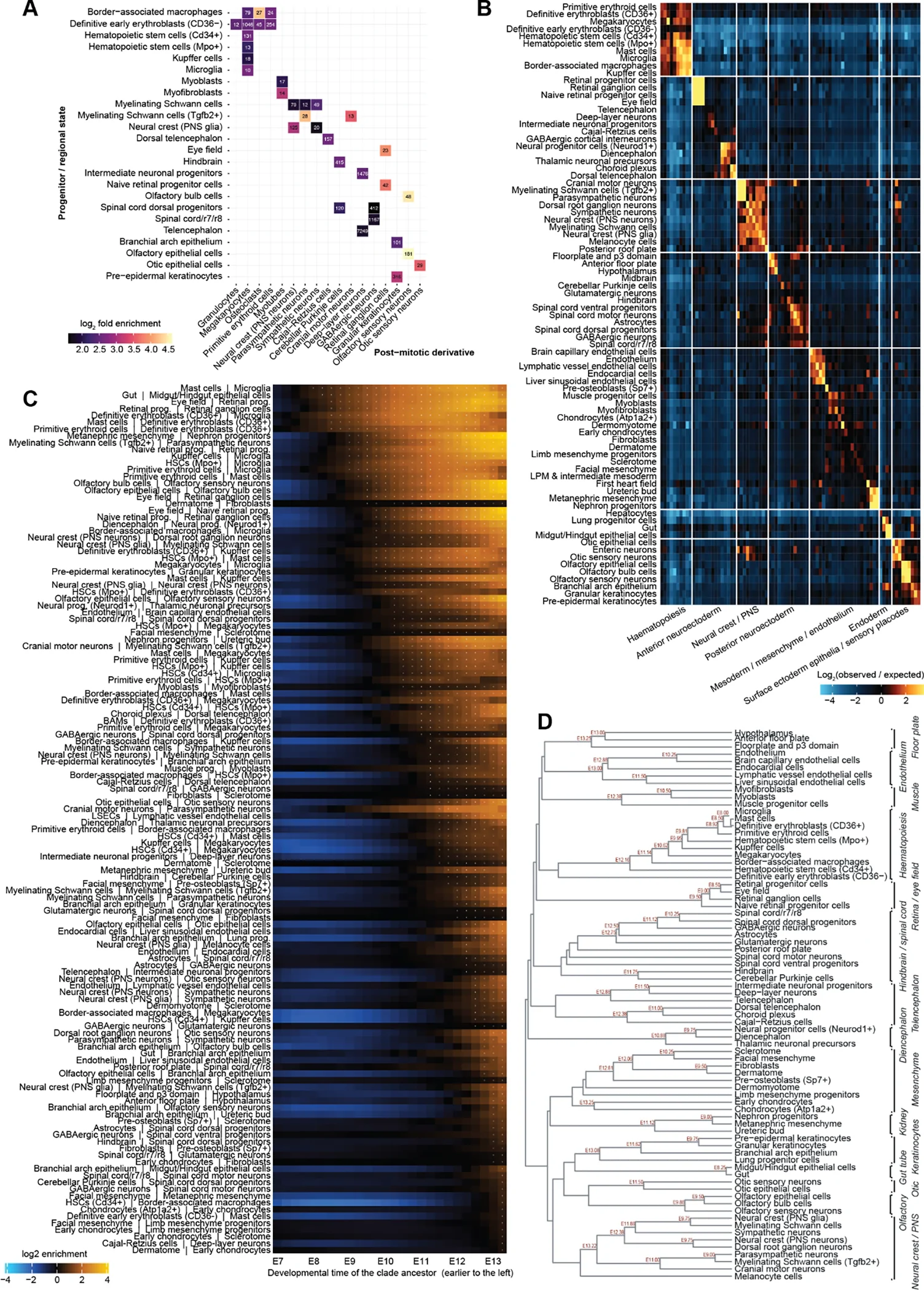
Lineage predicts fate at every scale, from sibling pairs to a dated hierarchy of cell-type couplings. **(A)** Heatmap of the 34 progenitor→post-mitotic heterotypic pairings that significantly and reliably recur among late-splitting tree siblings above chance. Rows, 24 progenitor or regional states; columns, 17 post-mitotic cell types; both ordered by germ layer. Color, log_2_-fold enrichment of the pairing over a tip-label-permuted null. Numbers within cells, post-mitotic cells supporting that pairing (range 10–7,249; 13,752 in total). White, pairing not captured. All pairings shown are supported by at least 3 post-mitotic cells, are at least 3-fold enriched, and clear a Benjamini-Hochberg FDR of 1% across the 653 pairings tested. **(B)** Briefly, we partitioned the tree into “maximal clades” (see text), and for each pair of abundant cell types, counted the number of clades containing both (including self-pairs) and compared this to the expected count under random cell-type label shuffling (100 permutations). Log_2_-enrichment (observed / expected), clipped to [−5, 3.5] is shown at K = 15, capturing local family groups. Cell types are ordered by hierarchical clustering (Ward’s linkage on Euclidean distance) of the K = 15 matrix with minor manual adjustments, and the same order is used across rows and columns. White lines were manually added and demarcate clusters of cell types corresponding to major groupings shown at bottom. **(C)** Timed fate couplings between cell types. Each row is one of the 128 cell-type pairs with a defined coupling depth. Each column is one of the 26 developmental time slices from E7.0 to E13.25 in 0.25-day steps, with earlier ancestors to the left. Color, log_2_-enrichment (observed / expected under tip-label permutation null) at that slice, averaged over timings calculated on each blastomere subtree independently. White dots mark slices at which the pair is co-enriched in both blastomere sub-trees independently. The leftmost white dot in each row therefore corresponds to a given pair’s coupling depth. Rows are ordered by coupling depth, deepest first, then by peak enrichment. Within each pair label, the cell type appearing earlier in the Qiu *et al.* developmental graph^5^ is listed first (a presentational guidepost and not a claim of directionality, which this analysis does not compute). Source values are provided in **table S6**. **(D)** A draft hierarchy of timed fate couplings. Average-linkage hierarchy over the matrix of pairwise coupling depths, built on 76 cell types that couple with at least one partner. The tree is drawn unscaled and cannot be read as a phylogeny; horizontal distance is merge rank, not time. Of its 75 branch points, the 51 that close at or before E13.25, the last time slice, are labelled with that coupling depth; the remaining 24 join only through pairs that never couple and so carry no date. As coupling depth reports how far back two fates remain confined to a shared pool of lineages, not when they diverged, groups closing earliest stay co-restricted deepest. Labels at the right of cell types name manually curated coherent groups. Six of 82 cell types couple with no partner at any slice by this method and are omitted: enteric neurons, first heart field, GABAergic cortical interneurons, hepatocytes, lateral plate and intermediate mesoderm, and midbrain.

The 34 divisions assign at least one progenitor to 17 of the 27 post-mitotic types, drawing on 24 distinct progenitor or regional states, and recover canonical differentiation events at the resolution of individual dated divisions (Fig. 6A). These included terminal differentiations associated with: (i) CNS neurogenesis (*e.g.* hindbrain and spinal cord dorsal progenitors → cerebellar Purkinje cells; intermediate neuronal progenitors → deep-layer neurons); (ii) PNS neurogenesis (*e.g.* neural crest glial progenitors → various peripheral neuronal subtypes); (iii) sensory neurogenesis (*e.g.* olfactory epithelium → olfactory sensory neurons; eye field and naive retinal progenitors → retinal ganglion cells; otic epithelium → otic sensory neurons); (iv) plausible germ layer boundaries crossings (*e.g.* olfactory bulb cells → olfactory sensory neurons; myelinating Schwann cells (Tgfb2^+^) → cranial motor neurons); (v) terminal maturation (*e.g.* pre-epidermal keratinocytes and branchial arch epithelium → granular keratinocytes; myofibroblasts and myoblasts → myotubes); and (vi) hematopoiesis (*e.g.* convergent megakaryocyte production from six distinct progenitors, as well as osteoclasts, primitive erythroid cells and granulocytes). Although plausible, the heterotypic blood pairings should be read with caution, as the slowdown in editing in hematopoietic lineages means that our thresholds for edit distance may not isolate recent divisions as well as for other lineages.

### Co-occurrence within maximal clades recovers recent and ancient lineage relationships among cell types

Tree siblings probe only the immediate neighborhood of each terminal node. To characterize “older” cell type relationships, we moved further up the tree, partitioning the A and B sublineages into maximal, terminal proximate clades^81^ and evaluating their enrichment for cell type pairs with clonal coupling metrics^26,82^. Specifically, for each internal node in the full tree, we identified “maximal clades” as subtrees containing ≥3 but no more than K cells, while its parent’s subtree exceeded K (*i.e.* clades that could not be grown further without breaching the size cap). We ran this at two granularities: K = 15 (small, tightly related family groups; 86,544 (A) and 60,289 (B) clades; median ancestor age E11.4 (A) and E11.3 (B)) and K = 200 (large, loosely related family groups; 10,468 (A) and 7,174 (B) clades; median ancestor age E8.9 (A) and E8.8 (B)). For each pair of 82 cell types represented by ≥100 cells in each blastomere subtree, we counted the maximal clades in which both were observed and compared this to expectation under a tip-label-permuted null (100 permutations).

Hierarchical clustering of the resulting K = 15 matrix of pairwise enrichments revealed clear blocks corresponding to hematopoiesis, anterior neuroectoderm, posterior neuroectoderm, neural crest & its derivatives, mesoderm/mesenchyme/endothelium, endoderm, and surface ectoderm epithelia and sensory placodes (Fig. 6B). Assignment was near-perfectly concordant with germ layer, with 78 of 82 cell types falling in the block dominated by their own layer. The four exceptions (cranial motor neurons and posterior roof plate (both neuroectodermal) grouping with neural crest; enteric neurons (neural crest) and olfactory bulb cells (neuroectodermal) grouping with surface ectoderm epithelia) involve fates that are spatially interleaved with the block they join. Because the tree was built without reference to transcriptional state or cell type annotation, this recovery of lineage organization from clade co-occurrence alone is a further indication that the reconstructed topology reflects genuine lineage relationships. This view is reinforced by close agreement between the two independently reconstructed blastomeres (off-diagonal Spearman’s ρ = 0.79 at K = 15, p < 10^−15^; fig. S8A).

Block structure was much sharper at K = 15 (more recent clades; fig. S8A) than K = 200 (more ancient clades; fig. S8B). Of 3,321 cell-type pairs, 145 were significantly enriched in both blastomeres at K = 15 but only 13 at K = 200, and just 8 at both (fig. S8C). We noticed that the 137 pairs enriched only at K = 15 overwhelmingly correspond to post-gastrulation fate relationships, while the 8 pairs enriched at both values of K remain coupled even within clades whose common ancestor dates to ~E8.9, implying that the fate coupling of the latter pairs dates to gastrulation or earlier. Consistent with this interpretation, 5 of these 8 pairs derive from the early myeloid and erythroid compartment (microglia with mast cells, Kupffer cells and Mpo+ HSCs; mast cells with Kupffer cells and primitive erythroid cells), while the remaining 3 mark the retinal field (retinal progenitors with eye field and retinal ganglion cells) and gut tube (gut with midgut/hindgut epithelium). That a handful of couplings persist across a 13-fold change in the clade-size cap, while the great majority do not, suggested to us that the depth at which enrichments appear or disappear is itself informative, and that sweeping the tree while varying this parameter might enable the systematic dating of lineally related cell type pairs throughout gastrulation and organogenesis.

### A hierarchical succession of timed fate couplings spanning late gastrulation to late organogenesis

To this end, we set out to build a hierarchy of timed cell type fate couplings. Rather than sweeping the clade-size cap as above, we instead swept developmental time directly. For each time *t* from E7.0 to E13.25 in 0.25-day steps, we took every lineage present at *t* and defined its clade as all of its sampled descendants. The number of clades defined by such time slices rises from 3,612 at E7.0 to 302,595 at E11.0. Focusing on clades containing ≥3 cells, we computed the same pairwise co-occurrence enrichment as above, here against a tip-label permutation null of 1,000 shuffles per slice per A/B-defined half-embryo. We called a pair significantly co-enriched when its observed number of shared clades exceeded the permuted mean by ≥3 permutation standard deviations, on ≥5 clades, and in both blastomere sub-trees independently. For each qualifying cell-type pair, we recorded its coupling depth, *i.e.* the earliest value of *t* at which the two remained significantly co-enriched. Of note, the deepest slices are also the least sensitive, dominated by a tail of very large clades, which a tip-label permutation expects to contain nearly every cell type, leaving little contrast to detect. Consistent with this, no pair of E13.5 cell types was co-enriched before E8.0.

This strategy yielded a succession of coupling depths for 128 cell-type pairs spanning 76 of the 82 cell types, with the earliest dating to E8.0 (mast cells and microglia) and the latest to E13.25 (13 pairs) (**table S6**). Although we do not have space here for a full review of these pairings, the vast majority are biologically sensible, as are their timings (Fig. 6C). Furthermore, the coupling depth estimates were highly reproducible between the two independently reconstructed blastomeres (Spearman’s ρ = 0.94, p < 10^−53^; median discrepancy of 0.25 days or one grid step, with 83% within a half-day; fig. S8D).

We proceeded to construct a hierarchy of couplings by average linkage, such that every branch point carries a developmental date (Fig. 6D). The hierarchy appears inverted with respect to a splitting lineage through time, but this is because in this representation, branches are joined, rather than split, at their deepest coupling. Upon visual inspection, clusters of coupled cell types generally correspond to major germ layers or organs, and groups whose members are most deeply coupled “close” first, *e.g.* the hematopoietic compartment at E8.5–9.0, the retinal progenitors at E8.5–9.6, the gut tube at E8.75, kidney progenitors at E9.3, the olfactory epithelium and its neurons at E9.5–9.9, and epidermal keratinocytes at E10.0.

Taken together, these four analyses highlight how much can be read from a large, dated cell lineage of a single mouse embryo in which only the terminal states are known. Working from the tips upwards, we found: (i) sibling pairs share fate far in excess of chance, most strongly in lineages that expand from localized progenitor pools; (ii) heterotypic siblings resolve differentiation-associated divisions and assign progenitors to post-mitotic cell types; (iii) the co-occurrence of cell types within larger clades recovers germ layer organization; and (iv) sweeping developmental time converts those co-occurrence patterns into a dated hierarchy of cell-type couplings that agrees strongly with the canonical relationships of mouse development. That this hierarchy: (i) was recovered from clonal co-occurrence at a single timepoint in a single embryo; (ii) is based on a topology that was reconstructed without reference to any transcriptional state or cell type annotation; and (iii) that it reproduces independently in the two “half-embryo” subtrees, speaks to how much even a single embryo’s incomplete lineage can say about mouse development.

### Heuristic inference of the transcriptional states and cell type annotations of ancestral nodes

In previous work, we assembled a cell type transition graph of 262 nodes and 474 edges spanning mouse development from E0 to P0 by linking transcriptionally similar states^5^. Constructing that graph required manual review of 1,155 heuristically nominated candidate edges against the developmental literature, and even so, many relationships remained ambiguous. This ambiguity is intrinsic to transcriptional snapshots, as similarity between two cell states at adjacent timepoints is equally consistent with direct lineage descent, convergence to a shared transcriptional program, or no developmental relationship at all^83^.

**Figure 7.**
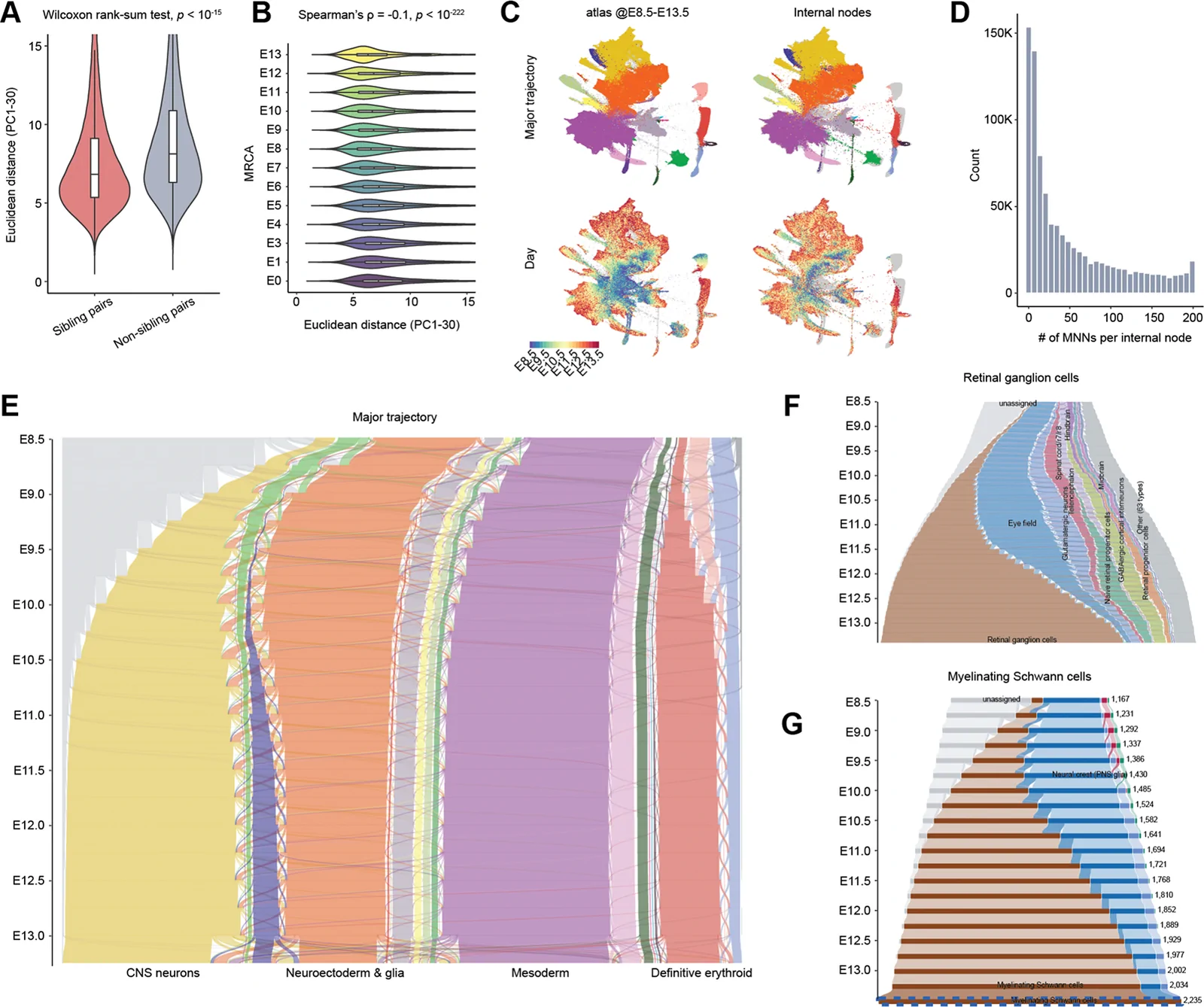
Heuristic inference of the transcriptional states and cell type annotations of ancestral nodes. **(A)** For each cell type, up to 10,000 sibling pairs were randomly sampled, along with a matched number of non-sibling control pairs of the same cell type (excluding known sibling and self-pairs). Transcriptional distance was defined as the Euclidean distance in the first 30 PCs. Violin plots show the distributions for sibling (red) and non-sibling (grey) pairs; inner box plots indicate the median and interquartile range. **(B)** Same-cell-type pairs, binned by most recent common ancestor (MRCA) depth on the lineage tree, were sampled at up to 10,000 pairs per unit-depth bin. Transcriptional distance was defined as the Euclidean distance in the first 30 principal components. Violin plots show the distributions grouped into MRCA-depth intervals; inner box plots indicate the median and interquartile range. Spearman correlation between transcriptional distance and MRCA unit-depth bin is reported. **(C)** 2D UMAP co-embedding of 1,943,035 single-cell profiles, comprising 896,782 internal nodes with imputed transcriptomes from embryo #3 (this study) and ~1 million cells from 21 timepoints of our high-temporal-resolution single-cell atlas of mouse development (E8.5–E13.5, 6-hour increments; each timepoint downsampled to 50,000 cells). UMAP was first computed on the atlas cells, and internal nodes were then projected onto this embedding. Both internal nodes and atlas cells were represented in a joint 100-dimensional PCA space, computed by integrating embryo #3 (all E13.5) with ~5.2 million single-cell profiles from the atlas (E8-E13.5); each internal node’s transcriptome was defined as the mean of the PCA coordinates of its assigned MNNs. The same embedding is shown split by dataset of origin (left: atlas; right: embryo #3), colored by major trajectory (top) and developmental day (bottom). **(D)** Histogram of the number of MNNs assigned to each of 896,782 internal nodes. **(E-G)** Examples illustrating NextCell’s ancestral state viewers. **(E)** All 1,340,794 E13.5 cells were traced back through the lineage tree and, at each time slice, assigned the inferred state of their nearest annotated ancestor; ancestral state calls are available to E8.5. Vertical position indicates developmental time. Total ribbon width represents all cells, and individual ribbon widths the fraction occupying each state; ribbon crossings indicate inferred cell-state transitions. Cells with no annotated ancestor are shown in grey (39% at E8.5). Single-slice A→B→A excursions (≤0.25 d) were removed. **(F)** Developmental origins of a terminal cell type, illustrated for the retinal ganglion cells. Traceback and time axis as in panel **E**, restricted to cells of the selected type at E13.5. Going backward in time, lineages coalesce into fewer ancestors. Bar width is proportional to the number of ancestral lineages, and segments are coloured by the inferred state of each ancestor (12 most abundant states shown individually, remainder grouped as "Other"). **(G)** Traceback view, shown for myelinating Schwann cells. Traceback, bar widths, segment colours, and ribbons as in panel **F**. Within each gap, one ribbon per state links ancestral lineages between adjacent time slices; same-state transitions are shown faintly and state changes as solid ribbons. Hovering over a ribbon displays the state transition and the corresponding number of lineages. The dashed bottom row represents the 2,235 E13.5 cells, with one lineage per cell and therefore a single state by construction.

We wondered whether the large, dated, tip-annotated phylogeny could supply what snapshots alone cannot. Encouragingly, examining the 1.34M-cell phylogeny at the granularity of transcriptional states rather than cell type labels, we find that within a given cell type, tree siblings are on average closer in transcriptional state (measured as Euclidean distance in the first 30 principle components (PCs)) than non-sibling cells of the same type (Fig. 7A), and that this similarity declines over the first few degrees of relatedness before saturating (Fig. 7B). These trends are nonetheless subtle, which is itself a measure of the difficulty, and a reason to lean on aggregation across many descendants rather than on individual siblings.

To impute a transcriptional state and cell type annotation for each of the 1,142,588 internal nodes of the phylogeny, we combined two resources: (i) the time-scaled full tree, in which every tip carries both a resolved lineage history and transcriptional profile; and (ii) our whole-embryo atlas of mouse development, which spans E8 to P0 at 2- to 6-hour intervals^5^. We deliberately chose a simple heuristic, both as a first test of feasibility and because it runs quickly even at this scale. We integrated the 1,753,895 single-cell profiles from embryo #3 (all E13.5) with 5.2M single-cell profiles from the atlas (E8–13.5) in a joint 100-dimensional PC space. Traversing the tree from tips to root, we assigned each internal node the mean PC profile of its immediate children. We then linked ancestral nodes to atlas cells by mutual nearest neighbors (MNNs) in successive 6-hour windows from E13.5 back to E8.5, requiring reciprocal membership among the 200 nearest neighbors in PC space. Each ancestor was then assigned an updated transcriptional profile mean PC profile of its MNNs (Fig. 7C) and a cell type annotation by majority vote among them (Fig. 7D); within this window, 17.5% of nodes had no qualifying MNN and were left unassigned, as were all 55,231 nodes predating E8.5, which fall outside the atlas interval entirely. Although the sibling-level signal is weak in individual cells, averaging over descendants recovers usable structure, as pseudobulk profiles aggregated by timepoint agree well between ancestral nodes and the atlas at the matched timepoint. Critically, unlike our previous graph^5^, in which cell type relationships were inferred from transcriptional similarity alone, here the lineage relationships are given by the tree, and the atlas serves only to identify the contemporaneous transcriptional state most consistent with each ancestral node.

This procedure assigned states to 896,782 of the 1,087,357 internal nodes dating to E8.5-E13.5, the interval covered by the atlas and to which we applied the heuristic (82.5%). Coverage was lowest at the earliest timepoints, with 39.8% of E8.5 ancestral nodes receiving a label, rising to 87% by E10.75 and 93–96% between E11.5 and E13.0, before falling to ~80% over the final half-day. Inferred states fall into three classes, two qualitatively distinct. 54% are “echoes”, where the inferred label was the single most frequent cell type among the node’s descendant tips and thus recapitulated information already present in the leaves; echoes were concentrated at late developmental stages (68% of E13.0 nodes) and in terminal cell types (definitive early erythroblasts 96%, hepatocytes 92%). Conversely, 20% of calls are “novel”, in that the inferred state appears in no descendant tip at all. These are genuinely inferred progenitor states, and include extraembryonic visceral endoderm, hematoendothelial progenitors, notochord, Tbx6+ mesodermal progenitors and placodal area (each ≥98% novel). The remaining 26% are intermediate, *i.e.* the label is present among the descendant tips but is not the most frequent. The echo and novel classes stratify cleanly by depth: at E8.5, 53% of calls are novel and 11% echoes, whereas by E13.0 this inverts to 10% novel and 68% echoes.

To be clear, the imputed cell type labels are very noisy. This is evident in the fact that even if we collapse “echoes” and ignore recursive patterns, there are 136,812 distinct cell type traversal patterns spanning 2+ states (range = 2 to 14 states, median 4) represented in the state-imputed version of the tree. These include, for example, 384 instances of [hematopoietic stem cells (Cd34+) → definitive early erythroblasts (CD36−) → megakaryocytes], which is biologically plausible, and a single instance of [sclerotome → lateral plate and intermediate mesoderm → otic epithelial cells → facial mesenchyme → definitive early erythroblasts (CD36−) → hematopoietic stem cells (Cd34+) → B cell progenitors], which is not.

Focusing on a single cell type, myelinating Schwann cells, there are 7,817 cells with this annotation among the E13.5 tips, of which 4,958 (63%) are associated with one of 1,723 routes. However, a single route, [neural crest (PNS glia) → myelinating Schwann cells], accounts for 1,488 of these cells (30% of those traced back), 15-fold more than the next most common, and it is strongly unidirectional in the tree (3,797 transitions in this direction vs. 316 in the reverse). The maturation intermediate is also recovered: [neural crest (PNS glia) → myelinating Schwann cells (Tgfb2+) → myelinating Schwann cells] (34 cells), in which both steps are curated edges and both unidirectional (asymmetry +0.61 and +0.61). By contrast, the next most frequent multi-step routes merely add a spurious state to the core transition, and in a consistently reversible pattern (asymmetry −0.01, +0.10 and −0.15).

Generalizing these criteria, we defined a path as qualifying if it is heterotypic and non-recursive, is carried by at least 1% of that cell type’s traced cells and at least 5 cells, and has every step directionally asymmetric in the tree (>=50 overall transitions, with at least a 60/40 preference for the observed direction). Importantly, all three criteria use only the phylogeny and the imputed labels, and no external knowledge of developmental biology. This heuristic yielded 251 qualifying paths spanning 77 of the 132 cell types with any traceable ancestry, collectively accounting for 121,055 cells (20% of those traced back) (**table S7**). Of these 251, 56 (22%) are multi-step, and 245 (98%) are recovered independently in both blastomeres.

Among the 251 qualifying paths are canonical transitions spanning every germ layer, including: extraembryonic visceral endoderm → hepatocytes^84^ (82% of traced hepatocytes); hematoendothelial progenitors → endothelium; muscle progenitor cells → myoblasts; dermomyotome → myoblasts; sclerotome → early chondrocytes and → chondrocytes (Atp1a2+); gut → each of lung progenitor cells, midgut/hindgut epithelium, foregut epithelium and pancreatic acinar cells; neural crest (PNS glia) → myelinating Schwann cells; placodal area → branchial arch epithelium; pre-epidermal keratinocytes → granular keratinocytes; eye field → naive retinal progenitor cells; and lateral plate and intermediate mesoderm → first heart field.

Multi-step routes are also recovered, including dermomyotome → muscle progenitor cells → myoblasts; hematopoietic stem cells (Cd34+) → border-associated macrophages → Kupffer cells; placodal area → pre-epidermal keratinocytes → granular keratinocytes; hematoendothelial progenitors → endothelium → liver sinusoidal endothelial cells; and lateral plate and intermediate mesoderm → posterior intermediate mesoderm → metanephric mesenchyme → nephron progenitors.

To evaluate these paths systematically rather than anecdotally, we scored every step against the aforementioned Qiu *et al.* (2024) graph^5^. Because our paths retain only nodes that received a call and collapse contiguous repeats, a single step may legitimately span more than one curated edge. Of the 311 testable steps in 251 qualifying paths, 141 (45%) connect states that are directly adjacent in the 452-edge curated graph and 199 (64%) connect states within two edges of one, against 3.1% and 8.4% expected for random pairs drawn from the same set of cell types (15-fold and 8-fold enrichment; p < 10^−6^ in both cases, based on label permutation). Of steps that are adjacent and orientable in the curated graph, 85 of 98 (87%) run in the curated direction, from the less to the more differentiated state.

In summary, combining a time-resolved atlas of mouse development with a resolved lineage history from a single mouse recovers a substantial share of the developmental relationships that took decades to establish, from essentially two whole-organism datasets. These results were obtained with no manual curation. The two datasets are complementary in precisely the way this problem requires. The atlas provides transcriptional states densely sampled across developmental time but carries no information about lineage, while the DNA Typewriter phylogeny provides the lineage relationships among 1.34 million cells of a single individual but is fixed at a single timepoint. Neither suffices alone, which is why our earlier graph, built from transcriptional similarity across timepoints, required manual review of >1,000 candidate relationships against the literature and still left many ambiguous. Here the lineage relationships are given, and the atlas is used only to identify the state most consistent with each ancestral node.

Imputed cell type annotations are painted directly onto the tree represented in the NextCell interactive browser, Qualifying paths and their temporal dynamics can also be interactively explored there, including as temporal flow-allocation diagrams inspired by Mittnenzweig et al.^9^ (Fig. 7E–G).

## DISCUSSION

Molecular technologies, like algorithms, have a theoretical scaling that is clean and a practical scaling that is not. At the outset of the genomic recording field around 2016, we and others imagined that the path from proof-of-concept to massive cellular phylogenies of vertebrate development would be a straightforward one -- a modest number of target sites, each drawing on a diverse vocabulary of marks, can in principle encode enough combinatorial diversity to label astronomical numbers of cells uniquely, as well as to relate them to one another. But the devil, of course, is in the details. The promise was frustrated less by any flaw in the idea than by a succession of practical obstacles, many specific to the slow, iterative reality of molecular bioengineering, and slower still *in vivo*. Roughly a decade on, this study confronts, in a single *in vivo* system, a number of the problems that have been rate-limiting for this paradigm, in service of an ambition present from the beginning, the reconstruction of the cell lineage of a large, opaque, regulatively developing organism, from zygote to adult.

It is worth returning to the eight obstacles laid out in the Introduction, and considering what progress, if any, is demonstrated here. Two are resolved by the design of DNA Typewriter itself^33,37^. Because it writes through prime editing rather than double-strand breaks, it largely avoids the deletion-driven erasure of neighboring sites that plagues nuclease-based recorders, at least within the context of individual TAPEs (**ii**, information loss). Because it writes strictly 5′→3′, temporal order is read directly from the genome rather than inferred (**iv**, event ordering), a feature that benefited the reconstruction, including the unambiguous marking of the first cleavage.

The remaining six we make progress on but do not fully resolve. PNI of two plasmids bearing DNA Typewriter’s three components, followed by random integration with piggyBac established a stable, constitutively expressed system through E13.5, but delivery was mosaic and integration number/locations uncontrolled, such that only 1 of 10 of evaluated embryos exhibited robust editing, and even that embryo showed early quiescence and a hematopoiesis-specific slowdown (**i**, delivery and expression). However, particularly if these limitations can be overcome, PNI may enable a quicker design-test loop than even HEK293T cells or organoids, while also letting us prototype directly *in vivo* (**viii,** cycle time). The sequential “typehead” of DNA Typewriter holds the number of active sites far more stable than consuming recorders can, though not indefinitely, as active sites per lineage fell from 11 (E0) to ~4.6 (E13.5) (**iii**, instability of temporal resolution). Inexpensive combinatorial-indexing profiling recovered >10^6^ cells, a substantial fraction of this E13.5 embryo but still orders of magnitude short of the number of cells in an adult mouse (**v**, completeness). circTAPE was a critical enabling advance for readout, but recovery per integration was uneven, as only ~50% of the full 1.75M cell × 11 TAPE matrix was confidently genotyped, and circTAPE recovery from nuclei fell well short of what circularization achieves in whole cells (**vi,** records retrieval). Finally, the neighbor-joining, sample-placement, and edit-count support strategies we bring to bear here enabled a time-calibrated phylogeny of 1,340,794 cells with parsimony-based node support, bringing trees of this scale within reach; we nonetheless favor an eventual transition to maximum likelihood methods that integrate our prior understanding of the molecular biology of sequential genome editing, accommodate missing data, retain node support, and scale to the direct incorporation of all informative cells into a fully dated tree^48,85^ (**vii**, tree building).

Three further obstacles were not on our list, yet loomed large in practice. The first is interpreting a dataset of this developmental complexity at all, *e.g.* annotating cell types across an entire mammalian embryo harvested during late-organogenesis was tractable here not because of the recorder but because of a parallel, multi-year effort to build a temporally resolved map of mouse cell states extending both well before and well beyond E13.5, against which our tips could be labeled^4–6^.

The second is that a tree whose internal nodes are unobserved is, on its own, close to uninterpretable. It reports which cell begat which, but not what any of those ancestors were. We make headway by leveraging clonal co-occurrence to build a dated hierarchy of cell-type couplings, and simple heuristics to infer the states and types of internal nodes and to trace cell types back through them, but these are first approximations. More broadly, the toolkit for so-called “developmental statistics”^86^ remains sparse relative to what these large phylogenetic objects can potentially support.

A third obstacle, also unanticipated, is comparative. Because we report a single embryo, our assessment of reproducibility rests on an internal control -- comparisons of blastomere A vs. blastomere B, the daughters of the first cleavage -- which holds genotype, staging, and dissociation constant but cannot speak to variation between animals. Integrating trees across individuals, genotypes, and stages remains a large and interesting problem, on which a wonderfully complementary recent preprint using PEtracer makes substantial headway with phylogenies ranging from ~1,000 to ~320,000 cells from sixteen E7.5-E10 embryos^35,87^.

Our delivery route deserves further comment, since it proved both the study’s sharpest limitation and an unexpected opportunity. When PNI worked it worked beyond our expectations, and the interval from injection to single-cell data was a few weeks. The editing pattern also permits a specific guess at what followed injection. Every integration is constitutional, indicating that all eleven circTAPE integrations and the PEmax cassette landed at the zygote stage. This is likely because piggyBac transposase mRNA was cleared with the maternal mRNA pool^88^, or because no unintegrated constructs remained available for integration after the first division. A large number of sequential edits distinguish the two blastomeres of the 2-cell stage. None of these edits are shared, and none appear in the cleavages that follow. Most, though not all, are “on vocabulary”, *i.e.* attributable to integrated epegRNAs. Together this implies a burst of PEmax and epegRNA expression from integrated copies at the 2-cell stage, coincident with major ZGA. The 2-cell stage has been found unusually permissive for genome editing in the mouse, both for large-fragment knock-in and for prime editing, although attributed there to repair and delivery dynamics rather than to transcriptional onset^89,90^. This burst is what unambiguously marks the daughters of the first zygotic cleavage, a resolution that previous prospective recorders have not achieved^21,22,27,67,91^, *e.g.* CARLIN and DARLIN induce editing at later stages, PEtracer and other donor-mESC approaches are rooted in donor cells rather than the zygote, and systems active from conception either resolved the first lineage segregations rather than the first cleavage, or did not extend beyond gastrulation.

Following the 2-cell stage burst, editing was suppressed for several divisions, resuming around E6 and remaining relatively stable thereafter, except in hematopoietic lineages, which became biased against editing as they differentiated. Interestingly, the PEtracer mouse preprint^87^ did not report an analogous blood-specific slowdown by E10, suggesting that the slowdown in embryo #3 may be due to positional effects on PEmax integrations, or to their sampling ending before such an effect would be apparent. The intervening quiescence is a harder feature to explain -- repair factor availability, dilution of episomes, maternal RNA clearance pathways^88^, delayed accumulation of PEmax and epegRNA from integrants after ZGA, and stage-specific activation or silencing of transgenes may all contribute, non-exclusively. Because both the early quiescence and blood-specific-slowdown of DNA Typewriter activity reproduced in both blastomeres against an identical set of integrations, concordance cannot separate features of mouse biology at these stages from the peculiarities of where our components integrated. Looking forward, DNA Typewriter mice generated through safe harbor integrations and crosses may distinguish the two. Profiling extraembryonic tissues, discarded here, may also help pin down the timing of the quiescent interval.

Several avenues for improvement are essentially arithmetic. Recording capacity is the product of TAPE number and TAPE length. The 11 × 6-monomer TAPEs used here afford 66 writable positions, whereas 50 × 12-monomer TAPEs would afford 600 -- enough, at the recording rates observed here, to record stably through and for several months past birth, which would better exploit the rate stability that sequential editing affords. We also note that better methods for dating 10^6^ to 10^8^-cell phylogenies, more stages and more cells, and the integration of signal-, state-, and spatially-resolved recording would each add a dimension the present tree lacks. As we have shown with ENGRAM^38^, the same TAPE substrate already supports symbolic recording of signaling and *cis*-regulatory activity. Furthermore, coupling to lineage-specific perturbation would let one ask how genetic lesions reshape developmental constraint and the timing of lineage restriction.

Massive, dense cellular phylogenies of development are a genuinely new kind of object, and they invite genuinely new kinds of analysis that remain in their infancy, *e.g.* clonal dominance, founder-pool structure, division motifs, and the statistical regularities that are invariant across individuals even when no single lineage is. It is these invariant features, we have argued elsewhere, that should define the resolution of a molecularly annotated “consensus ontogeny” -- a lineage-grounded reference onto which measurements and perturbations can be layered^92^. The tree reported here, one embryo at one stage comprising roughly 10% of its cells, is at best a single viewpoint on this consensus ontogeny. Yet it recovers, from clonal co-occurrence alone and without reference to transcriptional state, a dated hierarchy of cell-type relationships that agrees with one that we derived independently from a transcriptional time-series, but which there required extensive manual curation^5^. We have barely scratched the surface of what can be learned from even this one dataset, and we welcome its further exploration. To that end, all raw and processed data are freely available, alongside NextCell, an interactive browser for the annotated phylogeny.

Biology unfolds over time, within cells and tissues that are opaque to our eyes and instruments. Genomics is destructive and static; imaging is confined to a few channels in accessible systems. Recording offers a way out of this bind, writing each cell’s history into a medium that is faithfully copied across every division, such that a single destructive readout of an otherwise opaque organism recovers not its present state alone but the past that produced it. Sulston’s complete lineage, seen in this light, was possible only because his worm could be watched directly, division by division -- a luxury that opacity and scale deny us in nearly every other animal. Recording removes the requirement to watch at all. Following years of work across many groups to advance the underlying concept to its fullest potential^18^, we hope that this and contemporaneous studies^35,87^ may mark an inflection point for recording technologies. As more recording capacity, more stages, and the coupling of lineage to signal, state, and space are brought to bear, the outlines of a complete cell lineage of the mouse throughout its lifespan, and of the consensus ontogeny it would anchor, come into view.

## EXPERIMENTAL METHODS

### Molecular cloning

#### PEmax plasmid:

The chicken HS4 insulators, CAGGS promoter, and PEmax-P2A-dTomato-WPRE-bGHpA cassette were synthesized as gene fragments with compatible overhangs (GenScript). These fragments were excised using PaqCI restriction enzyme (NEB, R0745S) and assembled into a piggyBac transposon vector via Golden Gate assembly.

#### epegRNA-circTAPE plasmid library:

A partial segment of epegRNA, including the region encoding the partially degenerate insertion (NNNGGA), was synthesized as a library (IDT, Ultramer). The 6-unit circTAPE was synthesized as a DNA oligonucleotide (Ansa Biotechnologies). The remaining regions were synthesized as clonal DNAs (GenScript). These components were amplified by PCR and cloned into a piggyBac transposon vector by Gibson assembly using NEBuilder HiFi DNA Assembly (NEB, E2126S). Next, an oligo library for the TAPE barcode (NNNNNAANNNNN) was synthesized by IDT, amplified by PCR, and cloned into the epegRNA-circTAPE construct via Golden Gate assembly using NEBridge Ligase Master Mix (NEB, 1100S) and BsaI-HFv2 (NEB, R3733L). The sequences of synthetic oligonucleotides are listed in **File S1**.

### Animal husbandry

All animal experimentation was conducted according to the protocols approved by the Institutional Animal Care and Use Committee of the Allen Institute (Protocol #2530 and #2401). Mice were housed in a dedicated animal vivarium operating on a 14hr-10hr light-dark cycle and had access to food and water ad libitum.

### Zygote collections, pronuclear injection, and embryo transfer surgeries

Superovulation, zygote collection, pronuclear zygotic injection, and embryo transfer were performed as detailed in Manipulating the Mouse Embryo: A Laboratory Manual (4^th^ ed.; Behringer, Gertsenstein, Nagy, and Nagy, 2014). In brief, 4-week-old C57BL6/J females (The Jackson Laboratory, 000664) were superovulated via intraperitoneal injection of 5 I.U.s of human chorionic gonadotropin (hCG; Sigma-Aldrich, CG5) and 5 I.U.s of pregnant mare serum gonadotropin (PMSG; Ilex Life Sciences, A22725K). Superovulated females were then paired with fertile C57BL6/J males overnight to generate fertilized zygotes. E0.5 zygotes were collected from superovulated females the morning after mating. E0.5 zygotes were injected with a mixture of piggyBac transposon mRNA (GenScript, RP-A00064–0.2) and plasmid DNA in sterile, filtered IDTE (Integrated DNA Technologies, 11–01-02–02). Injected zygotes were transferred the same day into the ampulla of pseudopregnant CD-1 females (Charles River, 022).

### Harvesting and processing of E13.5 embryos

Once embryos reached the desired gestation stage, surrogate CD-1 females were euthanized and embryos were harvested, rinsed in 1x PBS, dabbed dried and immediately flash frozen in liquid nitrogen. Nuclei of E13.5 embryos were then isolated and fixed following the sci-RNA-seq3 protocol.

### Bulk amplicon-seq of TAPE

10% of the fixed nuclei from each embryo were subjected to genomic DNA extraction using Qiagen AllPrep DNA/RNA kit (Qiagen, 80204). Bulk amplicon-sequencing libraries spanning the TAPE region or the epegRNA-TAPE region were generated from the purified gDNA using two-step PCR and sequenced on a NextSeq 2000. Roughly 5 ng of purified gDNA was used for the first PCR with 0.4 μM of forward and reverse primers that included sequencing adaptors at their 5′ ends in a final reaction volume of 25 μl. In the first PCR, samples were incubated for 3 min at 95 °C; 15 s at 95 °C, 10 s at 65 °C and 45 s at 72 °C for 25–28 cycles; and 1 min at 72 °C. Products of the first PCR were purified using 1.0x AMPure beads (Beckman Coulter, A63882) and added to the second PCR that appended dual sample index sequences and flow cell adaptors. The second PCR used the same thermocycling program as the first, with the cycle number reduced to 6–8. Products were again purified using 1.0x AMPure beads and assessed on a TapeStation D1000 chip (Agilent, 5067–5582 Screentape and 5067–5583 reagent) before the sequencing run. Sequencing was performed on a NextSeq 2000 with a read structure of 318/10/10 (Read 1/Index 1/Index 2) or 70/10/200 (Read 1/Index 1/Read 2) for TAPE and epegRNA-TAPE libraries, respectively, using standard Illumina primers (TruSeq Read 1, Nextera Index 1, TruSeq Index 2). The sequences of synthetic oligonucleotides are listed in **File S1**.

### ddPCR on transgene copy number variation

To assess the number of PEmax integrations in the E13.5 embryos, 5 ng of purified gDNA was used as input for ddPCR. BioRad 2X ddPCR Supermix for Probes (BioRad 186–3023) was used according to the manufacturer’s protocol. Primers specific to a region of the Cas9 sequence were designed and validated. GAPDH ddPCR Copy Number Determination Assay (BioRad Assay ID dMmuCNS559838272) was used as the reference. The Cas9 probe was detected using FAM and the GAPDH probe was detected using Cy5.5. Assays were run on a QX600 AutoDG Digital Droplet PCR System (BioRad 17008371) and analyzed using QX Manager V2.2 Standard Edition software (BioRad). The sequences of the oligonucleotides, probes, and amplicons are listed in **File S1**.

### scRNA-seq (sci-RNA-seq3) of E13.5 embryos

Sci-RNA-seq3 was performed on fixed nuclei as previously described^41^ with the following differences: A new set of indexed reverse transcription primers and indexed ligation primers was used to widen the combinatorial space. (**File S2**). The nuclei were fixed in two steps, 1ml of resuspended nuclei was fixed first with 4ml methanol 15min on ice, and then rehydrated with Sucrose/Phosphate Buffered Saline/Triton X-100/MgCl2 (SPBSTM) buffer containing 2.5mg/ml BS3 (bis(sulfosuccinimidyl)suberate) (ThermoFisher #21580) for a second fixation for another 10min. In the first experiment, fixed nuclei (~24M of embryo#2, ~10M of embryo#3) were then split over 6 96-well plates for reverse transcription, incorporating the first of three indexes. The nuclei are then pooled and split over 6 fresh plates for ligation of the second index. They are pooled again, counted, and distributed to 23 new plates, aiming for 400,000 nuclei per plate. In a second, smaller, experiment, an additional 4M nuclei from embryo #3 were processed similarly, adding 2 more final plates for 25 plates total. These plates are then subjected to second strand synthesis, and the nuclei are disrupted with protease (Qiagen #19157). At this point, the freed cDNA is split - 1/3 will continue with the usual sci-RNAseq3 protocol and fragmented with Tn5 transposase (Diagenode #C01070010–100) loaded with Nextera N7 oligos, and then amplified with sci-RNAseq3 PCR primers. The remaining two-thirds undergo a separate amplification specific to the TAPE. First, an indexed primer (CAAGCAGAAGACGGCATACGAGAT##########GAAATAGGCCTGCATTAGTCTTGAGG), is used to perform 5 cycles of linear PCR at 60C annealing, with either KAPA Robust polymerase (Fisher #50–196-5219) or NEB OneTaq (#M0484L). After these linear cycles, the other primer is added for an additional 17 cycles of exponential PCR. Each plate of sci-RNA-seq3 PCR and TAPE PCR is pooled by plate, and size-selected by a dual-AMPure XP (Beckman Coulter #A63882) purification. The sci-RNA-seq3 and TAPE library pools are sequenced at a ratio of (~80% sci-RNA-seq3/~20% TAPE). Sequencing was performed on either Illumina NextSeq 2000, or NovaSeq X with custom primers. Read1 – 35 cycles, primer: Truseq_R1. Index1 – 10 cycles, primers: Nextera_idx1, tape_seqindex1. Index2 – 10 cycles, primer: Truseq_idx2. Read2 – 183 cycles, primers: Nextera_read2, tape_seqread2. The sequences of synthetic oligonucleotides are listed in **File S2**.

## COMPUTATIONAL METHODS

### Primary analysis of bulk TAPE

#### Input sequencing data.

Bulk TAPE amplicons were generated by PCR from embryo genomic DNA and sequenced for each embryo in which TAPE was detected, giving one amplicon FASTQ per embryo. Embryos #2 and #3 were single-end sequenced, and #6 was paired-end sequenced. For #6, the amplicon is carried on R2, so we reverse complemented it and then processed it identically to #1 and #2. Each amplicon read spans a 12-bp integration barcode, the six-monomer array, and the 3′ terminal landmark.

#### Integration barcode and insertion vocabulary derivation (tape/reference.py).

We defined two reference sets per embryo that are used for all downstream analysis (and, for embryo #3, reused for the single-cell data; see below). (i) A whitelist of TAPE integration barcodes (a 12-bp tag, NNNNNAANNNNN, that falls between GGCATG and CTCTGG flanks), derived from ~2M sampled reads as the 12N barcodes above ≥0.2% frequency and separated by ≥3 mismatches, to exclude single-mismatch "shadows" of abundant barcodes. (ii) An insertion vocabulary of monomer edit symbols. Because real edits are clonal and therefore concentrate at a specific integration and site, we counted each candidate insertion per (integration barcode × site position) across all reads. We admitted insertions that reached ≥10,000 reads at some single (barcode × position) combination to the insertion vocabulary of a given embryo.

#### Read parsing (tape/parser.py):

Each read’s integration barcode is extracted and corrected to the nearest whitelist entry within 2 mismatches (only when unambiguous). The 6-site monomer array is resolved both forward from the 5′ anchor (using the fact that editing is strictly ordered, so once a site is unedited all downstream sites are expected to be unedited) and backward from the 3′ terminal landmark (which recovers the distal sites of heavily edited molecules whose forward read-through might otherwise truncate). Reads with an edit following an unedited site (ordering violations) are discarded. A read is retained as valid only if its barcode is whitelisted, all six sites are resolved, and every edit matches the insertion vocabulary. Valid reads are tallied per integration barcode as counts over their 6-site genotype.

#### De-noising (tape/denoise.py):

Barcodes with <2,000 total valid reads were not analyzed. Within each retained integration barcode, we removed amplification and sequencing artifacts in four steps: (i) error-collapse, folding a rare genotype into a single-mismatch neighbor at least 2-fold more abundant; (ii) chimera removal, discarding fully-edited genotypes explained as the prefix of one abundant genotype joined to the suffix of another (partially-edited genotypes are exempt, since their unedited tails are shared legitimately); (iii) a minimum of 2 supporting reads; and (iv) a cross-barcode filter removing deeply-edited genotypes (≥3 edits) whose read support was highest in a different integration barcode, which flags integration-barcode swap recombinants.

#### Integration copy number (tape/copynumber.py):

piggyBac integrations are sometimes duplicated^93^. Because every barcode is sampled from the same cells at the same sequencing depth, a barcode’s total read abundance reports its genomic copy number, *i.e.* single-copy integrations share one abundance level, and barcodes carried as multiple genomic copies sit at integer multiples of it. We estimated the single-copy level as the median abundance of the single-copy barcodes and assigned each barcode an integer copy number; the total number of integrations is the sum of copy numbers, which exceeds the number of distinct valid barcodes when any are multi-copy, and per-cell genomic-equivalent counts were divided by copy number.

#### Cell-number estimation (tape/depth.py):

Because bulk data lack UMIs, we estimated genomic equivalents from read depth. For each integration in embryos #2 and #3, we took λ, the single-genomic-equivalent read depth, as the mode of the per-genotype read-count distribution in log space; the number of cells carrying a genotype was round(reads/λ), and the genomic equivalents per integration was their sum.

#### Per-integration lineage trees (tape/editchain.py + tape/tree.py):

For each integration in embryo #3, we built a prefix tree over the ordered edit symbols, in which every root-to-tip path traces a lineage’s editing history and tip depth equals the number of sites written. Reads representing 3′-truncated versions of a deeper genotype were attributed to that deeper lineage (folded onto the tree only when the deeper lineage was ≥4× more abundant, so that genuinely silenced shallow lineages are retained), yielding one lineage tree per integration barcode.

### Processing of sci-RNA-seq3 sequencing reads

For the sci-RNA-seq3 experiments, data were processed independently in batches of four PCR plates for read alignment and gene count matrix generation using our previously described sci-RNA-seq3 pipeline^4^ (https://github.com/bethmartin/sci-RNA-seq3_pipeline/tree/MEGAsci) with minor modifications to accommodate new RT and ligation oligos. The first experiment comprised 23 PCR plates divided into six processing batches, and the second experiment comprised two PCR plates processed as a single batch. Base calls were converted to FASTQ format using Illumina *bcl2fastq*/v2.20 and demultiplexed by PCR i5 and i7 barcodes using the maximum-likelihood demultiplexer *deML*^94^ with default settings. Downstream processing followed our sci-RNA-seq protocol.^42^ Briefly, PCR-indexed demultiplexed reads were further filtered on RT and ligation indices (ED < 2) and adapter-trimmed with *trim_galore*/v0.6.5 (https://github.com/FelixKrueger/TrimGalore) using default settings. Trimmed reads were aligned to the mouse reference genome (mm39) using *STAR*/v2.6.1d^95^ with default parameters and GENCODE vM37 gene annotations. Uniquely mapping reads were retained, and duplicates were collapsed using the combination of UMI sequence (ED < 2), RT index, ligation adaptor index, and read 2 end coordinate (*i.e.* reads sharing an RT index, ligation index, pcr index, tagmentation site, and UMI within ED < 2 were treated as duplicates). Mapped reads were then split into constituent cellular indices by the combination of all three indexes. Digital expression matrices were generated with the *HTseq* package^96^ (*python*/v2.7.13) by counting strand-specific UMIs mapping to exonic and intronic regions of each gene. Multi-mapping reads (*i.e.*, those assigned to multiple genes) were resolved by choosing the gene whose 3′ end was closest to the mapped position; reads mapping within 100 bp of the 3′ end of more than one gene were discarded. For downstream analyses, both expected-strand intronic and exonic UMIs were included in the per-gene single-cell expression matrix. Low-quality cells (UMI count < 100, genes detected < 100, or unmatched rate [proportion of reads not mapping to any exon or intron] ≥ 0.4) were filtered out.

### Doublet removal

To exhaustively identify and remove potential doublets, we applied a two-step procedure. First, we detected doublets directly with Scrublet. To reduce memory and runtime, the dataset was randomly partitioned into multiple subsets (six for most experiments), and *scrublet*/v0.1 pipeline^97^ was run on each subset with the following parameters: min_count = 3, min_cells = 3, vscore_percentile = 85, n_pc = 30, expected_doublet_rate = 0.06, sim_doublet_ratio = 2, n_neighbors = 30, and scaling_method = ‘log’. Cells with a doublet score above 0.2 were flagged as putative doublets. Second, we performed two rounds of clustering to identify subclusters enriched for these flagged cells, using *Scanpy*/v.1.6.0^98^. Gene counts on sex chromosomes and genes with zero counts across all cells were removed. Each cell was normalized by its total UMI count, the top 3,000 most variable genes were selected, and the resulting expression matrix was renormalized. Values were log-transformed (with a pseudocount) and scaled to zero mean and unit variance. Dimensionality was reduced by PCA (30 components), and a neighborhood graph was constructed with scanpy.pp.neighbors (n_neighbors = 50), followed by Louvain clustering (scanpy.tl.louvain, resolution = 1). Each resulting cluster was then subclustered using the same procedure at higher resolution (resolution = 3). Subclusters in which more than 15% of cells had been flagged by Scrublet were designated as doublet-derived. Cells were removed if they were either flagged individually by Scrublet or belonged to a doublet-derived subcluster.

The previous two steps were performed automatically, leaving some small clusters driven by doublets difficult to detect. We therefore applied a third step to further identify and remove doublets. It consists of a series of six substeps: (i) Each cell’s expression vector was reduced to retain only protein-coding genes, lincRNAs, and pseudogenes, with sex chromosomes excluded. (ii) Doublets identified in the previous two steps were removed. (iii) The key difference from the previous step is that here we detected cell partitions using the *partitionCells* function implemented in Monocle3^4^, which automatically partitions cells to learn disjoint or parallel trajectories based on concepts from approximate graph abstraction. (iv) Each cell partition was downsampled to 2,500 cells, differentially expressed genes across partitions were computed, and a gene set was assembled by combining the top 200 marker genes for each partition. (v) Cells from each main cell partition were subjected to dimensionality reduction using this set of top partition-specific gene markers. (vi) Subclusters that expressed low levels of the genes identified as differentially expressed in step 4, showed high expression of markers specific to a different partition, and had relatively high doublet scores were labeled as doublet-derived subclusters and removed from the analysis. Overall, 7–14% of cells were removed from each experiment through this procedure.

### Cell clustering and cell-type annotations

Following doublet removal, we applied additional quality filters to exclude: cells with more than 85% of reads mapping to exons; cells with log_2_ UMI counts greater than mean + 2 SD or less than mean − 1 SD; cells with a *Scrublet* doublet score > 0.1; cells with more than 5% of reads mapping to ribosomal genes (Ribo%); and cells with more than 5% of reads mapping to mitochondrial genes (Mito%). After filtering, 1,753,896 cells from the E13.5 embryo (embryo #3) were retained, with a median of 790 UMIs and 583 genes detected per cell. Rather than processing the new dataset in isolation, we integrated it with our recently published time-resolved scRNA-seq atlas of mouse development^5^, 11.4 million nuclei from 74 well-staged embryos sampled every 6 hours from E8 to P0, annotated with 36 major developmental trajectories and over 200 distinct cell types. For annotation transfer, we selected 7 timepoints spanning E12.75 to E14.25 from the atlas (1,645,424 cells) and combined them with the embryo #3 data. Because the atlas was originally aligned to mm10, we re-aligned the raw reads to mm39 and re-annotated genes using GENCODE vM37 to match the new dataset. The combined dataset was processed with *Scanpy*/v.1.6.0^98^ as follows: (i) genes were restricted to protein-coding, lincRNA, and pseudogene biotypes, and counts on sex chromosomes were removed; (ii) UMI counts were normalized to the total count per cell and log-transformed; (iii) the 2,500 most highly variable genes were selected, and their expression was scaled to zero mean and unit variance; (iv) PCA was performed, and the top 30 components were used to construct a neighborhood graph (n_neighbors = 50), followed by Leiden clustering (resolution = 1); (v) cells were visualized in 2D and 3D via UMAP (min_dist = 0.1). Within this shared PCA space (dims = 30), each new cell was assigned a major trajectory and cell-type label by majority vote among its 20 nearest neighbors in the atlas.

### Primary analysis of single-cell circTAPE

#### Input sequencing data:

Amplicons derived from circTAPE were obtained by RT-PCR amplification in the course of the sci-RNA-seq3 experiment on embryos #2 and #3. All single-cell circTAPE data analyzed here derive from embryo #3. These were subjected to single-end (NextSeq 2000 P3 200 platform, Read1: 35bp, Index1 10bp, Index2 10bp, Read2: 183bp) or paired-end (NextSeq 2000 P2 600 platform, Read1: 250bp, Index1 10bp, Index2 10bp, Read2: 250bp) sequencing. Reads were processed in the first part of sci-RNAseq3 pipeline to pull out the three indexes that would identify the cell to match it with its transcriptome. Each processed read header carries the cell identifier and an 8-bp unique molecular identifier (UMI).

#### Read merging (tape/merge.py):

In the paired-end data, R2 is the amplicon read (spanning the integration barcode through the array to the 3′ terminal landmark) and R1 is its mate from the 3′/poly-A end. R1 and R2 mates were merged into a single full-length consensus read by overlap using fastp^99^ (--merge --correction, with paired-end adapter detection; minimum overlap 15 bp, allowing ≤8 mismatches and ≤30% mismatched bases within the overlap), which corrects low-quality overlap bases using the higher-quality mate. Because the amplicon read (R2) alone spans the full barcode–array–terminal region at high quality while the majority of R1 mates were low-quality or uninformative, we used a merge-with-fallback scheme: where a pair merged (a minority of pairs, in which R1 is clean), the merged read was used; otherwise the amplicon read (R2) was used. Merged reads were reverse-complemented to the amplicon orientation. The single-end runs required no merging. Three runs contribute to the final callset: the paired-end run and one single-end run, which share a cell set and were pooled by (cell, UMI, integration); and a second single-end run covering a disjoint batch of cells (a different plate; zero cell-identifier overlap), which was called separately and whose cell × integration matrix was then row-concatenated with the first. The combined callset comprises 1,708,269 cells.

#### Reference sets, parsing, and de-noising.

The integration-barcode whitelist and insertion vocabulary for embryo #3 were taken directly from the bulk analysis above. Single-cell reads begin at the integration barcode (rather than after a GGCATG flank), so the barcode is taken as the 12-bp tag immediately 5′ of CTCTGG and corrected to the whitelist as in the bulk pipeline, and the six-site array is resolved and validated identically. Reads were tallied per cell, per integration, and per UMI. PCR duplicates were then collapsed to molecules by UMI. A UMI within one mismatch of a more-abundant UMI at the same cell and integration is merged into it, a merged UMI supported by fewer than 2 reads is then discarded (single-read UMIs are enriched for PCR and ambient error), and each molecule was assigned the dominant genotype among its reads. Within each (cell, integration), the per-barcode de-noising steps from the bulk pipeline (error-collapse of single-mismatch neighbors and prefix/suffix chimera removal) were applied to the molecule counts, and any genotype supported by fewer than 2 molecules was discarded. The cross-barcode swap filter is specific to the bulk population and is not applied per cell.

#### Per-cell consensus (tape/sc.py + tape/editchain.py).

A single consensus genotype was called per cell and integration using an edit-chain model. Because TAPE transcripts carrying earlier edit states may persist across cell divisions, a molecule with fewer edits that lies on the same ordered edit chain is a prefix (carry-over) of the cell’s current genotype rather than a distinct allele; the consensus therefore descends the most-supported edit chain, folding such prefix molecules onto it, and reports the deepest dominant genotype. Two different edit symbols competing at the same next site (which neither a single lineage nor carry-over can produce) define a branch; a locus is flagged as a putative doublet when any such off-path branch carries ≥2 molecules. Branching does not truncate the call (the consensus is always the deepest dominant tip) but it lowers the locus’s dominance, defined as the molecules on the dominant root-to-tip path (the called genotype’s own molecules plus every prefix molecule on that path) divided by all molecules at the locus, equivalently 1 − (branching molecules / all molecules). A locus is emitted only when the called lineage is supported by ≥3 molecules and its dominance is ≥0.9; otherwise it is left missing, on the principle that a wrong call harms the tree while a missing one does not. Together with the reads-per-UMI floor above, these thresholds retain ~98% of cells while removing ~27% of the lowest-confidence locus calls. This yields a cell × integration character matrix of consensus genotypes, together with per-cell quality metrics: the number of integrations recovered (n_loci), the number carrying a competing allele (n_doublet_loci), and the mean fraction of molecules supporting the called allele across loci (mean_dominance).

#### Blastomere routing (blastomere_route.py).

Before tree building, cells were partitioned into the two daughters of the first cleavage (A and B) from their founder edits directly, rather than letting the neighbor-joining distance resolve that split, which misassigns a few percent of low-coverage cells at the first division, either through shared missingness or an ambient bleed edit at a founder locus. Blastomere-defining alleles were taken at site 1 of each integration, the site at which the two founder chains diverge, by bipartitioning well-covered cells (≥8 recovered integrations, so that shared missingness could not drive the split) on the leading principal component of their rarity-weighted site-1 alleles, and retaining alleles present in ≥85% of recovered calls on one side and enriched by ≥0.30 over the other. This gave 20 defining alleles across all 11 integrations. Every cell was then routed by majority vote over these alleles, provided it carried at least one and no more than one allele of the opposite side; that single opposite-side call was treated as ambient bleed and set to missing. Cells with no founder allele, a tie, or opposite-side alleles at ≥2 integrations were set aside rather than assigned, and handed off rather than discarded, since their composition is not fate-neutral. Of 1,708,269 cells, 1,686,864 (98.7%) routed: 975,196 to A and 711,668 to B, and 21,405 (1.25%) were set aside. These per-cell metrics were then used for a final, non-destructive filter: cells were flagged as passing QC when they carried ≥4 recovered integrations, ≤1 doublet locus, and mean_dominance ≥0.95, retaining 1,340,943 of the 1,686,864 routed cells (79.5%). All routed cells remain in the released matrices; the distance step selects the passing set. The two blastomere matrices were then carried separately through distance, neighbor-joining, and dating.

### Reconstruction of the backbone tree

We reconstructed a time-calibrated phylogeny of ~1.3 million cells in three stages. First, we built a blastomere-resolved backbone tree from 640,012 cells with high-confidence TAPEs, using neighbor-joining on independently constructed distance matrices for each of the two founding blastomeres (A: 401,901 cells; B: 238,111 cells). Second, we applied molecular clock dating to each subtree to recover time-calibrated branch lengths. Third, we grafted the remaining ~700,782 cells onto this timed backbone using a fast sample-placement approach, avoiding full re-inference at this scale.

#### Cell selection and blastomere partitioning.

From the cells that passed in the upstream filtering procedure, we retained those with at least 7 observed tapes out of all 11, essentially taking a large set of the most informative cells for backbone reconstruction. Each retained cell was assigned to blastomere A or B according to whether its TAPE edits matched the blastomere-defining edits; a missing (unobserved) TAPE was treated as uninformative, not as a mismatch.

#### Distance matrix.

For each blastomere, we constructed a pairwise distance matrix from processed TAPE sequences, using a distance function adapted from Choi *et al*.^33^. Let $e_{i}(z)$ denote the number of edits that cell i acquired in tape z, and $p_{ij}(z)$ denote the length of the shared edit prefix between cells i and j in tape z. Writing $Z_{ij}$ for the set of tapes recovered in both cells i and j, we computed the distance as the average, over $Z_{ij}$, of the number of diverging edits:

$$D_{ij}=\frac{1}{\left|Z_{ij}\right|}\Sigma_{z\in Z_{ij}}\left[e_{i}(z)+e_{j}(z)-2\;p_{ij}(z)\right]$$

This distance metric essentially counts the number of diverging edits between two cells. We use the average over tape distances to handle missing TAPEs.

Per blastomere, we added one synthetic cell to the distance matrix that carries only the founder blastomere edits at each TAPE and otherwise unedited. This cell will later be used as an outgroup to root each subtree.

#### Neighbor-joining tree inference.

We built phylogenies independently for the two first division daughters (blastomeres A, B), using the 401,901 (A) and 238,111 (B) cells plus 1 synthetic outgroup cell per blastomere. For each blastomere, we inferred a neighbor joining tree with DecentTree’s RapidNJ^52^ , rooted the tree on its synthetic outgroup, and dropped that outgroup tip. Negative branch lengths, 6.5% (A) and 4.9% (B) of branches, were corrected with the BAT R package (v2.11.1) tree.zero()^50^, a Felsenstein–Kuhner correction that zeroes each negative edge while shortening its two child edges by the same total amount, approximately preserving tip-to-tip distances^51^.

#### Tree dating.

Rooted trees were time-scaled by least-squares dating in LSD2 (v2.4.4)^53^, fixing the root at day 1.5 (the two-cell blastomere founder) and all tips at E13.5, with variance mode −v 1, a 2-hour minimum branch length (0.083333 d), and near-zero input branches collapsed to polytomies at −l 0.01. Because a subsample genealogy cannot carry more coexisting lineages at any developmental time than the whole embryo had cells, we constrained dating with a literature-derived embryo cell-count ceiling (Supplementary Table 2), halved to reflect each blastomere’s expected share of the whole embryo. This per side ceiling was inverted into an order-statistic ladder of per-node minimum-age lower bounds such that the split raising the tree’s coexisting-lineage count to K cannot predate the first day the embryo held K cells. We supplied this ranking to LSD2 as a −d date file, with dating iterated over three passes that re-ranked nodes each time. The two dated per-side trees were finally joined onto a shared day-0 zygote root by a 1.5-day stem, producing a timed backbone tree of 640,012 tips.

### Phylogenetic placement onto backbone tree

Reconstructing a tree of ~1.3 million cells in one go lies beyond the reach of any current method for lineage recorder data, so we had to build it in pieces. One option is to first define early splits in the tree and then reconstruct the resulting subtrees independently. But the earliest divisions after the 2-cell stage had the sparsest editing and a cell sorted into the wrong subtree stays there, along with everything descended from it. So we opted for another strategy, where we first infer a backbone tree from a subset of high-quality cells and then place the remaining cells onto the backbone one at a time, scaling linearly.

Cells were added to the backbone tree in two stages. First, a *placement* stage assigned every unplaced cell to a best ancestor on the basis of shared edits, defining the topology of attachment. Second, a *grafting* stage converted each placement into a time-scaled branch on the dated backbone tree, preventing us from redating the entire tree.

To place a query cell, we identify an anchor among the tips of the backbone tree and any previously placed query cells. The anchor is the cell with the smallest edit distance to the query, in the same edit distance that built the backbone: the minimum number of edits separating two cells. Because this metric is coarse and frequently produces ties, we first favor candidates sharing the greatest number of tapes with the query and then those with the highest tape recovery.

Exhaustively comparing every query cell with every candidate would be computationally prohibitive. Instead, we use an inverted index of rare edits to retrieve a small set of likely candidates and compute the exact edit distance only for these, selecting the closest as the anchor. Because previously placed query cells are also eligible as anchors, closely related query cells naturally form local sublineages rather than attaching independently to the backbone. Query cells with identical genotypes are represented as a single polytomy rather than being arranged into an arbitrary ladder.

A placed cell needs an upstream branch as well as a position. We insert a new node between the anchor and the query, at the point implied by their shared edited prefix, the closest approximation to their real common ancestor. Because tape edits accumulate sequentially, each cell’s own distance to that ancestor is simply the edits it privately acquired past the shared prefix, and their sum defines the total distance between the query and the anchor. We convert the query’s edit distance to time by dividing it by the molecular-clock rate estimated for that blastomere. The new internal node is then placed at that interval before the sampling time, splitting the anchor branch as needed. If this would place the node older than the anchor’s parent, we instead cap it at the parent’s age. Both the anchor and query remain sampled at the observed time. Thus, placing a query rescales a single branch without altering the dates of the existing tree.

### Internal node support

Resampling-based bootstrap support is computationally infeasible at this scale, as rebuilding a 640,012-cell tree hundreds of times is prohibitively expensive. Instead, we report a per-branch edit-count support statistic that conditions on the inferred topology and measures how many independent editing events across tapes support each branch, rather than how often that branch recurs across bootstrap replicates. This statistic is conceptually related to the branch parsimony score used to assess support in large viral phylogenies, including UShER^100^, but addresses a different question. Whereas the UShER score quantifies confidence in the placement of a newly added sample, our statistic quantifies the support for a branch already present in a fixed tree.

Because edits are acquired irreversibly and in a fixed write order within each recording tape, the topology and tip genotypes uniquely determine the most parsimonious ancestral tape state at every internal node. If two descendant lineages differ at a site, that site and all downstream sites are inferred to be unedited in their common ancestor. Each edit is then counted once, on the branch where it first appears. Summing these counts across all 66 recording sites (6 sites on each of 11 independent tapes) gives a branch’s edit-count support: the number of independent loci whose reconstructed edit history supports that branch.

### Clonal dominance

#### Founder definition.

We cut the time-scaled tree at day T0=E6.0; each ancestral branch in the tree at that time defines one founder and its tip count at E13.5 is that founder’s clone size.

#### Dominance statistics.

We quantified skew in the founder clone size distribution in two ways: 1) the number of founders required to reach 50% of all cells in the embryo and (2) the Gini index of clone sizes across all founders computed with ineq∷Gini from the R package ineq (v0.2–13)^101,102^. The Gini index summarises inequality among founder contributions into one number (0 = all founders contribute equally, 1 = one founder produces all cells). To determine when clonal dominance emerged, we held the set of T0 founders fixed and recomputed the Gini index at 50 successive developmental time points by recounting the descendants of each founder present in the reconstructed cell phylogeny at each time point.

#### Null model.

Observed skew was compared to a neutral-drift null in which N0 founders (matching the T0 founder count) each undergo an independent Yule (pure-birth) process^68^ at a shared rate λ for the same duration ΔT (T0→E13.5). λ was fit so the null’s expected total population matched the observed cell count. The required founders for 50% statistic were tested via 10,000 replicate draws from this null; Gini was tested the same way. The null was refit independently for each blastomere and, for per-cell-type tests, resampled via multivariate hypergeometric draws to match each type’s observed cell count.

#### Tree basis.

Two tree versions were used depending on whether we performed a time informed or topological analysis: the full placement-augmented tree (1.34M tips) for the rank-abundance, founders-for-50%, and prevalent-founder-heatmap panels; the smaller backbone tree (640K tips) for the Gini-vs-null, Gini over time, and per-cell-type panels. Both are cut at the identical T0.

### Tree sibling analyses

Tree siblings were defined as pairs of tips sharing an immediate parent. This included: (i) cherries, in which the parent had exactly two tip children, and (ii) terminal polytomies, in which the parent had ≥3 tip children. Within each terminal sibling group all unordered pairs were enumerated, so a group of n tips contributes n(n−1)/2 pairs. Tips whose only siblings are internal nodes (372,161 of 1,340,794; 27.8%) contribute no pairs, to the observed data and to the null alike. Tree topology was extracted from the final placement tree (1,340,794 tips) using ape (v5.7.1), yielding 959,215 sibling pairs across 427,041 terminal sibling groups -- 361,162 cherries, and 65,879 polytomies (up to 168 tips) contributing 598,053 pairs -- with the A and B blastomere subtrees analyzed independently.

Enrichment was computed on the combined tree, with the independently reconstructed blastomere A and B subtrees analysed separately as replication. For each of the 25 major trajectories and the 129 annotated cell types present in both blastomere subtrees, we counted sibling pairs in which both cells shared the same annotation. To generate a null distribution, major-trajectory or cell-type labels were permuted across annotated tips with the tree held fixed (100 permutations), which preserves both the sibling structure and each label’s abundance. Sibling enrichment was calculated as (observed+1)/(mean permuted+1), with a pseudocount of 1 added to stabilize estimates for rare annotations. Reproducibility between blastomeres was assessed by Spearman correlation of enrichment values across fates.

#### Heterotypic sibling analyses.

Within the same sibling-pair set, we asked which progenitor→post-mitotic pairings recur among tree siblings above chance, taking each as a captured terminal differentiation division. Of the 135 cell types, 27 were classified as post-mitotic (**table S5**), and a sibling pair was heterotypic when exactly one of its members was post-mitotic. Analysis was restricted to pairs whose shared ancestor dated ≥E12.5 and whose members differed at no more than one discordant DNA-typewriter site among the sites called in both cells. Both criteria are independent of cell identity, so the eligible pair set is identical for observed and permuted labels. Each post-mitotic cell was counted at most once, credited to its nearest qualifying sibling (fewest discordant sites), so that a terminal polytomy cannot contribute more candidate divisions than it has cells. The null permuted cell-type labels across annotated tips within each blastomere with the tree held fixed, recomputing the whole procedure for each of 100 permutations. Fold enrichment was (observed+1)/(expected+1), p was the upper tail of a Poisson with mean (expected+1), and a pairing was called a captured division at Benjamini–Hochberg FDR<1% with fold ≥3, testing over the whole tree and treating the two blastomeres as validation rather than as a gate.

### Clade co-occurrence

Maximal clades were identified independently on the A and B blastomere subtrees of the full placed tree. For a given size cap K, we traversed each subtree and, at each internal node, evaluated the number of tips in its subtree counted over that blastomere’s leaves alone: if this count fell between 3 and K inclusive while the count for its parent’s subtree exceeded K, the node’s subtree was called a maximal clade. This yields a set of non-overlapping clades covering the largest possible groupings not exceeding K tips (terminal groups of fewer than 3 tips are not called, so the clades cover rather than exactly partition each subtree). Clades were identified separately at K = 15 and K = 200.

For each pair among the 82 cell types represented by ≥100 cells in both blastomere subtrees, we counted the number of maximal clades in which both cell types occurred (co-occurrence count), separately for each blastomere and each value of K. The null is tip-label permutation within each blastomere with the tree and clade partition held fixed. Enrichment for each pair was calculated as log2((observed+1)/(expected+1)), the pseudocount stabilising pairs with few co-occurrences. A pair was called significant when observed co-occurrence exceeded the null expectation by ≥3 Poisson standard deviations on at least 5 co-occurring clades; for the recent-versus-deep classification this was required in both blastomeres independently.

Hierarchical clustering of the K = 15 log2-enrichment matrix was performed using Ward linkage on correlation distance between matrix rows; the resulting cell-type order was applied to both rows and columns and to the K = 200 matrix for comparison. Reproducibility between blastomeres was assessed by Spearman correlation of off-diagonal log2-enrichment values.

### Timed fate couplings

For each time point from E7.0 to E13.25 in 0.25-day increments, we defined time-slice clades on the full placed and dated tree. Each branch intersecting time *t* defined one clade consisting of all sampled descendant tips. Time-slice clades were identified independently for the A and B blastomere subtrees as branches whose parent node predated *t* and whose child node was dated at or after *t*. Clades containing fewer than three cells of the blastomere under analysis were excluded.

For each of the 82 cell types analyzed previously, and for every cell-type pair, we counted the number of time-slice clades containing at least one cell of each type. A null distribution was generated by permuting cell-type labels across tips within each blastomere subtree (1,000 permutations per time slice), while holding tree topology and time-slice clades fixed. A cell-type pair was considered significantly co-enriched at a given time slice if its observed co-occurrence count exceeded the permuted mean by at least three permutation standard deviations, was supported by at least five clades, and met these criteria independently in both blastomere subtrees. Coupling depth was defined as the earliest time point at which a cell-type pair first satisfied these criteria. Reproducibility of coupling depths between blastomeres was assessed using Spearman correlation and the median absolute difference between blastomere-specific estimates.

#### Hierarchy construction.

A cell-type hierarchy was constructed from the matrix of pairwise coupling depths using average-linkage agglomerative clustering (scipy v1.11, scipy.cluster.hierarchy.linkage, method=‘average’), restricting the analysis to the 76 cell types with a defined coupling depth to at least one partner. Pairs that never couple were assigned a censoring value of E13.5, and dendrogram branch points were labelled with their average-linkage merge height, which is a coupling depth in embryonic days rather than an abstract merge distance. Branch points whose height exceeded the last time slice (E13.25) can only be carried by the censoring value and were left undated.

### Heuristic inference of the transcriptional states and cell type annotations of ancestral nodes

For ancestral state imputation, we repeated the joint preprocessing described above using the embryo #3 dataset together with 5.2 million atlas profiles spanning E8.0-E13.5^5^. The combined dataset was normalized, scaled, and subjected to principal component analysis in Scanpy (v1.6.0), retaining the first 100 principal components.

#### Ancestral state imputation.

Starting from the observed E13.5 tips, we traversed the full placement tree from tips to the root using a post-order traversal. Each internal node was assigned a principal-component profile equal to the unweighted mean of its children’s profiles, with tip nodes contributing their observed PC coordinates and internal nodes their previously computed mean profiles. Ancestral nodes were linked to atlas cells by mutual nearest neighbors (MNN), proceeding in successive 6-hour windows from E13.5 to E8.5. Within each window, an ancestral node and atlas cell were linked if each ranked among the other’s 200 nearest neighbors in the 100-dimensional PC space (Euclidean distance). Nodes without a MNN match were left unassigned (17.5%). Each matched node received a cell-type label by majority vote across its linked atlas neighbors. Each ancestor was then assigned an updated transcriptional profile mean PC profile of its MNNs.

#### Traceback path.

For each E13.5 tip we walked root-ward and recorded the imputed ancestral labels, skipping uncalled nodes and collapsing contiguous runs of the same label, giving one sequence of successive distinct states per lineage, read oldest to youngest and ending on the tip’s own observed cell type. Paths were required to be heterotypic (≥2 distinct states) and non-recursive (no state appearing twice). The latter removes lineages in which a state recurs after having been left, which reflects oscillation of the imputation between two co-resident labels rather than differentiation. Of 1,340,794 tips, 145,275 (10.8%) had no heterotypic ancestry and 603,291 (45.0%) were recurrent, leaving 592,228 traced on 136,812 distinct paths across 132 of the 135 cell types.

A path was additionally defined as qualifying if: (i) it was followed by at least 1% of that cell type’s traced cells and at least 5 cells, and (ii) every step was directionally asymmetric. Transitions were counted over nearest-called-ancestor pairs: for each node carrying a call whose nearest called ancestor carried a different call, one transition was recorded from the ancestor’s to the node’s label, with tips included. A step A→B was retained only if that pair had at least 50 transitions and asymmetry (n_AB − n_BA)/(n_AB + n_BA) ≥ 0.2, *i.e.* the observed direction favored at least 60:40. Both criteria use only the phylogeny and the imputed labels. This gave 251 qualifying paths in 77 cell types (121,055 cells; **table S7**). For each we recorded the number of independent origins, defined as distinct tree nodes at which its earliest state occurs, and whether it was recovered in both blastomere-derived halves of the embryo, defined by the two largest clades descending from the root.

#### Traceback evaluation.

Qualifying paths were scored against the curated cell-type relationship graph of Qiu *et al.*^5^, reconstructed from their Supplementary Tables 20 and 22; merging states that share a name and removing self-edges gives 262 states and 474 undirected edges. Our labels were mapped onto these states by normalised string matching with four manual assignments. The graph was used only to evaluate paths, never to select them.

For each step we computed the shortest-path distance d between its two states. d = 1 indicates direct adjacency; we also report d ≤ 2, which permits one intervening state, since paths retain only called nodes and collapse contiguous repeats and a single step may therefore legitimately span more than one curated edge. Both endpoints of all 311 steps in the 251 paths mapped into the graph. Significance was assessed by permuting the 95 cell-type labels appearing in the qualifying paths among themselves and recomputing every step distance (10^6^ permutations; empirical P = (1 + k)/(1 + N) for k permutations reaching the observed value); the observed values exceeded every draw. Directional agreement was assessed only for curated-adjacent steps: because the graph is undirected, orientation was taken from each state’s shortest-path depth from the “Oocyte” node, with a step counted as concordant if the ancestral state is strictly shallower; steps whose states lie at equal depth cannot be oriented and were excluded. Paths were additionally classified by whether all of their states share a germ-layer assignment.

## Supplementary Material

Supplement 1

## DATA, CODE, TREES, INTERACTIVE BROWSER

At the NextCell website, we host links to our GitHub repository, useful intermediate files (*e.g.* lineage trees, metadata, scRNA-seq matrices, etc), and raw data. The raw data are also available at GEO (GSE341627 (token: gbwpuyeqhrypxyt). All code used to generate the analyses and figures is available at https://github.com/seidels/dtt-mouse-analysis. The NextCell interactive browser was built with Claude Code.

## References

[1] Sulston J. E. & Horvitz H. R. Post-embryonic cell lineages of the nematode, Caenorhabditis elegans. Dev Biol 56, 110–156 (1977). 838129 10.1016/0012-1606(77)90158-0

[2] Sulston J. E., Schierenberg E., White J. G. & Thomson J. N. The embryonic cell lineage of the nematode Caenorhabditis elegans. Dev Biol 100, 64–119 (1983). 6684600 10.1016/0012-1606(83)90201-4

[3] Hatton I. A. The human cell count and size distribution. Proceedings of the National Academy of Sciences 120, e2303077120 (2023). 10.1073/pnas.2303077120 PMC10523466 37722043

[4] Cao J. The single-cell transcriptional landscape of mammalian organogenesis. Nature 566, 496–502 (2019). 30787437 10.1038/s41586-019-0969-x PMC6434952

[5] Qiu C. A single-cell time-lapse of mouse prenatal development from gastrula to birth. Nature 626, 1084–1093 (2024). 38355799 10.1038/s41586-024-07069-w PMC10901739

[6] Qiu C. Systematic reconstruction of cellular trajectories across mouse embryogenesis. Nat Genet 54, 328–341 (2022). 35288709 10.1038/s41588-022-01018-x PMC8920898

[7] Pijuan-Sala B. A single-cell molecular map of mouse gastrulation and early organogenesis. Nature 566, 490–495 (2019). 30787436 10.1038/s41586-019-0933-9 PMC6522369

[8] Qiu C. Evolutionary transfer learning enables organism-wide inference of mammalian enhancer landscapes. bioRxiv 2026.04.07.717039 (2026) doi:10.64898/2026.04.07.717039.

[9] Mittnenzweig M. A single-embryo, single-cell time-resolved model for mouse gastrulation. Cell 184, 2825–2842.e22 (2021). 33932341 10.1016/j.cell.2021.04.004 PMC8162424

[10] Salipante S. J. & Horwitz M. S. Phylogenetic fate mapping. Proc Natl Acad Sci U S A 103, 5448–5453 (2006). 16569691 10.1073/pnas.0601265103 PMC1414797

[11] Frumkin D., Wasserstrom A., Kaplan S., Feige U. & Shapiro E. Genomic variability within an organism exposes its cell lineage tree. PLoS Comput Biol 1, e50 (2005). 16261192 10.1371/journal.pcbi.0010050 PMC1274291

[12] Kim K.-M. & Shibata D. Tracing ancestry with methylation patterns: most crypts appear distantly related in normal adult human colon. BMC Gastroenterol 4, 8 (2004). 15059289 10.1186/1471-230X-4-8 PMC400737

[13] Walsh C. & Cepko C. L. Widespread dispersion of neuronal clones across functional regions of the cerebral cortex. Science 255, 434–440 (1992). 1734520 10.1126/science.1734520

[14] Chow K.-H. K. Imaging cell lineage with a synthetic digital recording system. Science 372, (2021). 10.1126/science.abb3099 33833095

[15] Farzadfard F. & Lu T. K. Synthetic biology. Genomically encoded analog memory with precise in vivo DNA writing in living cell populations. Science 346, 1256272 (2014). 25395541 10.1126/science.1256272 PMC4266475

[16] Livet J. Transgenic strategies for combinatorial expression of fluorescent proteins in the nervous system. Nature 450, 56–62 (2007). 17972876 10.1038/nature06293

[17] Pei W. Polylox barcoding reveals haematopoietic stem cell fates realized in vivo. Nature 548, 456–460 (2017). 28813413 10.1038/nature23653 PMC5905670

[18] Askary A. The lives of cells, recorded. Nat Rev Genet 26, 203–222 (2025). 39587306 10.1038/s41576-024-00788-w PMC12184736

[19] McKenna A. Whole-organism lineage tracing by combinatorial and cumulative genome editing. Science 353, aaf7907 (2016). 27229144 10.1126/science.aaf7907 PMC4967023

[20] Frieda K. L. Synthetic recording and in situ readout of lineage information in single cells. Nature 541, 107–111 (2017). 27869821 10.1038/nature20777 PMC6487260

[21] Kalhor R. Developmental barcoding of whole mouse via homing CRISPR. Science 361, (2018). 10.1126/science.aat9804 PMC6139672 30093604

[22] Chan M. M. Molecular recording of mammalian embryogenesis. Nature 570, 77–82 (2019). 31086336 10.1038/s41586-019-1184-5 PMC7229772

[23] Stadler T., Pybus O. G. & Stumpf M. P. H. Phylodynamics for cell biologists. Science 371, (2021). 10.1126/science.aah6266 33446527

[24] Raj B. Simultaneous single-cell profiling of lineages and cell types in the vertebrate brain. Nat Biotechnol 36, 442–450 (2018). 29608178 10.1038/nbt.4103 PMC5938111

[25] Alemany A., Florescu M., Baron C. S., Peterson-Maduro J. & van Oudenaarden A. Whole-organism clone tracing using single-cell sequencing. Nature 556, 108–112 (2018). 29590089 10.1038/nature25969

[26] Spanjaard B. Simultaneous lineage tracing and cell-type identification using CRISPR-Cas9-induced genetic scars. Nat Biotechnol 36, 469–473 (2018). 29644996 10.1038/nbt.4124 PMC5942543

[27] Bowling S. An Engineered CRISPR-Cas9 Mouse Line for Simultaneous Readout of Lineage Histories and Gene Expression Profiles in Single Cells. Cell 181, 1693–1694 (2020). 32589959 10.1016/j.cell.2020.06.018 PMC7530571

[28] Liu K. Mapping single-cell-resolution cell phylogeny reveals cell population dynamics during organ development. Nat Methods 18, 1506–1514 (2021). 34857936 10.1038/s41592-021-01325-x

[29] Chadly D. M. Reconstructing cell histories in space with image-readable base editor recording.bioRxiv 2024.01.03.573434 (2024) doi:10.1101/2024.01.03.573434.

[30] Winter E., Emiliani F., Cook A., Abderrahim A. & McKenna A. BASELINE: A CRISPR Base Editing Platform for Mammalian-Scale Single-Cell Lineage Tracing. bioRxiv 2025.03.19.644238 (2025) doi:10.1101/2025.03.19.644238. PMC13434336 42549577

[31] Chadly D. Regenerative base editing enables deep lineage recording. bioRxiv (2026) doi:10.64898/2026.02.06.703857.

[32] Anzalone A. V. Search-and-replace genome editing without double-strand breaks or donor DNA. Nature 576, 149–157 (2019). 31634902 10.1038/s41586-019-1711-4 PMC6907074

[33] Choi J. A time-resolved, multi-symbol molecular recorder via sequential genome editing. Nature 608, 98–107 (2022). 35794474 10.1038/s41586-022-04922-8 PMC9352581

[34] Loveless T. B. Open-ended molecular recording of sequential cellular events into DNA. Nat Chem Biol 21, 512–521 (2025). 39543397 10.1038/s41589-024-01764-5 PMC11952980

[35] Koblan L. W. High-resolution spatial mapping of cell state and lineage dynamics in vivo with PEtracer. Science 390, eadx3800 (2025). 40705858 10.1126/science.adx3800 PMC12766569

[36] CZI Cell Science Program CZ CELLxGENE Discover: a single-cell data platform for scalable exploration, analysis and modeling of aggregated data. Nucleic Acids Res 53, D886–D900 (2025). 39607691 10.1093/nar/gkae1142 PMC11701654

[37] Liao H., Choi J. & Shendure J. Molecular recording using DNA Typewriter. Nat Protoc 19, 2833–2862 (2024). 38844553 10.1038/s41596-024-01003-0

[38] Chen W. Symbolic recording of signalling and cis-regulatory element activity to DNA. Nature 632, 1073–1081 (2024). 39020177 10.1038/s41586-024-07706-4 PMC11357993

[39] Regalado S. G. Lineage recording in monoclonal gastruloids reveals heritable modes of early development. bioRxiv (2025) doi:10.1101/2025.05.23.655664.

[40] Martin B. K. Optimized single-nucleus transcriptional profiling by combinatorial indexing. Nat. Protoc. 18, 188–207 (2023). 36261634 10.1038/s41596-022-00752-0 PMC9839601

[41] Chen P. J. Enhanced prime editing systems by manipulating cellular determinants of editing outcomes. Cell 184, 5635–5652.e29 (2021). 34653350 10.1016/j.cell.2021.09.018 PMC8584034

[42] Cao J. Comprehensive single-cell transcriptional profiling of a multicellular organism. Science 357,661–667 (2017). 28818938 10.1126/science.aam8940 PMC5894354

[43] Lalanne J.-B. Multiplex profiling of developmental cis-regulatory elements with quantitative single-cell expression reporters. Nat Methods 21, 983–993 (2024). 38724692 10.1038/s41592-024-02260-3 PMC11166576

[44] Litke J. L. & Jaffrey S. R. Highly efficient expression of circular RNA aptamers in cells using autocatalytic transcripts. Nat Biotechnol 37, 667–675 (2019). 30962542 10.1038/s41587-019-0090-6 PMC6554452

[45] Li X. Chromatin context-dependent regulation and epigenetic manipulation of prime editing. Cell 187, 2411–2427.e25 (2024). 38608704 10.1016/j.cell.2024.03.020 PMC11088515

[46] Hurlbert S. H. The Nonconcept of Species Diversity: A Critique and Alternative Parameters. Ecology 52, 577–586 (1971). 28973811 10.2307/1934145

[47] Paul P. Comparison of Fluorometric and UV Spectrophotometric Findings for DNA Isolated From Formalin-Fixed Paraffin-Embedded Blocks, Fine Needle Aspiration Cytology Smears, and Blood. Cureus 13, e19583 (2021). 34926054 10.7759/cureus.19583 PMC8672151

[48] Seidel S. SciPhy: A Bayesian phylogenetic framework using sequential genetic lineage tracing data. Nat Commun (2026) doi:10.1038/s41467-026-73377-6. PMC13402801 42270637

[49] Saitou N. & Nei M. The neighbor-joining method: a new method for reconstructing phylogenetic trees. Mol Biol Evol 4, 406–425 (1987). 3447015 10.1093/oxfordjournals.molbev.a040454

[50] Cardoso P., Rigal F. & Carvalho J. C. BAT – Biodiversity Assessment Tools, an R package for the measurement and estimation of alpha and beta taxon, phylogenetic and functional diversity. Methods Ecol. Evol. 6, 232–236 (2015).

[51] Kuhner M. K. & Felsenstein J. A simulation comparison of phylogeny algorithms under equal and unequal evolutionary rates. Mol Biol Evol 11, 459–468 (1994). 8015439 10.1093/oxfordjournals.molbev.a040126

[52] Wang W. DecentTree: scalable Neighbour-Joining for the genomic era. Bioinformatics 39, (2023). 10.1093/bioinformatics/btad536 PMC10491953 37651445

[53] To T.-H., Jung M., Lycett S. & Gascuel O. Fast Dating Using Least-Squares Criteria and Algorithms. Syst Biol 65, 82–97 (2016). 26424727 10.1093/sysbio/syv068 PMC4678253

[54] Snow M. H. L. Gastrulation in the mouse : Growth and regionalization of the epiblast. Development 42, 293–303 (1977).

[55] Felsenstein J. CONFIDENCE LIMITS ON PHYLOGENIES: AN APPROACH USING THE BOOTSTRAP. Evolution 39, 783–791 (1985). 28561359 10.1111/j.1558-5646.1985.tb00420.x

[56] Bromham L. & Penny D. The modern molecular clock. Nat Rev Genet 4, 216–224 (2003). 12610526 10.1038/nrg1020

[57] Wang Q. Broadening the reach and investigating the potential of prime editors through fully viral gene-deleted adenoviral vector delivery. Nucleic Acids Res 49, 11986–12001 (2021). 34669958 10.1093/nar/gkab938 PMC8599732

[58] Petrova I. O. & Smirnikhina S. A. Prime Editing in Dividing and Quiescent Cells. Int J Mol Sci 26, (2025). 10.3390/ijms26083596 PMC12026808 40332080

[59] Ponnienselvan K. Addressing the dNTP bottleneck restricting prime editing activity. bioRxiv (2023) doi:10.1101/2023.10.21.563443.

[60] Liu P. Increasing intracellular dNTP levels improves prime editing efficiency. Nat Biotechnol 43, 539–544 (2025). 39322763 10.1038/s41587-024-02405-x PMC12092096

[61] Ferreira da Silva J. Prime editing efficiency and fidelity are enhanced in the absence of mismatch repair. Nat Commun 13, 760 (2022). 35140211 10.1038/s41467-022-28442-1 PMC8828784

[62] Andrews K. R. Nextstrain automates real-time phylodynamic analysis of open data for endemic and emerging pathogens. bioRxiv (2026) doi:10.64898/2026.03.23.713807.

[63] Kamimoto K. Heterogeneity and stochastic growth regulation of biliary epithelial cells dictate dynamic epithelial tissue remodeling. Elife 5, (2016). 10.7554/eLife.15034 PMC4951195 27431614

[64] Shakiba N. Cell competition during reprogramming gives rise to dominant clones. Science 364, (2019). 10.1126/science.aan0925 30898844

[65] Gupta V. & Poss K. D. Clonally dominant cardiomyocytes direct heart morphogenesis. Nature 484, 479–484 (2012). 22538609 10.1038/nature11045 PMC3340018

[66] Lopez-Garcia C., Klein A. M., Simons B. D. & Winton D. J. Intestinal stem cell replacement follows a pattern of neutral drift. Science 330, 822–825 (2010). 20929733 10.1126/science.1196236

[67] Weber T. S. LoxCode in vivo barcoding reveals epiblast clonal fate bias to fetal organs. Cell 188, 3882–3896.e19 (2025). 40378848 10.1016/j.cell.2025.04.026

[68] Website. https://www.jstor.org/stable/92117.

[69] Tam P. P. & Behringer R. R. Mouse gastrulation: the formation of a mammalian body plan. Mech Dev 68, 3–25 (1997). 9431800 10.1016/s0925-4773(97)00123-8

[70] Mac Auley A., Werb Z. & Mirkes P. E. Characterization of the unusually rapid cell cycles during rat gastrulation. Development 117, 873–883 (1993). 8325243 10.1242/dev.117.3.873

[71] Palis J., Malik J., McGrath K. E. & Kingsley P. D. Primitive erythropoiesis in the mammalian embryo. Int J Dev Biol 54, 1011–1018 (2010). 20711979 10.1387/ijdb.093056jp

[72] Kingsley P. D., Malik J., Fantauzzo K. A. & Palis J. Yolk sac-derived primitive erythroblasts enucleate during mammalian embryogenesis. Blood 104, 19–25 (2004). 15031208 10.1182/blood-2003-12-4162

[73] Palis J. Erythropoiesis in the mammalian embryo. Exp Hematol 136, 104283 (2024). 39048071 10.1016/j.exphem.2024.104283

[74] Medvinsky A. & Dzierzak E. Definitive hematopoiesis is autonomously initiated by the AGM region. Cell 86, 897–906 (1996). 8808625 10.1016/s0092-8674(00)80165-8

[75] Mu J., Slevin J. C., Qu D., McCormick S. & Adamson S. L. In vivo quantification of embryonic and placental growth during gestation in mice using micro-ultrasound. Reprod. Biol. Endocrinol. 6, 34 (2008). 18700008 10.1186/1477-7827-6-34 PMC2527569

[76] Zorn A. M. & Wells J. M. Vertebrate endoderm development and organ formation. Annu. Rev. Cell Dev. Biol. 25, 221–251 (2009). 19575677 10.1146/annurev.cellbio.042308.113344 PMC2861293

[77] Cardoso W. V. & Lü J. Regulation of early lung morphogenesis: questions, facts and controversies. Development 133, 1611–1624 (2006). 16613830 10.1242/dev.02310

[78] Ganuza M. Lifelong haematopoiesis is established by hundreds of precursors throughout mammalian ontogeny. Nat. Cell Biol. 19, 1153–1163 (2017). 28920953 10.1038/ncb3607 PMC5705075

[79] Bronner M. E. & LeDouarin N. M. Development and evolution of the neural crest: an overview. Dev. Biol. 366, 2–9 (2012). 22230617 10.1016/j.ydbio.2011.12.042 PMC3351559

[80] Streit A. The preplacodal region: an ectodermal domain with multipotential progenitors that contribute to sense organs and cranial sensory ganglia. Int. J. Dev. Biol. 51, 447–461 (2007). 17891708 10.1387/ijdb.072327as

[81] Balaban M., Moshiri N., Mai U., Jia X. & Mirarab S. TreeCluster: Clustering biological sequences using phylogenetic trees. PLoS One 14, e0221068 (2019). 31437182 10.1371/journal.pone.0221068 PMC6705769

[82] Weinreb C., Rodriguez-Fraticelli A., Camargo F. D. & Klein A. M. Lineage tracing on transcriptional landscapes links state to fate during differentiation. Science 367, (2020). 10.1126/science.aaw3381 PMC7608074 31974159

[83] Wagner D. E. & Klein A. M. Lineage tracing meets single-cell omics: opportunities and challenges. Nat Rev Genet 21, 410–427 (2020). 32235876 10.1038/s41576-020-0223-2 PMC7307462

[84] Kwon G. S., Viotti M. & Hadjantonakis A.-K. The endoderm of the mouse embryo arises by dynamic widespread intercalation of embryonic and extraembryonic lineages. Dev Cell 15, 509–520 (2008). 18854136 10.1016/j.devcel.2008.07.017 PMC2677989

[85] Pilarski J., Stadler T. & Seidel S. Assessing the inference of single-cell phylogenies and population dynamics from CRISPR lineage recordings. PLoS Comput Biol 22, e1014370 (2026). 42258486 10.1371/journal.pcbi.1014370 PMC13268653

[86] Farrell J. A. Single-cell reconstruction of developmental trajectories during zebrafish embryogenesis. Science (2018) doi:10.1126/science.aar3131. PMC6247916 29700225

[87] Colgan W. N. Comprehensive Lineage Tracing Maps the Landscape of Cell Fate Decisions in Mouse Embryogenesis. bioRxiv (2026) doi:10.64898/2026.05.07.722278.

[88] Hamatani T., Carter M. G., Sharov A. A. & Ko M. S. H. Dynamics of global gene expression changes during mouse preimplantation development. Dev Cell 6, 117–131 (2004). 14723852 10.1016/s1534-5807(03)00373-3

[89] Kim-Yip R. P. Efficient prime editing in two-cell mouse embryos using PEmbryo. Nat Biotechnol 42, 1822–1830 (2024). 38321114 10.1038/s41587-023-02106-x PMC11631759

[90] Gu B., Posfai E. & Rossant J. Efficient generation of targeted large insertions by microinjection into two-cell-stage mouse embryos. Nat Biotechnol 36, 632–637 (2018). 29889212 10.1038/nbt.4166

[91] Li L. A mouse model with high clonal barcode diversity for joint lineage, transcriptomic, and epigenomic profiling in single cells. Cell 186, 5183–5199.e22 (2023). 37852258 10.1016/j.cell.2023.09.019 PMC13409469

[92] Domcke S. & Shendure J. A reference cell tree will serve science better than a reference cell atlas. Cell 186, 1103–1114 (2023). 36931241 10.1016/j.cell.2023.02.016

[93] Wang W., Bradley A. & Huang Y. A piggyBac transposon-based genome-wide library of insertionally mutated Blm-deficient murine ES cells. Genome Res 19, 667–673 (2009). 19233961 10.1101/gr.085621.108 PMC2665785

[94] Renaud G., Stenzel U., Maricic T., Wiebe V. & Kelso J. deML: robust demultiplexing of Illumina sequences using a likelihood-based approach. Bioinformatics 31, 770–772 (2015). 25359895 10.1093/bioinformatics/btu719 PMC4341068

[95] Dobin A. STAR: ultrafast universal RNA-seq aligner. Bioinformatics 29, 15–21 (2013). 23104886 10.1093/bioinformatics/bts635 PMC3530905

[96] Anders S., Pyl P. T. & Huber W. HTSeq--a Python framework to work with high-throughput sequencing data. Bioinformatics 31, 166–169 (2015). 25260700 10.1093/bioinformatics/btu638 PMC4287950

[97] Wolock S. L., Lopez R. & Klein A. M. Scrublet: Computational Identification of Cell Doublets in Single-Cell Transcriptomic Data. Cell Syst 8, 281–291.e9 (2019). 30954476 10.1016/j.cels.2018.11.005 PMC6625319

[98] Wolf F. A., Angerer P. & Theis F. J. SCANPY: large-scale single-cell gene expression data analysis. Genome Biol. 19, 15 (2018). 29409532 10.1186/s13059-017-1382-0 PMC5802054

[99] Chen S., Zhou Y., Chen Y. & Gu J. fastp: an ultra-fast all-in-one FASTQ preprocessor. Bioinformatics 34, i884–i890 (2018). 30423086 10.1093/bioinformatics/bty560 PMC6129281

[100] Turakhia Y. Ultrafast Sample placement on Existing tRees (UShER) enables real-time phylogenetics for the SARS-CoV-2 pandemic. Nat. Genet. 53, 809–816 (2021). 33972780 10.1038/s41588-021-00862-7 PMC9248294

[101] Zeileis A. Measuring Inequality, Concentration, and Poverty [R package ineq version 0.2–13]. (2014).

[102] Website. https://doi.org/10.2307/2223319 doi:10.2307/2223319.

