# A DNA Typewriter records the cell lineage history of a mouse, from zygote to late organogenesis

**DOI:** 10.64898/2026.07.29.741625

**Published:** 2026-10-05

**Authors:** Qi Yu, Haedong Kim, Sophie Seidel, James F. Acosta-Clark, Beth K. Martin, Kyle O’Connor, Riza M. Daza, Molly Gasperini, Jenny F. Nathans, Maggie Lam, Elena Gamo, Shruthi VijayKumar, Lauren Kuo, Jean-Benoît Lalanne, Kamen P. Simeonov, Marion Pepper, Cole Trapnell, Jesse M. Gray, Junhong Choi, Chengxiang Qiu, Jay Shendure

## Abstract

Mammalian biology unfolds over time, within tissues and organs opaque to our eyes and instruments. We applied DNA Typewriter, a sequential molecular recorder, to record the cell lineage of a mouse over nearly two weeks of development. From one embryo, we reconstruct a time-calibrated, parsimony-supported, zygote-rooted phylogeny of 1.28M transcriptionally-profiled cells. A burst of editing unequivocally marks the daughters of the first cleavage, which serve as inline replicates. We quantify clonal dominance arising during gastrulation. Tree siblings share cell type far above chance; heterotypic siblings mark terminal differentiations. Temporal sweeps of clade co-occurrence recover a dated hierarchy of cell-type couplings; imputed labels for internal nodes recapitulate known state paths. A lineage-anchored ontogeny of mammalian development, long out of reach, is coming into view.

## Full Text Availability

The license terms selected by the author(s) for this preprint version do not permit archiving in PMC. The full text is available from the preprint server.

