# Supplementary material for: In vivo reconstruction of the cell lineage history of a developing mouse with DNA Typewriter, from zygote to late organogenesis
Source: bioRxiv. 2026 Jul 30:2026.07.29.741625. Preprint. [Version 1] doi: 10.64898/2026.07.29.741625 (PMC13484211; doi:10.64898/2026.07.29.741625)
Supplement: Supplementary file 1 [file NIHPP2026.07.29.741625v1-supplement-1.pdf]

bioRxiv preprint doi: https://doi.org/10.64898/2026.07.29.741625; this version posted July 30, 2026. The copyright holder for this preprint (which was not certified by peer review) is the author/funder, who has granted bioRxiv a license to display the preprint in perpetuity. It is made available under a CC-BY 4.0 International license.

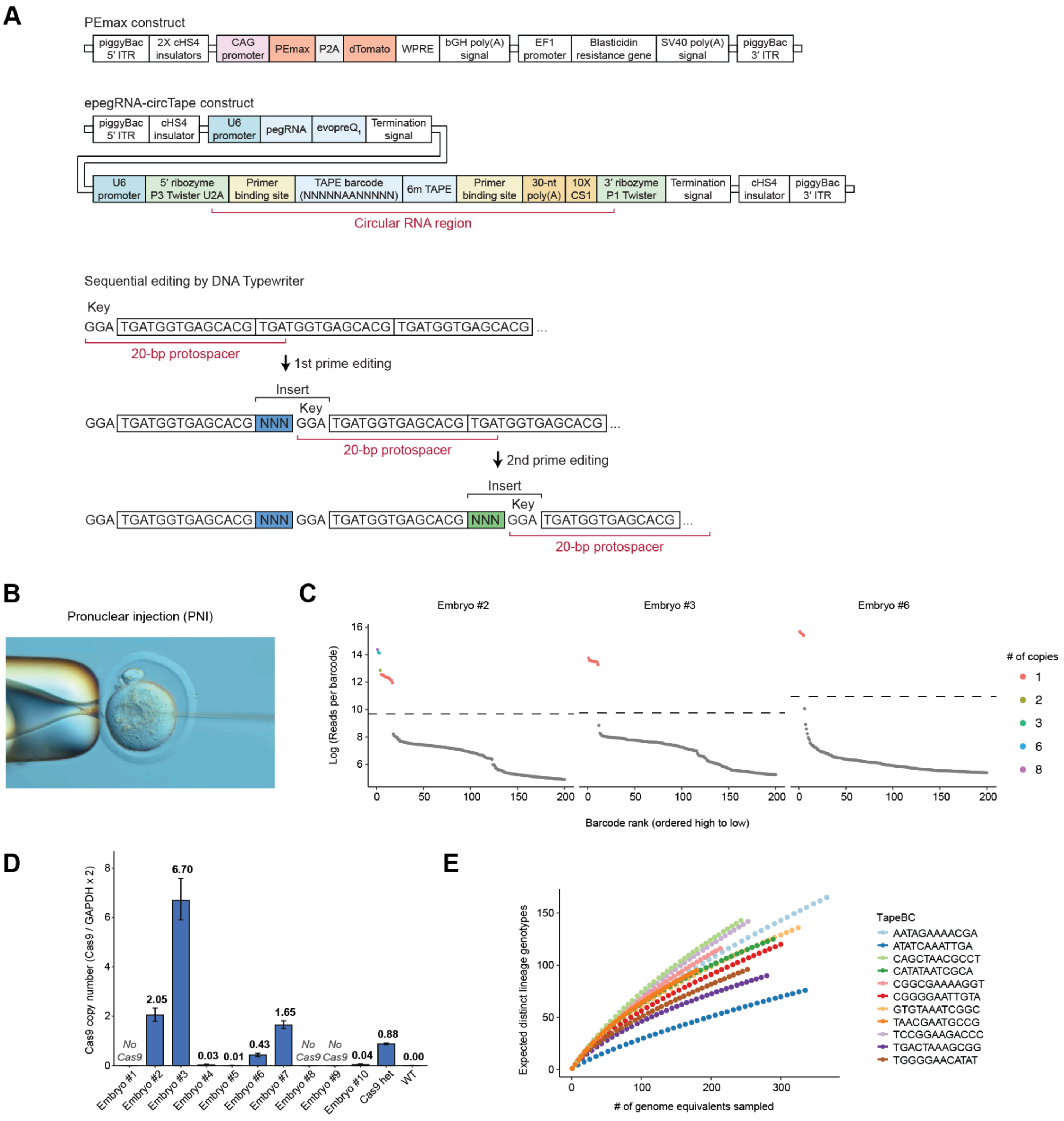

Figure S1. Characterization of epegRNA::TAPE-BC::circTAPE and PEmax integrations across E13.5 embryos, and the diversity of per-TAPE lineage genotypes in embryo #3. (A) Schematics of the PEmax and epegRNA::TAPE-BC::circTAPE constructs (top) and the sequential editing process mediated by the DNA Typewriter system (bottom). CS1, reverse complement of Capture Sequence 1 from the 10x Genomics platform. **(B)** Image of a single-cell zygote during PNI. A finely pulled glass needle was inserted into a pronucleus to deliver a mixture of mRNA and plasmid DNA. Imaged with a 40× DIC objective on a Nikon Ti2A microscope with a Nikon D1000 camera. **(C)** TAPE-BC read-count rank-abundance, shown separately for embryos #2, #3 and #6. Reads were first aggregated by

bioRxiv preprint doi: https://doi.org/10.64898/2026.07.29.741625; this version posted July 30, 2026. The copyright holder for this preprint (which was not certified by peer review) is the author/funder, who has granted bioRxiv a license to display the preprint in perpetuity. It is made available under a CC-BY 4.0 International license.

correcting each barcode to its nearest whitelist member within 2 mismatches, collapsing one- and two-base error shadows into their parent. Barcodes are ranked by read count (x-axis) against reads per barcode (log scale, y-axis); each point is one barcode, colored by estimated genomic copy number. The dashed line marks the whitelist cutoff separating called integrations (left) from background sequences (grey, right). **(D)** PEmax copy number in each E13.5 embryo was assessed by duplex ddPCR (Cas9, FAM; GAPDH, reference), calculated as (Cas9 / GAPDH) × 2. The Cas9 probe targets a region that is common to Cas9, PE2 and PEmax. Embryo #3 carried the highest estimated copy number of PEmax (measured 6.70, rounded to 7), followed by embryo #2 (measured 2.05, rounded to 2). Embryos #1, #8, and #9 yielded zero positive Cas9 droplets (“No Cas9”). Genomic DNA from a Cas9/+ mouse served as a positive control (measured 0.88, rounded to 1), and from a wildtype B6 mouse as a negative control (measured 0.00). Bars show the per-well point estimate; error bars are 95% Poisson confidence intervals propagated through the ratio. **(E)** Rarefaction analysis of recorded diversity in embryo #3. For each of the 11 TAPE-BC integrations (colored by TAPE-BC), the expected number of distinct TAPE-level genotypes (y-axis) is plotted as a function of the estimated number of genome equivalents sampled (x-axis). The diversity observed for each of the eleven TAPE integrations lies on the steeply rising portion of its curve, with a terminal slope far from zero, indicating that recorded diversity is far from saturated and is limited by the material sampled rather than by recorder capacity.

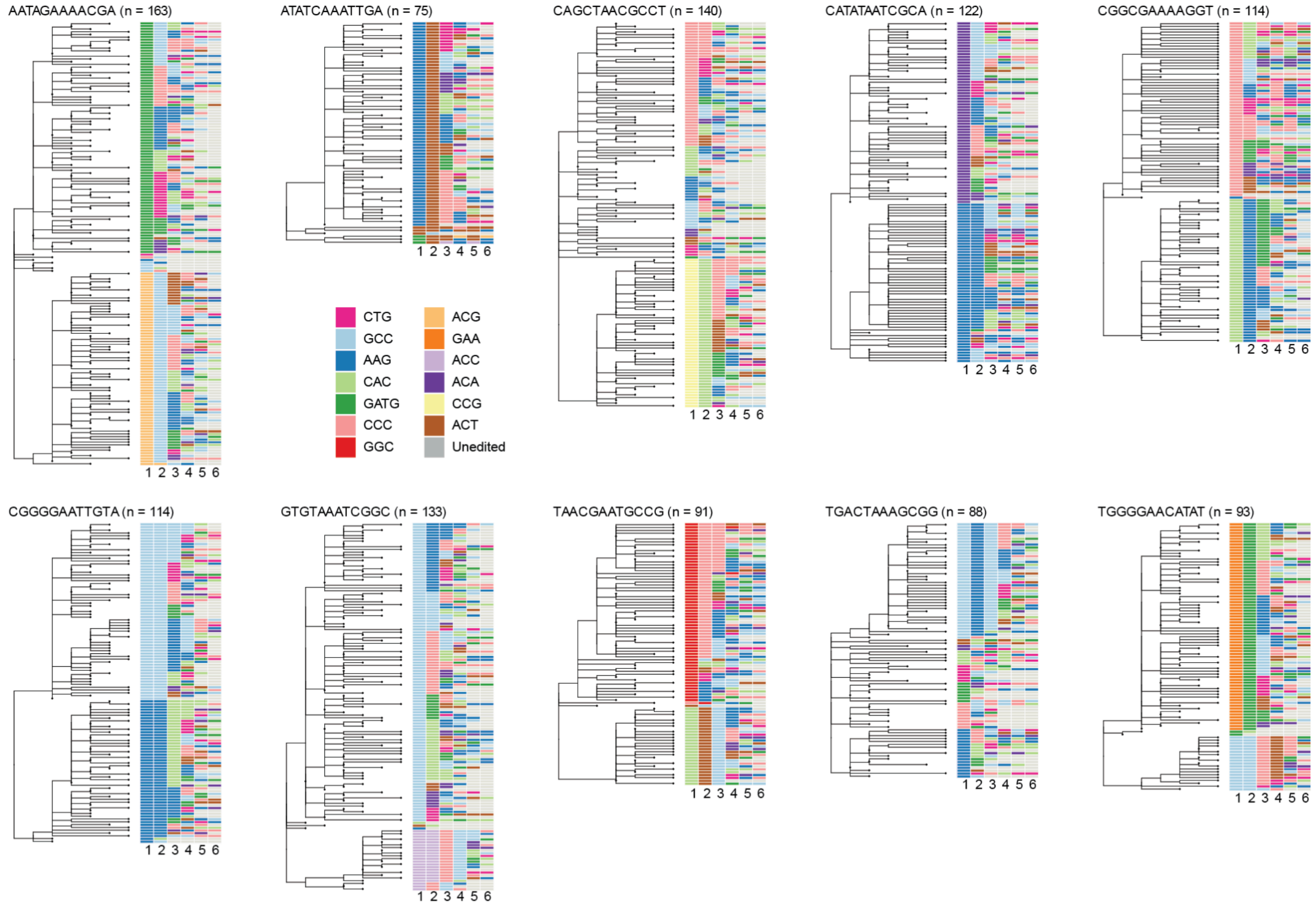

Figure S2. Neighbor-joining trees reconstructed from genotypes observed in bulk amplicon sequencing of TAPE. As in **Fig. 1F**, but for the remaining ten TAPE-BC-defined integrations. Six-site edit patterns shown to the right of lineage trees (colored by symbol; grey, unedited).

bioRxiv preprint doi: https://doi.org/10.64898/2026.07.29.741625; this version posted July 30, 2026. The copyright holder for this preprint (which was not certified by peer review) is the author/funder, who has granted bioRxiv a license to display the preprint in perpetuity. It is made available under a CC-BY 4.0 International license.

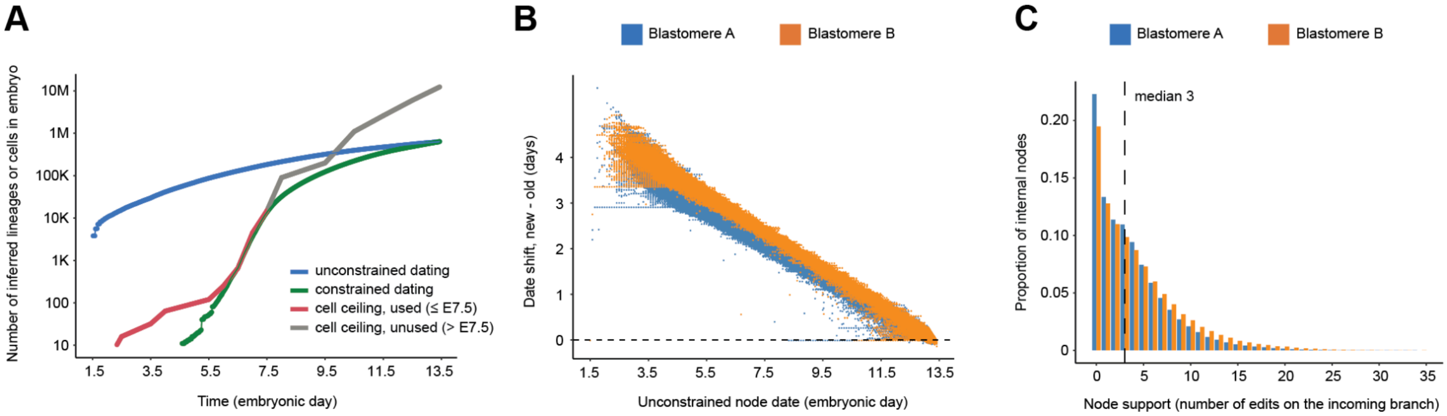

Figure S3. A cell-number ceiling corrects implausibly early node dates, and most internal branches carry multiple independent supporting edits. (A) Number of inferred lineages (blue, green) or anticipated number of cells in embryo (red, grey) over developmental time. Blue line, number of ancestral lineages for backbone tree obtained with unconstrained dating. Green line, number of ancestral lineages for backbone tree obtained with constrained dating (2-hour floor, cell number ceiling). Red/grey line, anticipated number of cells in embryo at various developmental times based on literature (**table S2**), colored differently for timepoints where the literature-based ceiling was constraining (red, ≤E7.5) vs. where it was not (grey, >E7.5). **(B)** Scatterplot of shifts in dating (y-axis, constrained minus unconstrained) vs. the unconstrained date (x-axis), for internal nodes of blastomere subtrees A (blue) and B (orange). **(C)** For each internal node, support is quantified as the number of edits accumulated on the incoming branch. A normalized histogram is shown that includes the proportion of internal nodes with various levels of support for the A (blue) and B (orange) backbone trees; dashed lines indicate the median. 78% (A) and 81% (B) of internal branches are supported by ≥1 edit. 64% (A) and 68% (B) of internal branches are supported by ≥2 edits.

bioRxiv preprint doi: https://doi.org/10.64898/2026.07.29.741625; this version posted July 30, 2026. The copyright holder for this preprint (which was not certified by peer review) is the author/funder, who has granted bioRxiv a license to display the preprint in perpetuity. It is made available under a CC-BY 4.0 International license.

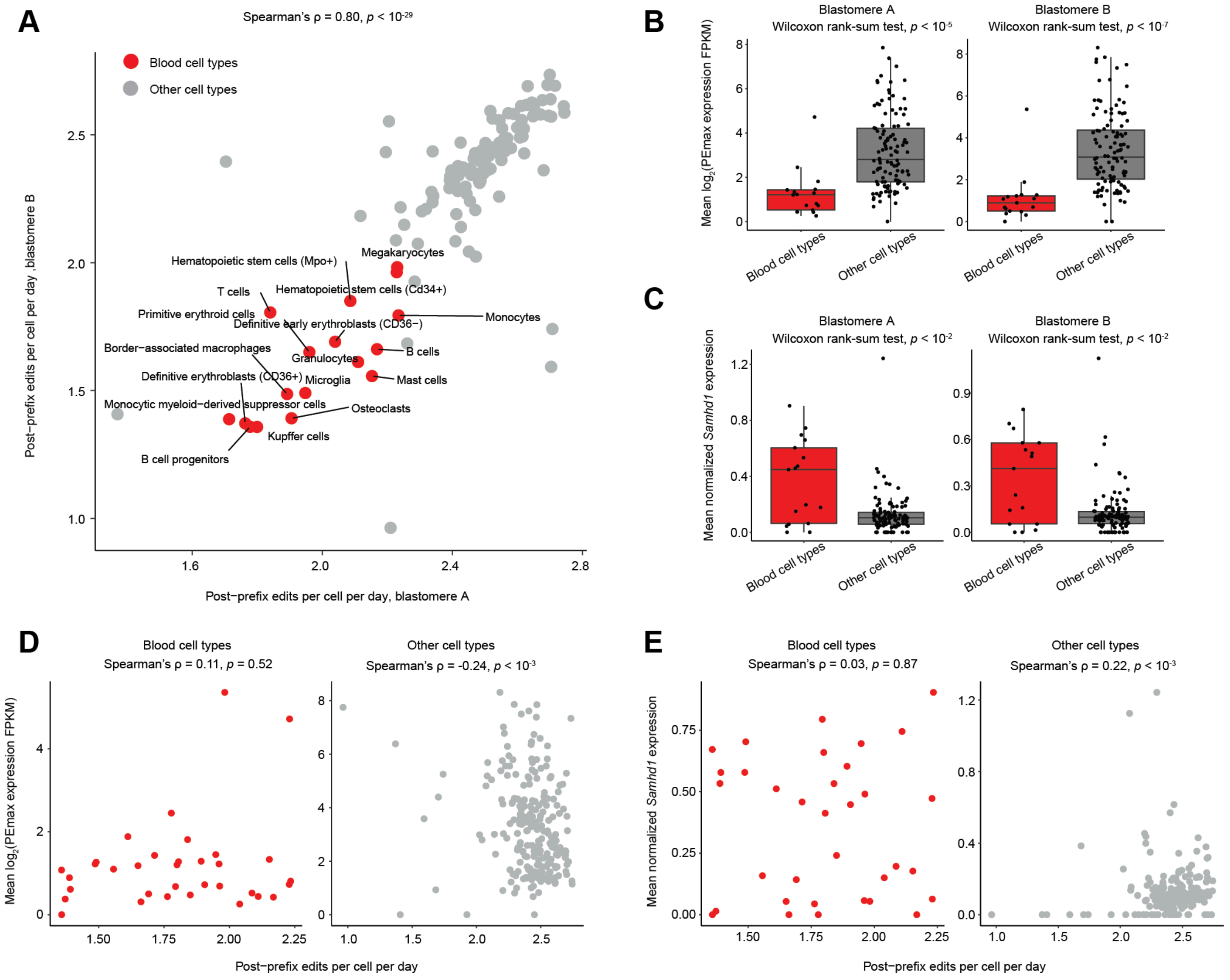

Figure S4. DNA Typewriter's cumulative activity in embryo #3 was reduced in hematopoietic cell types. (A) Scatter plot comparing per-cell-type post-prefix editing rates between the two founding blastomeres (A and B). For each cell, the number of edited sites accruing after the 2-cell-stage burst was counted for every recovered TAPE. These values were averaged across cells within each cell type to yield a per-TAPE editing rate for that cell type, divided by 13.5 to give a per-day rate, and then summed across TAPEs to give a per-cell, per-day editing rate for each cell type. Each dot represents one cell type; blood-associated cell types are shown in red and labeled, all others in grey. **(B)** *PEmax* transgene expression levels were inferred from the number of reads mapping to the model sequence of a PEmax-P2A-dTomato bicistronic construct, normalized by transcript length (8,005 bp) and by total reads per million to yield FPKM, then $\log_2$-transformed with a pseudocount of 1. For each cell type, the mean $\log_2$(FPKM + 1) was computed separately across cells assigned to blastomere A (left) and blastomere B (right). Cell types are grouped into blood-associated (red) and all others (grey). Boxes show median and interquartile range with whiskers extending to 1.5× IQR. **(C)** For each cell type, *Samhd1* expression levels were computed from single-cell transcriptomes, log-normalized with Seurat (scale factor 10,000), and averaged across cells within each cell type, separately across cells assigned to blastomere A (left) and blastomere B (right). Cell types are grouped into blood-associated (red) and all others (grey). Boxes show median and interquartile range with whiskers extending to 1.5× IQR. **(D)** Scatter plots of post-prefix editing rates for each cell type, plotted against their *PEmax* expression levels, stratified by color into blood-associated (red) and all other (grey) cell types. **(E)** Same as panel **D**, but plotting against cell types' *Samhd1* expression levels.

bioRxiv preprint doi: https://doi.org/10.64898/2026.07.29.741625; this version posted July 30, 2026. The copyright holder for this preprint (which was not certified by peer review) is the author/funder, who has granted bioRxiv a license to display the preprint in perpetuity. It is made available under a CC-BY 4.0 International license.

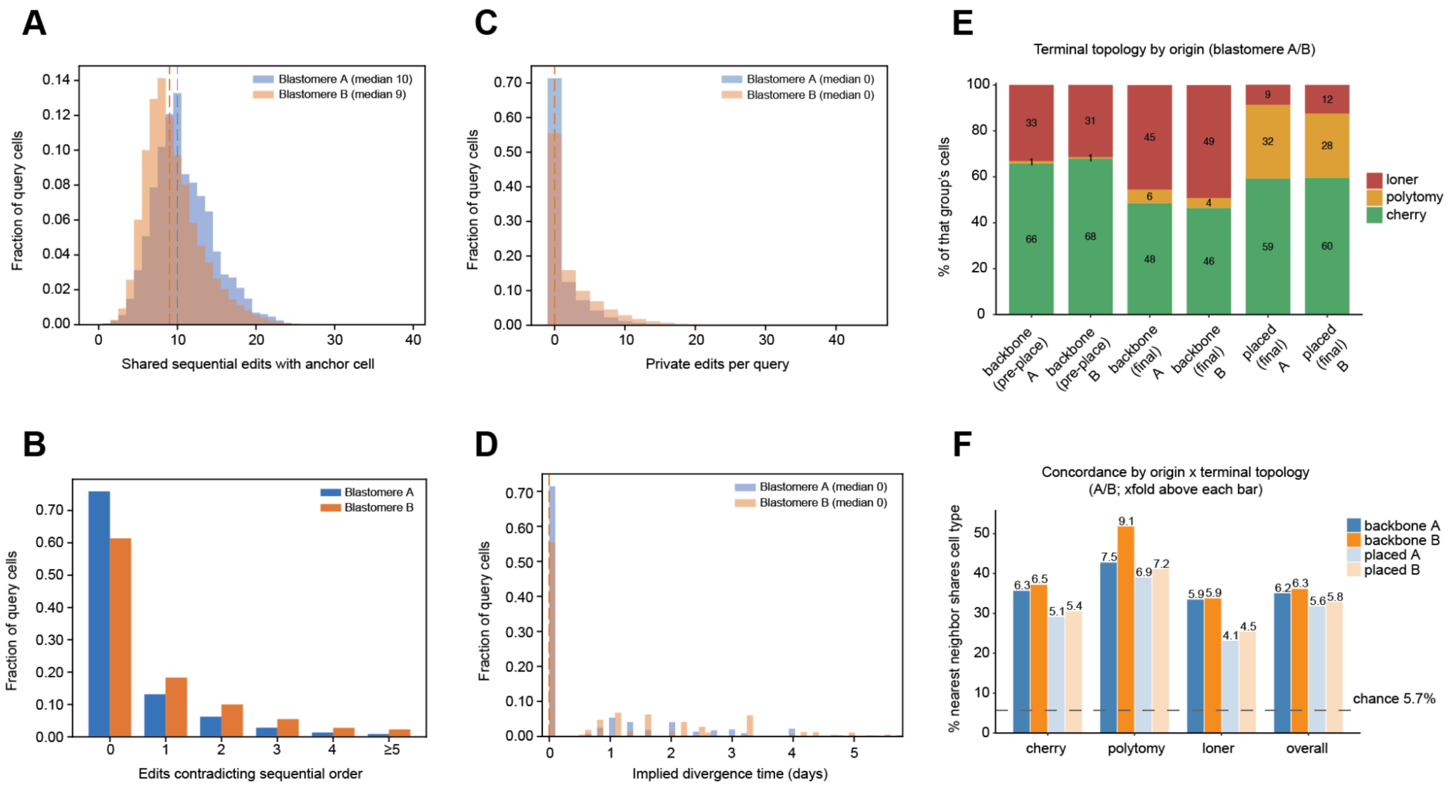

Figure S5. Query cells are placed at short edit distances from their anchors and recover cell-type concordance nearly as well as backbone cells. (A) Distribution of shared sequential edits between each query cell and its matched anchor, defined as the length of their common edited prefix summed over TAPEs genotyped in both cells. Founder-burst edits, which are shared by all cells within a blastomere, are excluded, so the values shown reflect only lineage information written after the first cleavage. **(B)** Fraction of placements, per blastomere, by the number of anchor edits that contradict the sequential edit order, *i.e.* an edit present in the anchor but absent, or differently written, in the query. Most placements are fully consistent, and placements with more than one or two contradicting edits are rare in both. **(C)** Number of private edits. Zero indicates that the query is genotype-identical to its anchor at every recovered site. **(D)** Implied divergence time between each query cell and its anchor, in days, obtained by scaling the pendant edit distance to the pair's inferred common ancestor by the per-blastomere clock rate. Most query cells are placed as contemporaries of their anchor rather than at a resolved depth of their own. The discrete spacing of the non-zero bars reflects the integer edit distances from which these times are derived. **(E)** Terminal topology by origin: cherry (exactly one leaf sibling), polytomy (≥2 leaf siblings), or loner (no leaf sibling; the parent's other children are internal subtrees). Bars give the percentage of cells in each context for, left to right, the 640,012 backbone cells in the backbone tree alone (before placement) and in the final (after placement) tree, and the 700,782 placed cells in the final tree. Each group is shown separately for blastomeres A and B. **(F)** For each cell, the fraction of its equally related nearest neighbors that share its cell type (for a cherry cell, its mate; for a polytomy or loner cell, the leaves tied for the fewest intervening branches). Each cell thus contributes a single value regardless of terminal context, making cherries, polytomies and loners directly comparable. Bars, mean across cells; dashed line, label-permutation null (5.7%, 100 permutations); value above each bar, fold-enrichment. Cell types were not used in placement.

bioRxiv preprint doi: https://doi.org/10.64898/2026.07.29.741625; this version posted July 30, 2026. The copyright holder for this preprint (which was not certified by peer review) is the author/funder, who has granted bioRxiv a license to display the preprint in perpetuity. It is made available under a CC-BY 4.0 International license.

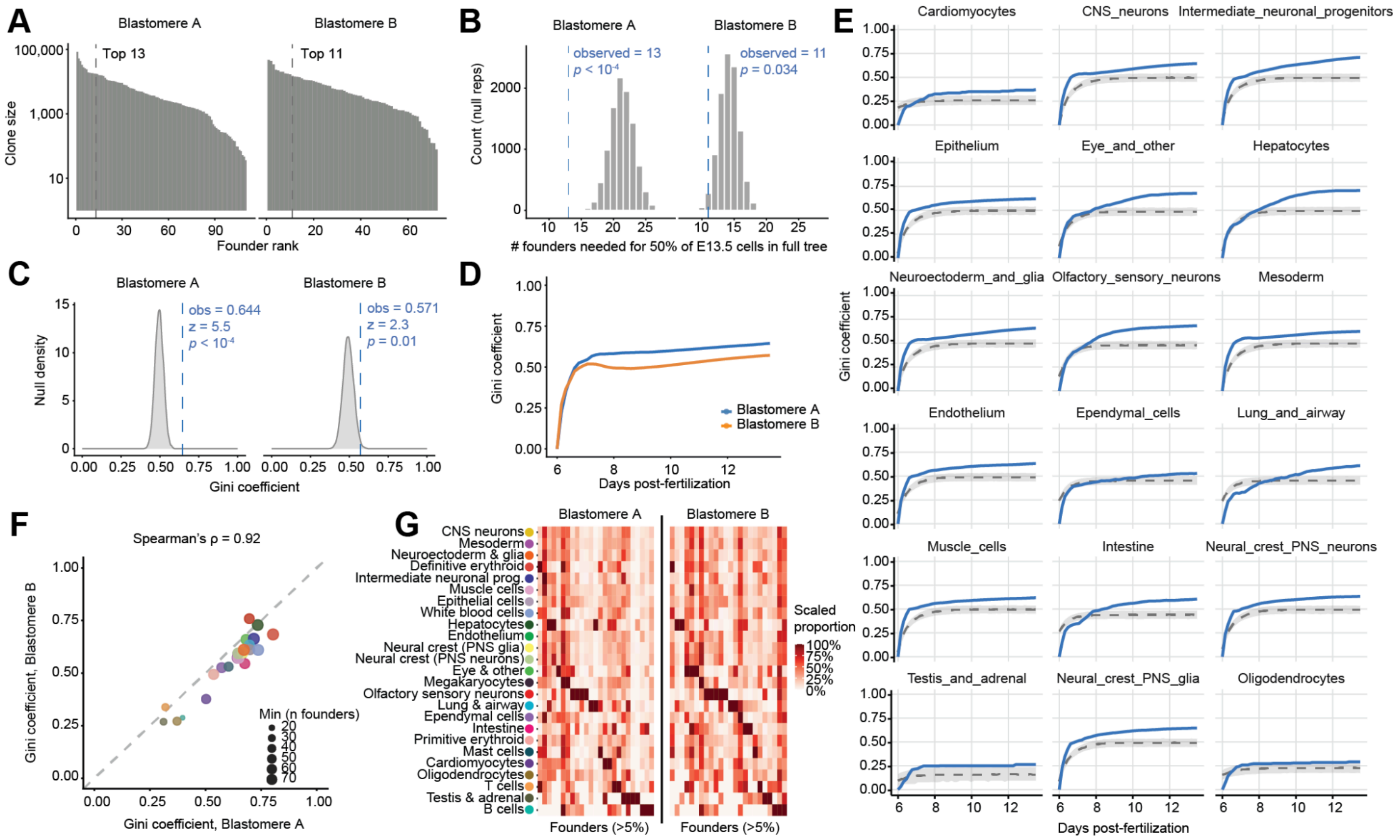

Figure S6. Patterns of clonal dominance are concordant between the two blastomere-derived subtrees. (A) Rank-abundance of founder clone sizes ($\log_{10}$ scale) for blastomeres A (110 founder clones) and B (72 founder clones). Dashed lines mark the number of founders accounting for 50% of E13.5 cells in that subtree. **(B)** Number of founders required to generate 50% of all E13.5 cells, in observed data for blastomeres A and B (blue dashed lines) vs. the Yule null (grey, 10,000 replicates). $p = P$ (null ≤ observed). **(C)** Gini coefficient of founder clone sizes, calculated separately for blastomeres A and B; the observed value (dashed line) is compared with its distribution under the Yule null (10,000 replicates). $p = P$ (null Gini ≥ observed). **(D)** Emergence of dominance, calculated separately for founders derived from blastomeres A and B. The Gini coefficient computed across founders' descendant-lineage counts at each time T, tracking the fixed founder set forward from T_0 to E13.5 (blue or orange). **(E)** As in panel **D**, but here computed for each of 18 non-hematopoietic major trajectories from founders with ≥1 descendant of that type (of 25; others shown in **Fig. 5E**), and comparing against the Yule null (grey) and its 95% envelope (grey). **(F)** Reproducibility of per-trajectory Gini indices at E13.5 compared between blastomeres A and B, for 25 trajectories in the full tree. Point size shows the smaller of the two founder counts (*i.e.* between blastomere A vs. B). Dashed line, $y = x$. Spearman's ρ = 0.92. **(G)** Heatmap showing the contribution of individual founder clones to each major trajectory, calculated separately for blastomeres A and B. See **Fig. 5F** for layout details.

bioRxiv preprint doi: https://doi.org/10.64898/2026.07.29.741625; this version posted July 30, 2026. The copyright holder for this preprint (which was not certified by peer review) is the author/funder, who has granted bioRxiv a license to display the preprint in perpetuity. It is made available under a CC-BY 4.0 International license.

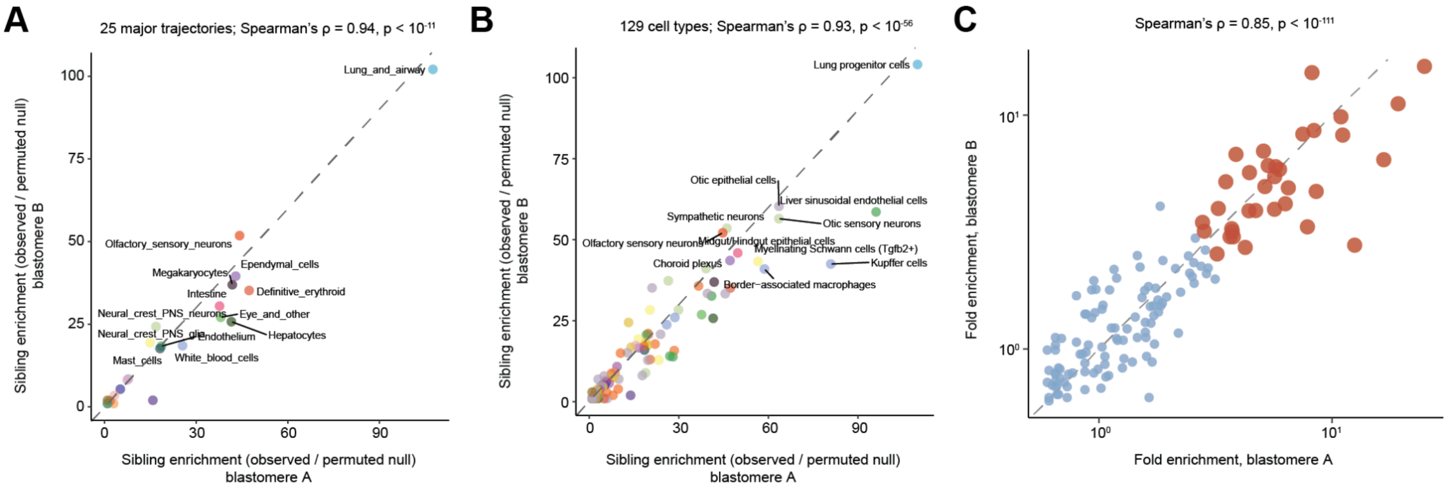

Figure S7. Tree siblings share annotations far in excess of chance, and reproducibly so. (A) Sibling enrichment for each of the 25 major trajectories, computed independently for descendants of blastomere A (x-axis) and blastomere B (y-axis). Enrichment is the ratio of same-trajectory sibling pairs to the mean count under a null that shuffles major trajectory labels across tips within A/B-defined half-embryo subtrees, holding sibling structure fixed (100 permutations), with a pseudocount of 1 in numerator and denominator to stabilize rare fates. Each point is one major trajectory, colored as in **Fig. 2A**; dashed line, $y = x$. Trajectories with the highest mean enrichments are labeled. Spearman's $\rho = 0.94$, $p < 10^{-11}$. Values for all trajectories are given in **table S3**. **(B)** As in panel **A**, but computed at cell-type resolution across 129 cell types present in both the A and B subtrees. Spearman's $\rho = 0.93$, $p < 10^{-56}$. Values for all cell types are given in **table S4**. **(C)** Fold-enrichment of each progenitor→post-mitotic pairing computed separately for A and B, for the 406 of 653 evaluated pairings observed in both subtrees ($\log_{10}$-scale axes; dashed line, $y = x$). Red, 34 reliable progenitor→post-mitotic pairings that clear BH FDR<1% and are ≥3-fold enriched in the overall tree (*i.e.* the subset shown in **Fig. 6A**). Blue, the remaining 372 pairings. Spearman's $\rho = 0.85$, $p < 10^{-111}$, when calculated on all 406 pairings shown.

bioRxiv preprint doi: https://doi.org/10.64898/2026.07.29.741625; this version posted July 30, 2026. The copyright holder for this preprint (which was not certified by peer review) is the author/funder, who has granted bioRxiv a license to display the preprint in perpetuity. It is made available under a CC-BY 4.0 International license.

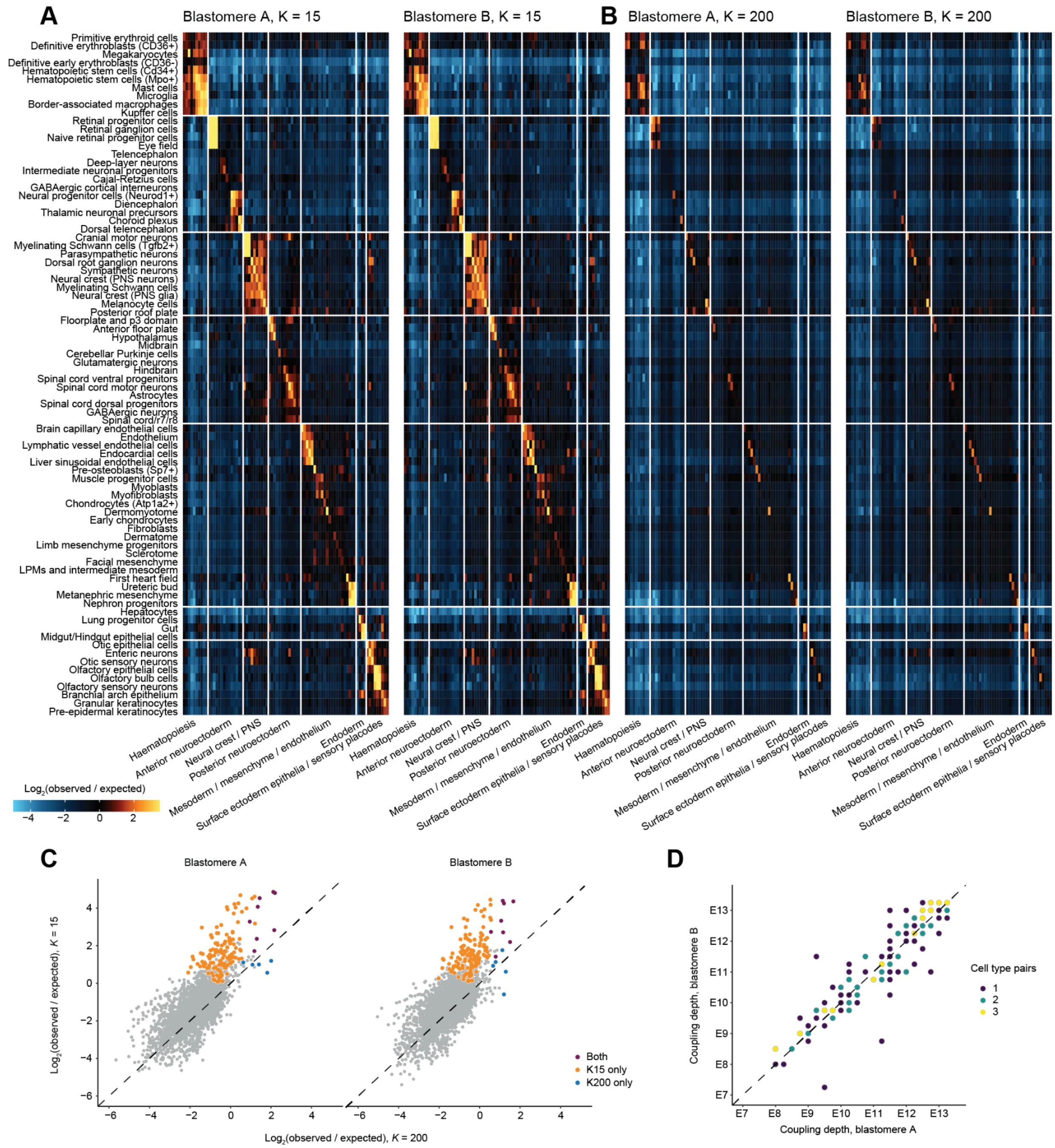

Figure S8. Clade co-occurrence analyses yield consistent patterns when conducted independently on subtrees defined by blastomeres A and B. (A) Timed fate couplings between cell types are reproducible between A and B subtrees at K = 15. Details as in **Fig. 6B**, but here calculated on A and B subtrees independently. **(B)** Timed fate couplings between cell types are reproducible at K = 200 as well, but show weaker block structure than K = 15. Details as in **Fig. 6**B, but here calculated on the blastomere A and B subtrees independently and at K = 200 instead of K = 15. **(C)** Co-occurrence enrichment at recent vs. ancient clade scales is highly reproducible. Each point is one of the 3,321 pairs of the 82 cell types, plotted by its $\log_2$ enrichment at K = 200 (x) against K = 15 (y), with results based on blastomere A and blastomere B's subtrees shown as separate panels. Color indicates significance (observed / expected ≥3 permutation standard deviations) in A, B or both. Dashed line, $y = x$. Most pairs that reach significance do so only at K = 15 and lie well above the identity line, *i.e.* their co-occurrence is specific to recent clades. **(D)** For each of the 128 significantly coupled cell-type pairs, coupling depth was re-estimated independently in blastomere A (x-axis) and B (y-axis). Because both axes are quantized to the 0.25-days, points are colored by the number of pairs falling on each grid position. Dashed line, $y = x$.

bioRxiv preprint doi: https://doi.org/10.64898/2026.07.29.741625; this version posted July 30, 2026. The copyright holder for this preprint (which was not certified by peer review) is the author/funder, who has granted bioRxiv a license to display the preprint in perpetuity. It is made available under a CC-BY 4.0 International license.

SUPPLEMENTARY INFORMATION

Table S1. Ubiquitous edits identifying the two blastomeres of the first cell division.

Table S2. Estimated total cell number during mouse embryonic development.

Table S3. Log2-fold-change of same-major-trajectory sibling pairs versus label-shuffled null.

Table S4. Log2-fold-change of same-cell-type sibling pairs versus label-shuffled null.

Table S5. List of postmitotic cell types.

Table S6. Coupling-depth matrix across 76 cell types.

Table S7. Qualifying traceback paths, with supporting cell counts and blastomere reproducibility.

File S1. Oligonucleotides used in molecular cloning, amplicon sequencing, and ddPCR.

File S2. Oligonucleotides used in sci-RNA-seq3.
